# Supplementary material for: Predicting sequence-specific amplification efficiency in multi-template PCR with deep learning
Source: Nat Commun. 2025 Oct 16;16:9187. doi: 10.1038/s41467-025-64221-4 (PMC12533003; doi:10.1038/s41467-025-64221-4)
Supplement: Supplementary file 1 — Supplementary Information [file 41467_2025_64221_MOESM1_ESM.pdf]

Supplementary Notes

Supplementary Note 1: Details of the deep learning models

- **Input:** One-hot encoded DNA sequences x.
- $x \leftarrow$ Linear projection(x) + Positional embedding(x)
- For $i = 1, \cdots, N$:
  - $x \leftarrow$ 1D convolutional layer(x)
  - $x \leftarrow$ BatchNorm(x)
  - $x \leftarrow$ ReLU(x)
- $x \leftarrow$ Global pooling(x)
- $x \leftarrow$ Linear classification layer(x)

Detailed architecture of the 1D-CNN model with PE, where N is the total number of convolutional layers.

Supplementary Note 2: Additional baseline models

We conducted a series of experiments to evaluate additional baseline models beyond the proposed baseline models mentioned in the main text. These baselines fall into three categories:

Oversampling for Class Imbalance.

To address concerns about the classifier's ability to handle severe class imbalance (2% prevalence of low-efficiency sequences), we applied SMOTE-based oversampling to the training set during both internal and external validation. While this approach aimed to enhance the model's sensitivity to the minority class, it consistently led to decreased generalization performance. In particular, the AUPRC dropped to below 20% compared to ∼40% for the original model trained on the imbalanced data. These results, shown in Figure 28, suggest that oversampling may distort the underlying data distribution in this setting, echoing observations in prior work.[1]

k-mer and Position-Aware Feature-Based Models.

We further compared the CNN to more expressive models based on explicit k-mer feature representations. Specifically, we extracted sequence features using 3-, 4-, and 5-mer counts, with encoding the start position of each k-mer. These features were used to train LightGBM classifiers. Among them, k=4 performed best; however, all k-mer-based models were still significantly outperformed by the CNN (see Figure 27). These results indicate that while position-aware k-mer features offer some predictive signal, they fall short of capturing the complex sequence patterns learned by the CNN.

Regression-Based Efficiency Prediction.

We also evaluated a regression formulation that directly predicts amplification efficiency. Despite achieving low RMSE values (e.g., ∼0.008 in the internal validation for GC data), regression models exhibited very low R^2 scores (e.g., $R^2 = 0.018$ for `GCall` and $R^2 = 0.005$ for `GCfix`) and failed to rank sequences effectively (e.g., rank correlation = −0.014 for `GCall` and −0.004 for `GCfix`), indicating limited ability to explain or rank variation in the data. This is visualized in Figure 29, where predictions are heavily clustered around the mean. Furthermore, when regression outputs were transformed into probability scores for binary classification (via inverse min-max scaling and post-hoc calibration), via

$$p = 1 - \frac{\text{pred} - \min(\text{pred})}{\max(\text{pred}) - \min(\text{pred})},$$

their AUPRC remained substantially lower than the CNN classifier (see green curves in Figure 29). These findings are consistent with prior work,[2] which suggests classification objectives may yield better optimization and generalization behavior for similar tasks.

Taken together, these baseline comparisons highlight the effectiveness of our classification approach and the superior performance of the CNN architecture in identifying poorly amplifying sequences.

Supplementary Note 3: Design and analysis of parameter validation pool

To assess the reproducibility of our assignment of amplification efficiencies, we conducted a serial amplification experiment with a new oligo pool comprising 1000 selected sequences from the GCall and GCfix pools (see Methods). The selection of sequences was primarily based on the sequences' estimated amplification efficiencies (i.e., 400 sequences with poor, 200 with normal, and 400 with superior efficiency), however, secondarily, the pool was also used to compare two methods for estimating amplification efficiencies from the sequencing data.

Specifically, of the 400 sequences with either poor or superior efficiency, half of each were selected based on the simple model of exponential amplification presented in the main text (see Equation 1 and 2). The other half was selected based on a more complex model of the whole process, including dilution and sequencing steps.

Supplementary Fig. 20b shows how the relative coverage of all sequences evolves during serial amplification. Evidently, both methods for estimating amplification efficiencies successfully identify poorly amplifying sequences, as the sequences identified by either model are quickly depleted in the sequencing data (i.e., around cycle 60). The simple model (model 1 in the figure) appears to perform slightly better in identifying poorly amplifying sequences compared to the complex model (model 2 in the figure), as evidenced by the faster loss of its selected sequences in the sequencing data (i.e., between cycles 30 and 60). For this reason, as well as the simplicity of this model, we

selected the method using a simple exponential model of the amplification process as our method for estimating the amplification efficiencies in all analyses.

Accordingly, we limited the sequences shown in Fig. 2e and Supplementary Fig. 21 to those selected by the simple model described in the main text, omitting the sequences selected by the complex model entirely. This reduces the number of sequences in the analysis from 1,000 to 654.

Supplementary Note 4: Complex statistical model

We introduce a statistical model to account for the concentration distribution in each step of the proposed sequencing experiment, and this model is modified based on the statistical model introduced in Ref. [3] for RNAseq. In this model, each DNA present in the original preparation, whose level is indicated as N_i, where i is the index of the individual sequence, and since the abundance of each sequence is the same in all replicated samples, the sample index j is omitted. Then each sample was subjected to different sequences of combinations of dilution steps and PCR amplification steps. The PCR amplification steps are made up of blocks of 15 cycles each, and the dilution steps are interleaved between the PCR steps to reduce the impact of the PCR bias.

Dilution:

As the dilution steps will simply dilute the concentration of the DNA molecules (the amount of DNA per unit volume) in a linear manner, so these separate dilution steps can simply be multiplied. The mean and variance of the concentration X_i can be formulated as:

$$E[X_{i,j}] = d_j N_{i,j}, \tag{1}$$

$$Var[X_{i,j}] = d_j^2 N_{i,j}, \tag{2}$$

where d_j is the multiplication of all dilution steps subject to each individual sample j.

PCR amplification:

The accumulation of products during the PCR reaction may be stochastically modeled by a Galton-Watson (GW) branching process[4–7]. We will consider the exponential phase of the PCR reaction, in which the reaction efficiency is constant in all cycles and also in different samples j for the same sequence i. In particular, the abundance of DNA sequence i after the $n+1$ PCR cycle can be expressed by the Markovian relation[7]:

$$L_i^{n+1} | L_i^n, \epsilon_i = L_i^n + Bionmial(L_i^n, \epsilon_i). \tag{3}$$

So, the abundance of DNA sequence i after the $n+1$ PCR cycle, L_i^{n+1}, is only conditioned on the abundance of DNA sequence i after the n PCR cycle and the PCR efficiency of the sequence, ϵ_i. Despite the analytical intractability of the GW distribution $P(L_i^n|X_i, \epsilon_i)$, the relationship between the mean and variance can be explicitly derived by standard branching theory results[4,7] as:

$$E[L_{i,j}] = N_{i,j}(1+\epsilon_i)^{N_j^{PCR}}, \tag{4}$$

$$\begin{aligned} Var[L_{i,j}] &= N_{i,j}\frac{1-\epsilon_i}{1+\epsilon_i}(1+\epsilon_i)^{N_j^{PCR}}[(1+\epsilon_i)^{N_j^{PCR}}-1] \\ &\approx N_{i,j}\frac{1-\epsilon_i}{1+\epsilon_i}(1+\epsilon_i)^{2N_j^{PCR}}. \end{aligned} \tag{5}$$

Sequencing Run:

For simplicity of the statistical model, we use a single parameter p to represent the probability that a given molecule existing in the PCR amplified library will generate a sequence entry in the output of the sequence. This probability encapsulates signal loss due to many subfactors in the sequencing run, e.g. sequencing depth, library preparation efficiency, sequencing technology efficiency, etc. The whole sequencing step can be viewed as a Poisson process where the final sequncing reads are ditributed according to the Poisson distribution. However, as discussed in the previous paragraphs, $L_{i,j}$ is a random variable, and this makes the Poisson process a mixed Poisson process which is a convolution of two distributions: the distribution of $L_{i,j}$ and the Poisson distribution defined by the sequencing probability p, and this yields the following statistical model for the final sequencing reads per sequence per sample $Y_{i,j}$:

$$Y_{i,j}|p, L_{i,j} \sim Poisson(pL_{i,j}), \tag{6}$$

$$\begin{aligned} E[Y_{i,j}] &= E[E[Y_{i,j}|L_{i,j}, p]] = pE[L_{i,j}] \\ &= p(1+\epsilon_i)^{N_j^{PCR}} d_j N_i, \end{aligned} \tag{7}$$

$$\begin{aligned} Var[Y_{i,j}] &= Var[E[Y_{i,j}|L_{i,j}, p]] + E[Var[Y_{i,j}|L_{i,j}, p]] \\ &= E[Y_{i,j}](E[Y_{i,j}]\phi + 1), \end{aligned} \tag{8}$$

$$\phi_{i,j} = \frac{(1-\epsilon_i)}{E[X_{i,j}](1+\epsilon_i)}. \tag{9}$$

The variance of the mixed Poisson distribution is determined by the law of the total variance:

$$Var(X) = E[Var(X \mid Y)] + Var(E[X \mid Y]). \tag{10}$$

In addition, extensive numerical simulations have been conducted to assess whether the mixed Poisson distribution by less complex functions in this study[3]. The authors proved that the Linear Quadratic Normal family (LQNO) can be used as a less complex alternative to approximate the mixed Poisson distribution, which is defined as:

$$Y_{i,j} \sim LQNO(\mu_{i,j}) = \frac{exp(-(Y_{i,j} - \mu_{i,j})^2/2\sigma_{i,j}^2}{\sqrt{2\pi}\sigma_{i,j}}, \tag{11}$$

$$\sigma_{i,j}^2 = \mu_{i,j}(1 + \mu_{i,j}\phi_{i,j}). \tag{12}$$

The overall statistical model is optimized by minimizing the negative log-likelihood (NLL), which quantifies the difference between the model predictions and the observed data (i.e. the final sequencing reads $Y_{i,j}$). The optimization algorithm iteratively adjusts the model parameters to minimize the objective function, and this process involves evaluating the objective function and its derivatives at various points in the parameter space.

As this complex statistical method did not perform better than a two parameter model (see Methods section and Supplementary Figure 20), the two parameter model was chosen for all further analysis.

Supplementary Note 5: Primer3 experiments

The experiments to validate the generalization of the identified inhibition mechanism were based on an amplicon designed by Primer3Plus[8]. To show generalization, special care was taken to use novel adapter sequences without any bias or internal secondary structure. Specifically, a randomly generated sequence of 1000 nt was generated and used as the template sequence in Primer3. Then, using the ”qPCR” settings and a product size range adjusted to 60-80, Primer3Plus selected an amplicon (63 nt) and corresponding primer sequences (20 nt both, see Supplementary Fig. 31 and Supplementary Table 6). Two additional sequences were created based on this amplicon selected by Primer3Plus, by manually introducing motifs complementary to the 5’- and 3’-adapters respectively. Specifically, a 6 nt motif complementary to either adapter was introduced just downstream of the adapter, once at the 5’- and once at the 3’-adapter (see Supplementary Fig. 31 and Supplementary Table 6). This recreates the motif-dependent sequence structure identified in the main text, but with a completely different primer set.

All three sequences were then individually synthesized (Microsynth, desalted) and used – together with the corresponding primer set – in three amplification experiments. First, amplification efficiency was quantified using serial dilutions with qPCR, as in Fig. 2d and outlined in the Methods

of the main text (i.e., KAPA SYBR FAST qPCR Master Mix, 1 µL 10 µM forward and reverse primer). The results of this qPCR dilution experiment are shown in Supplementary Fig. 31 and reproduce the findings of Fig. 2d: the sequences with an introduced motif amplified significantly lower than the motif-free sequence initially selected by Primer3Plus.

Second, a melting curve analysis was performed for all three sequences after amplification for 15 cycles, using the High-resolution melting curve of the Roche LightCycler 480 II and the same setup as above (i.e., KAPA SYBR FAST qPCR Master Mix, 1 µL 10 µM forward and reverse primer). Supplementary Fig. 32 shows the melting peaks of the three sequences, highlighting the presence of an additional species with a higher melting temperature only for the sequences with introduced motifs. For comparison, the melting temperatures of the amplicons (via OligoAnalyzer by IDT, eu.idtdna.com/calc/analyzer) and the extended hairpins (dashed lines, via mfold[9]) were predicted. These showed good agreement with the experimentally observed melting temperatures, providing additional evidence of the presence of extended hairpins after amplification.

Third, an amplification experiment without any primers present was devised to demonstrate the amplificability due to self-priming. The proposed mechanism of hairpin extension predicts the occurrence of a fully complementary hairpin as the only double-stranded product after extension without primers. However, as the templates are single-stranded and polymerases only extend 3'-5', our hypothesis also predicts extension only for the template with a 3'-motif. Thus, the sequence with a 5'-motif, while forming a hairpin, would remain unextended. Thus, a three-step amplification experiment was designed, involving:

1. First, all three templates (5 µL as-synthesized) were individually mixed with buffer, dye, and dNTPs, (53 µL ultrapure water, 1 µL 100X SYBR Green (Invitrogen S7563), 20 µL 5X OneTaq Standard Reaction Buffer (NEB M0480), 2 µL 10 mM dNTPs (NEB N0447), then 19 µL per well) and underwent a first melting curve analysis.
2. Second, a polymerase was added to each well (1.25 µL OneTaq polymerase (NEB M0480) in 8.75 µL ultrapure water, then 1 µL per well), and fluorescence was observed during a prolonged extension phase (54 °C for 3 min).
3. Third, another melting curve analysis was performed.

In Supplementary Fig. 33, the time profiles of fluorescence and temperature for all templates are shown. As expected, the template with 3'-motif is extended upon addition of a polymerase, despite the lack of primers. In Supplementary Fig. 34, the melting curve analyses before and after extension are shown. As expected, only the template with a 3'-motif shows a distinct melting transition after extension. These results strongly suggest the hairpin created by the 3'-motif leads to self-priming and subsequent extension by a polymerase.

Supplementary Figures

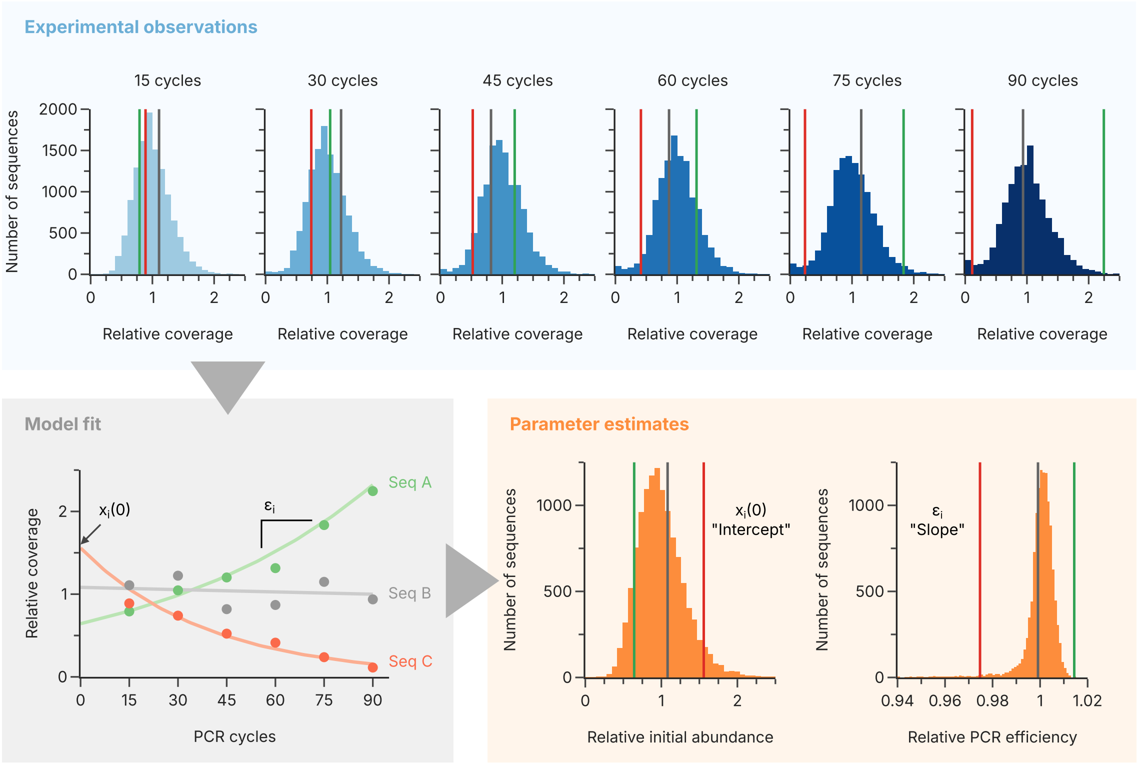

Supplementary Fig. 1 Illustration of the model workflow. After each iteration of the serial amplification protocol, the sequencing data is mapped to the reference sequences to quantify the relative coverage of each sequence in the pool (top, blue box). For each sequence, the set of relative coverages at each iteration is used to fit the two parameters of the exponential PCR model: the initial abundance (intercept, $x_i(0)$) and the amplification efficiency (slope, ϵ_i) (bottom left, gray box). The parameter estimates of all sequences in the pool yield distributions of the initial abundance and amplification efficiency (bottom right, orange box). All data shown corresponds to the GCall pool. The relative coverages and parameter estimates of three exemplary sequences (A, green, high amplification efficiency; B, gray, normal amplification efficiency; and C, red, low amplification efficiency) are highlighted across all panels. Source data are provided as a Source Data file.

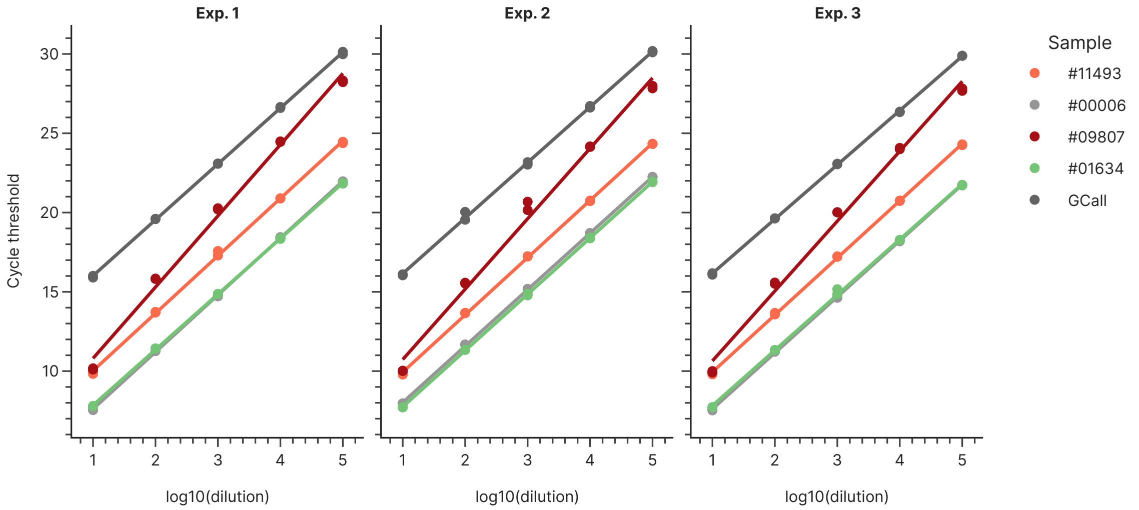

Supplementary Fig. 2 qPCR dilution curves for selected sequences of the GCall pool. Four sequences from the GCall pool were selected based on their estimated amplification efficiency (see Methods), individually synthesized, and tested for amplification efficiency together with the GCall pool as a whole. The slopes of the dilution curves across three repetitions (Exp. 1 through Exp. 3) of the dilution and qPCR workflow were then used to estimate the amplification efficiency (see Supplementary Table 2). Source data are provided as a Source Data file.

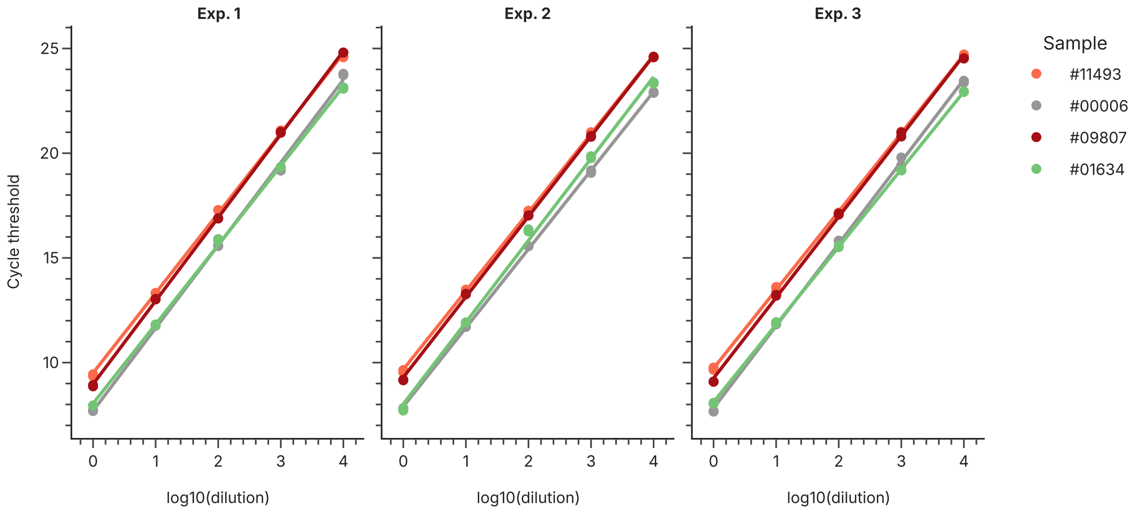

Supplementary Fig. 3 qPCR dilution curves for selected sequences of the GCall pool amplified with degenerate primers. The same sequences outlined in Supplementary Fig. 2 were amplified with a degenerate primer to estimate their amplification efficiencies (see Methods). Source data are provided as a Source Data file.

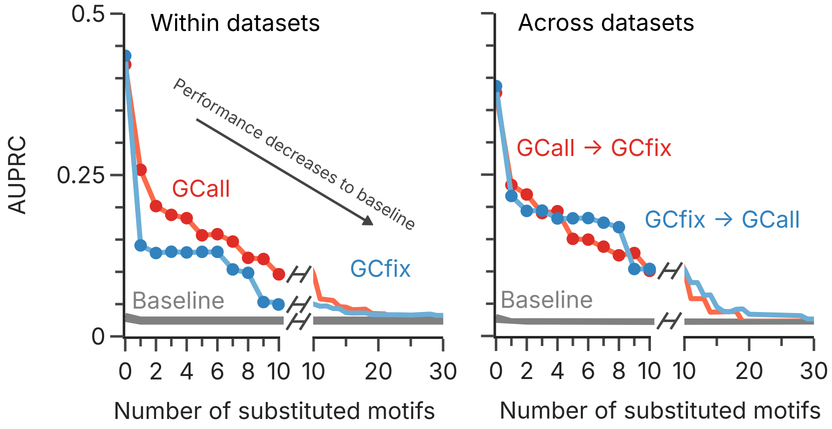

Supplementary Fig. 4 1D-CNN performance on the GCall and GCfix datasets during motif substitution using AUPRC. This figure shows the performance of the 1D-CNN models and the baseline models within (left) and across the GCall and GCfix datasets (right) as a function of the number of motifs replaced in the test data. The GCall model is shown in red, and the GCfix model is shown in blue. This figure is the equivalent to Fig. 4e using AUPRC as the performance metric. Source data are provided as a Source Data file.

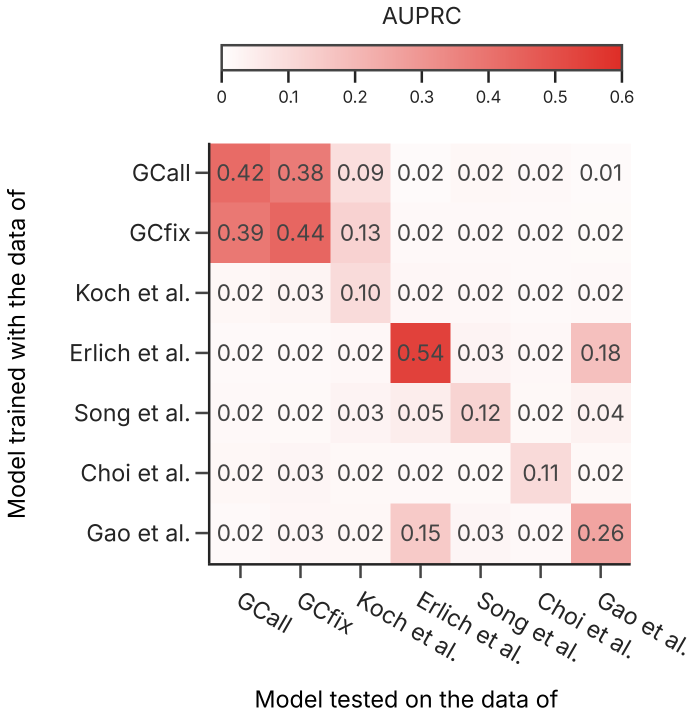

Supplementary Fig. 5 1D-CNN performance on all literature datasets using AUPRC. Heatmap of the area under the precision-recall curve (AUPRC) metric for the models trained and tested on the different literature datasets. This figure is equivalent to Fig. 5 using AUPRC as the performance metric. Source data are provided as a Source Data file.

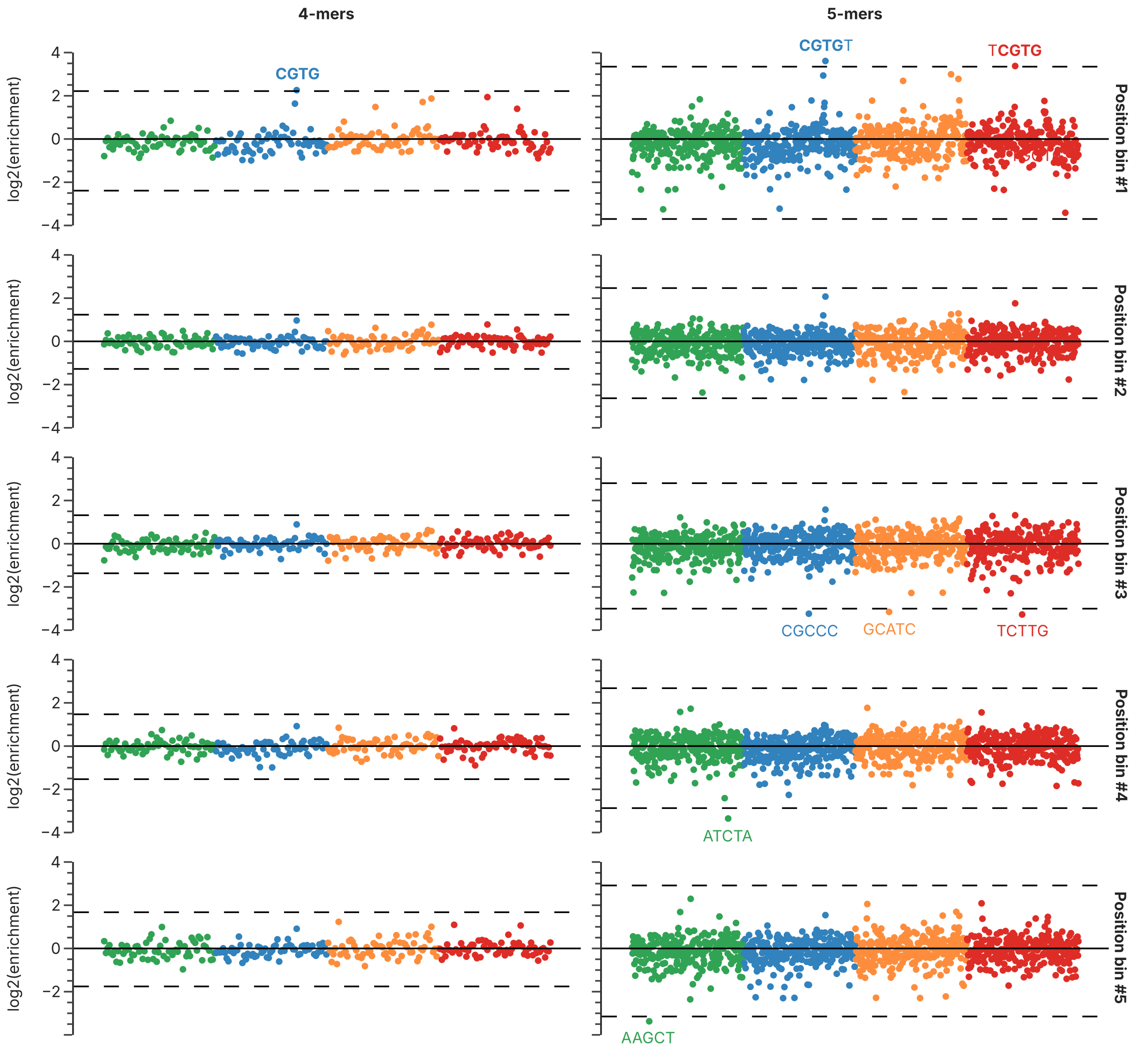

Supplementary Fig. 6 K-mer frequency analysis for the combined GCall and GCfix datasets. All sequences of the GCall and GCfix pools were converted into overlapping k-mers (considering $k \in \{4, 5\}$, left and right column respectively), and their frequency in the positive (i.e. low efficiency) group compared to their frequency in the negative group (i.e. normal efficiency) assessed. For each k-mer, the plot shows its enrichment in the positive group, separated by five positional bins starting from the 5'-end of the sequence. The solid horizontal line corresponds to an equal frequency in both groups (i.e. no enrichment), whereas the two dashed lines indicate five standard deviations (i.e. strong enrichment or depletion). k-mers outside of five standard deviations are annotated, with the CGTG submotif highlighted in bold where present. Colors indicate the starting nucleotide of the k-mer. It should be noted that the positional binning leads to a low abundance of k-mers within the poorly-amplifying sequences in each group. As a result, the significance of k-mers under-represented in poorly-amplifying sequences is low, as there is considerable stochastic noise. Source data are provided as a Source Data file.

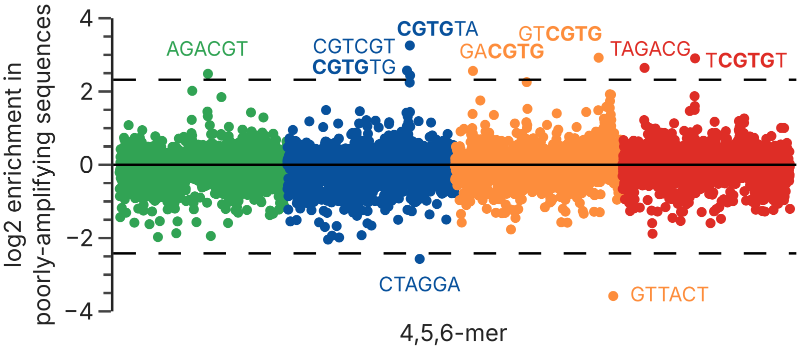

Supplementary Fig. 7 K-mer frequency analysis for the combined GCall and GCfix datasets. All sequences of the GCall and GCfix pools were converted into overlapping k-mers (considering $k \in [4, 6]$), and their frequency in the positive (i.e. low efficiency) group compared to their frequency in the negative group (i.e. normal efficiency) assessed. For each k-mer, the plot shows its enrichment in the positive group. The solid horizontal line corresponds to an equal frequency in both groups (i.e. no enrichment), whereas the two dashed lines indicate five standard deviations (i.e. strong enrichment or depletion). k-mers outside of five standard deviations are annotated, with the CGTG submotif highlighted in bold where present. Colors indicate the starting nucleotide of the k-mer. Source data are provided as a Source Data file.

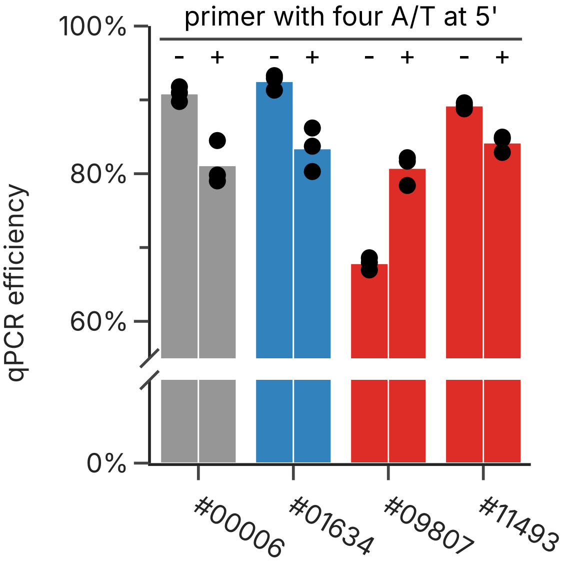

Supplementary Fig. 8 Comparison of qPCR-based amplification efficiency for the four selected sequences of the GCall pool (see Methods), upon amplification with or without degeneracy in the primer. The qPCR-based amplification efficiency ($N = 3$) is based on the slope of the calibration curves shown in Supplementary Figs. 2 and 3. Black dots show the individual data points. Source data are provided as a Source Data file.

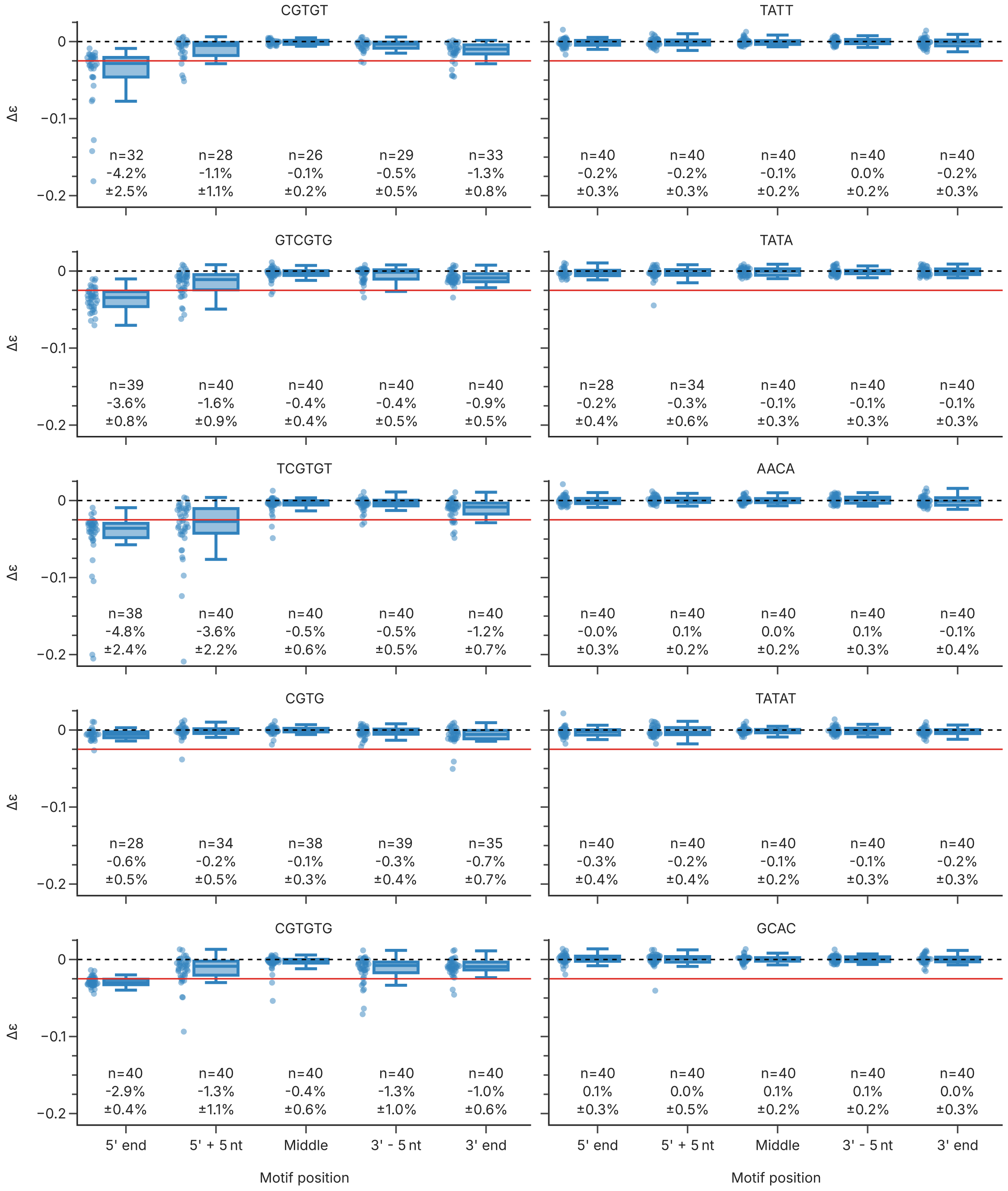

Supplementary Fig. 9 Motif- and position-dependent change in amplification efficiencies for the workflow based on the GCall and GCfix pools. Each panel shows the difference in amplification efficiency ($\Delta\epsilon$) between the base sequence without motif and the same sequence with the corresponding motif inserted at the indicated position (x-axis). All motifs in the left column correspond to the GCall/GCfix datasets, whereas the motifs in the right column belong to the Erlich et al. dataset. The dotted line represent no change in amplification efficiency, whereas the solid red line indicates a considerable change in amplification efficiency (-2.5%, roughly the threshold corresponding to the classification as a poorly amplifying sequence in the GCall/GCfix datasets). The number of individual sequences (n) and the mean difference and the Bonferroni-corrected 95% confidence interval are also shown below each box. Bounds of boxes show the first and third quartile, with the median indicated by a solid line. Whiskers show the upper and lower fence. Source data are provided as a Source Data file.

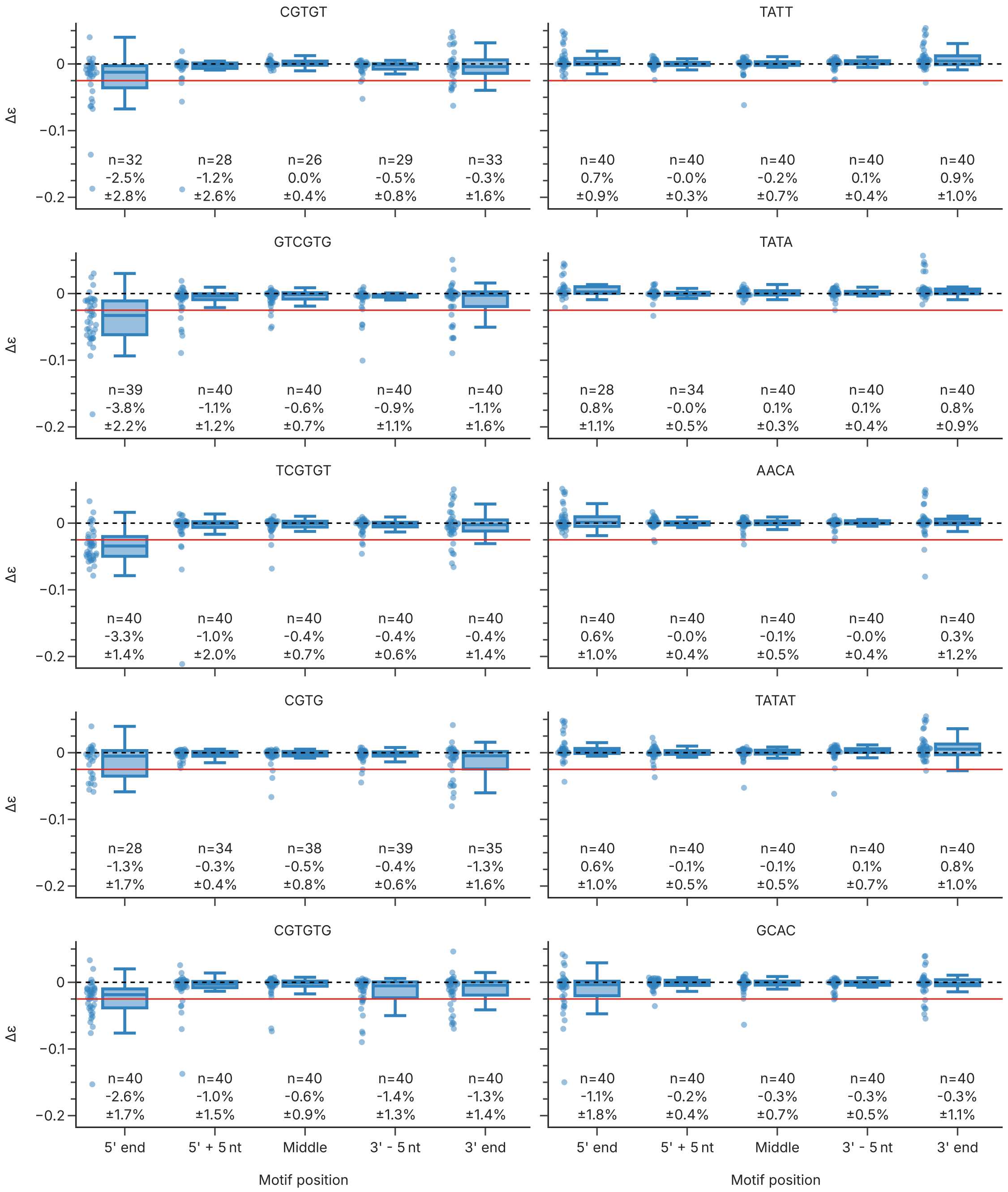

Supplementary Fig. 10 Motif- and position-dependent change in amplification efficiencies for the workflow based on the Erlich et al. data. Each panel shows the difference in amplification efficiency ($\Delta\epsilon$) between the base sequence without motif and the same sequence with the corresponding motif inserted at the indicated position (x-axis). All motifs in the left column correspond to the GCall/GCfix datasets, whereas the motifs in the right column belong to the Erlich et al. dataset. The dotted line represents no change in amplification efficiency, whereas the solid red line indicates a considerable change in amplification efficiency (-2.5%, roughly the threshold corresponding to the classification as a poorly amplifying sequence in the GCall/GCfix datasets). The number of individual sequences (n) and the mean difference and the Bonferroni-corrected 95% confidence interval are also shown below each box. Bounds of boxes show the first and third quartile, with the median indicated by a solid line. Whiskers show the upper and lower fence. Source data are provided as a Source Data file.

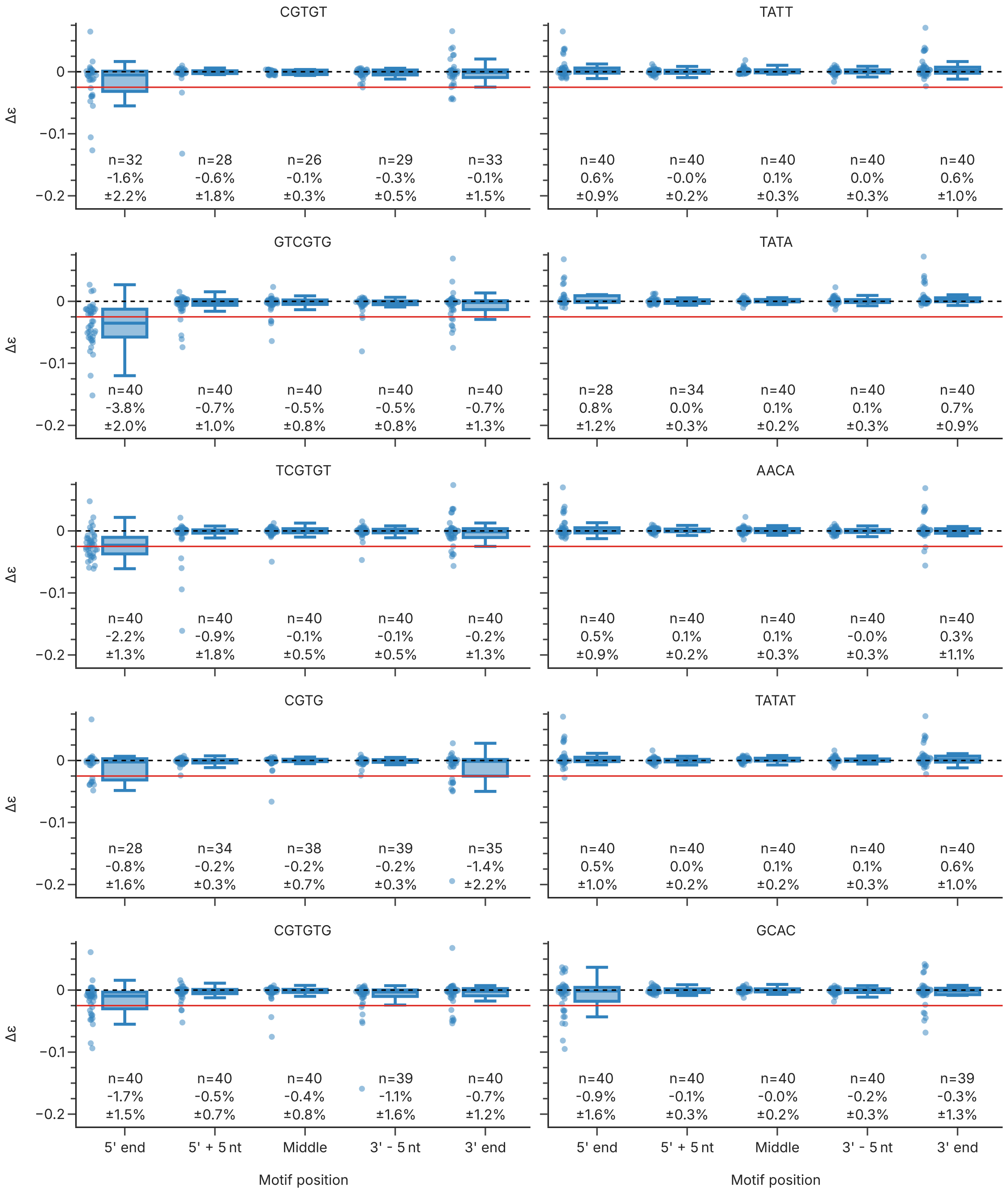

Supplementary Fig. 11 Motif- and position-dependent change in amplification efficiencies for the workflow based on the Erlich et al. pool, in the internal repeat of the experiment. Each panel shows the difference in amplification efficiency ($\Delta\epsilon$) between the base sequence without motif and the same sequence with the corresponding motif inserted at the indicated position (x-axis). All motifs in the left column correspond to the GCall/GCfix datasets, whereas the motifs in the right column belong to the Erlich et al. dataset. The dotted line represents no change in amplification efficiency, whereas the solid red line indicates a considerable change in amplification efficiency (-2.5%, roughly the threshold corresponding to the classification as a poorly amplifying sequence in the GCall/GCfix datasets). The number of individual sequences (n) and the mean difference and the Bonferroni-corrected 95% confidence interval are also shown below each box. Bounds of boxes show the first and third quartile, with the median indicated by a solid line. Whiskers show the upper and lower fence. Source data are provided as a Source Data file.

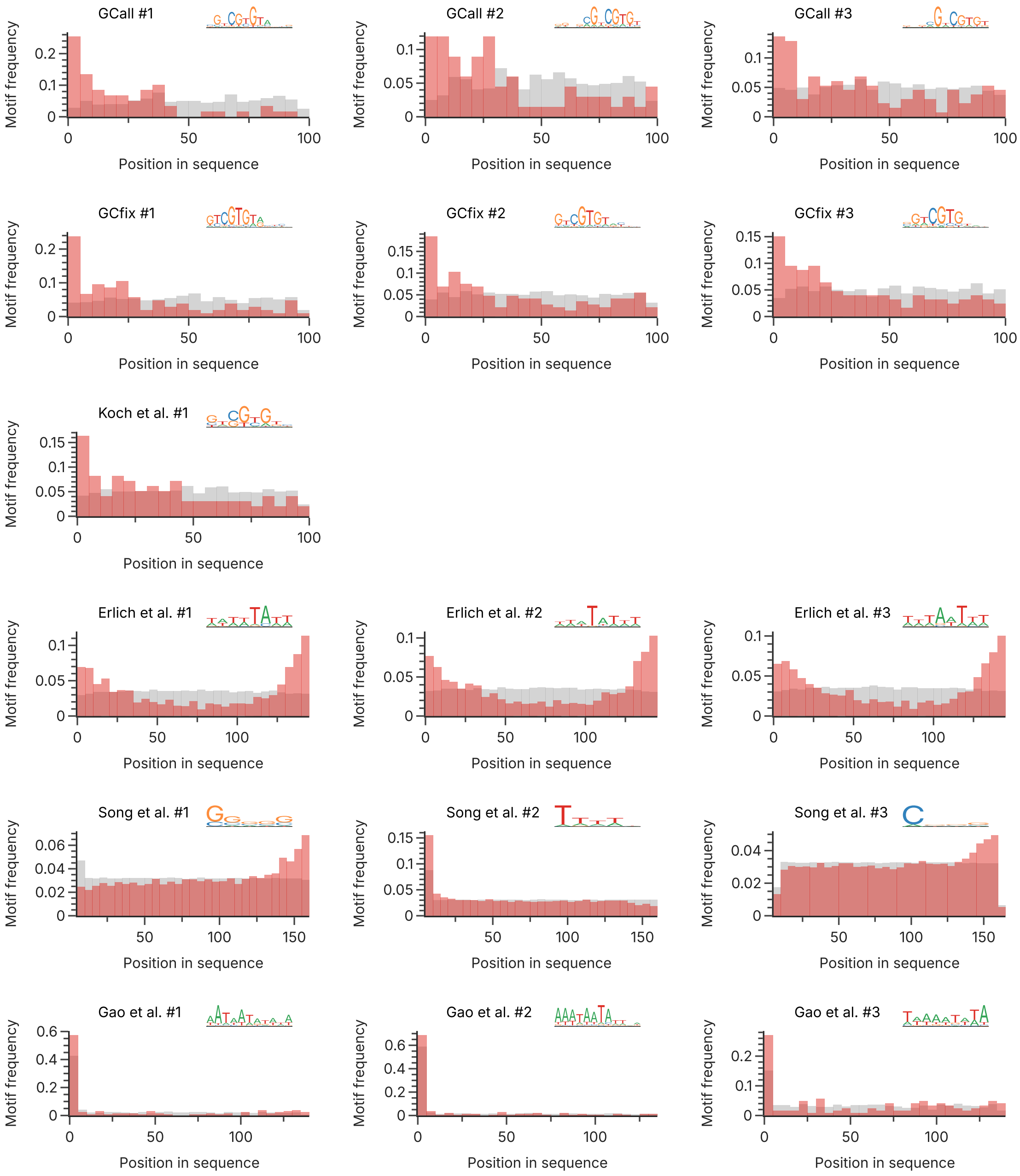

Supplementary Fig. 12 Top motifs and their positional bias for all datasets. In each row, the (up to) top three motifs of the datasets considered in this study are shown, as well as the positional frequency of this motif in the sequences classified as poorly amplifying (red) and normal (gray). Due to the length of the motif window, the number of possible positions is lower than the total length of the sequence. The Koch et al. dataset featured only one significant motif. Source data are provided as a Source Data file.

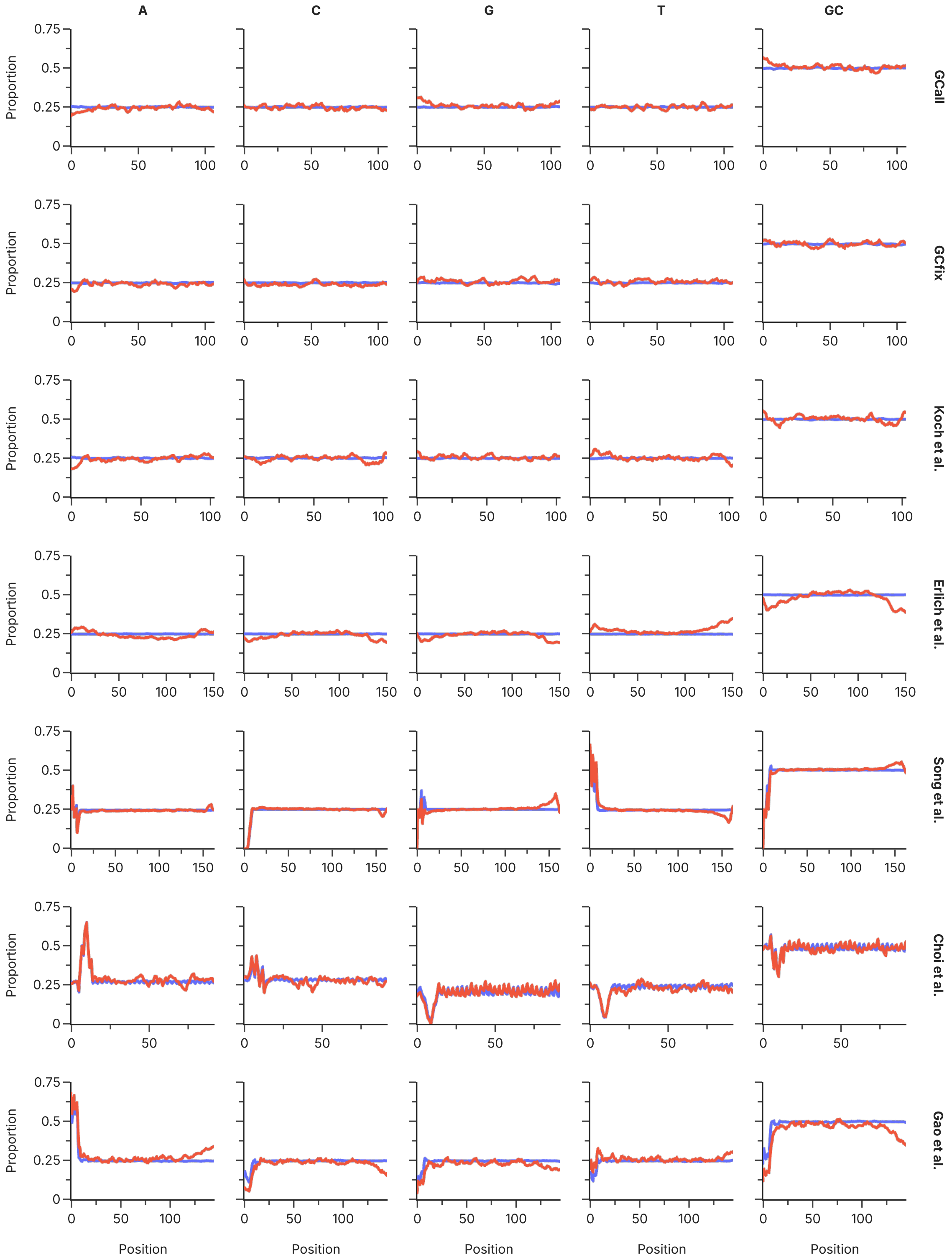

Supplementary Fig. 13 Base composition by position in the design sequences for the datasets generated in this study and the datasets from the literature. Across all columns (specifying the fraction of A, C, G, T, and GC), each row shows only the design sequences from one dataset, separating the sequences with poor (red) and normal amplification efficiencies (blue). To reduce noise, the base composition is shown with a rolling mean, employing a 5 nt window size. Source data are provided as a Source Data file.

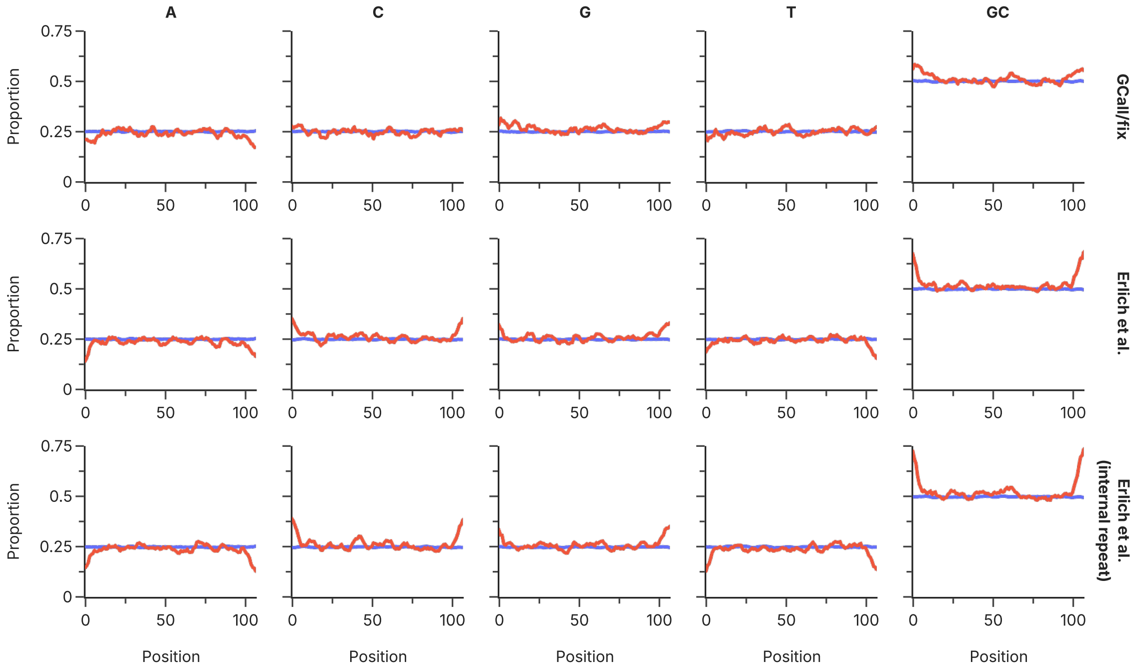

Supplementary Fig. 14 Base composition by position in the design sequences for the external validation datasets in the different experiment conditions. Across all columns (specifying the fraction of A, C, G, T, and GC), each row shows the same set of design sequences, but each time separated by poor (red) and normal amplification efficiencies (blue). To reduce noise, the base composition is shown with a rolling mean, employing a 5 nt window size. Source data are provided as a Source Data file.

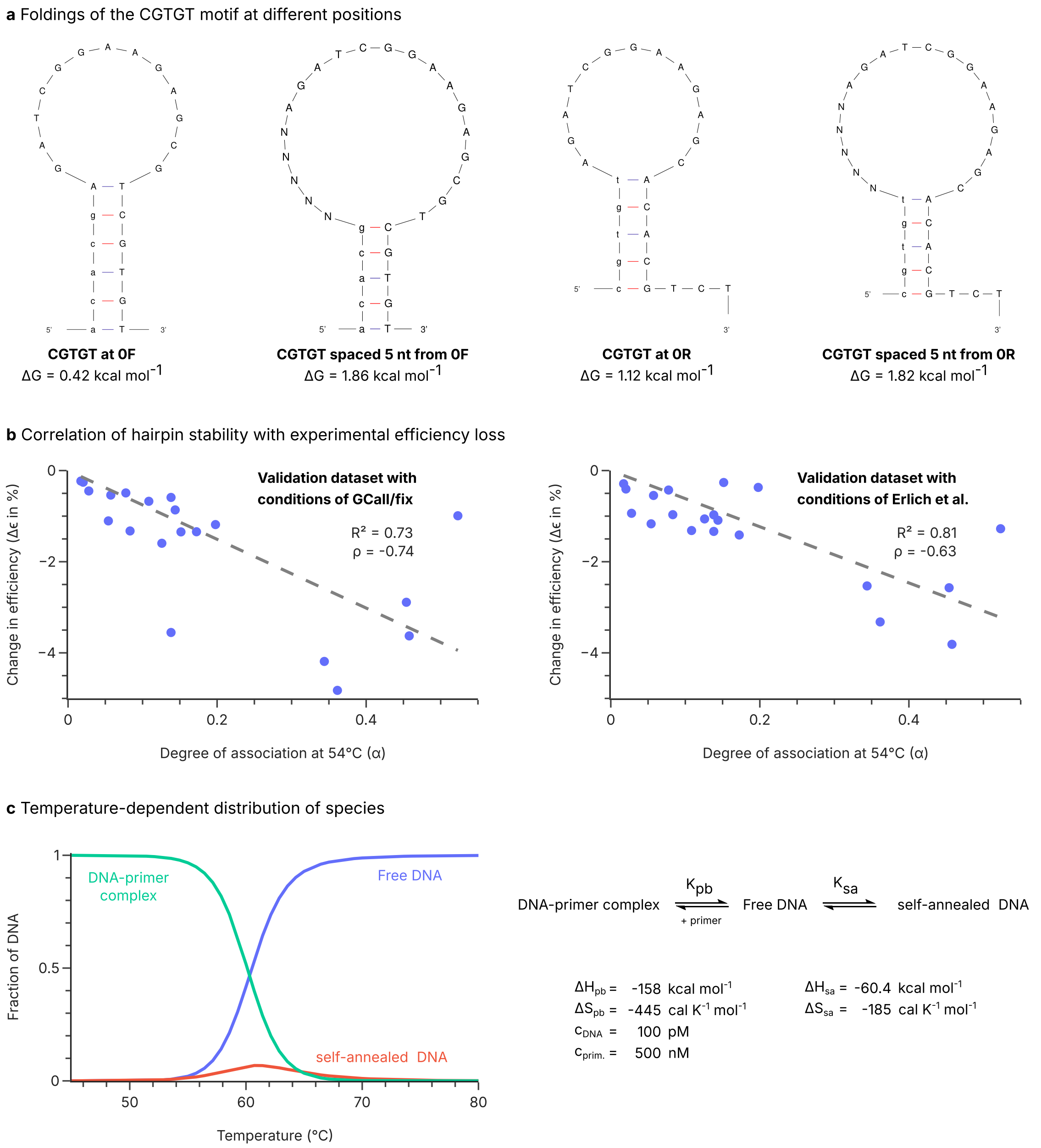

Supplementary Fig. 15 Thermodynamic considerations for template-motif interactions. (a) Predicted foldings of the CGTGT motif (lowercase) with the 5'- and 3'-adapters (0F and 0R, respectively). To illustrate the effect of additional spacing between the motif and the end of the adapter, interactions are also shown including a 5 N spacer. (b) Correlation between the degree of association calculated from the thermodynamic parameters, and the change in amplification efficiency observed in the experiment (see Fig. 6) for each motif at each position of a design sequence. The left plot uses the experimental data using the conditions of GCall/GCfix, whereas the plot on the right uses the experimental conditions of Erlich et al. (c) Thermodynamic equilibrium between the free DNA, the DNA-primer complex, and self-annealed DNA, illustrated as an example for one motif. The distribution of free DNA (blue), the DNA-primer complex (green) and the self-annealed DNA (red) is shown as a function of temperature, assuming temperature-independent thermodynamic parameters. All thermodynamic parameters were calculated at 54 °C and 50 mM Na^+ using the mfold webserver. Source data are provided as a Source Data file.

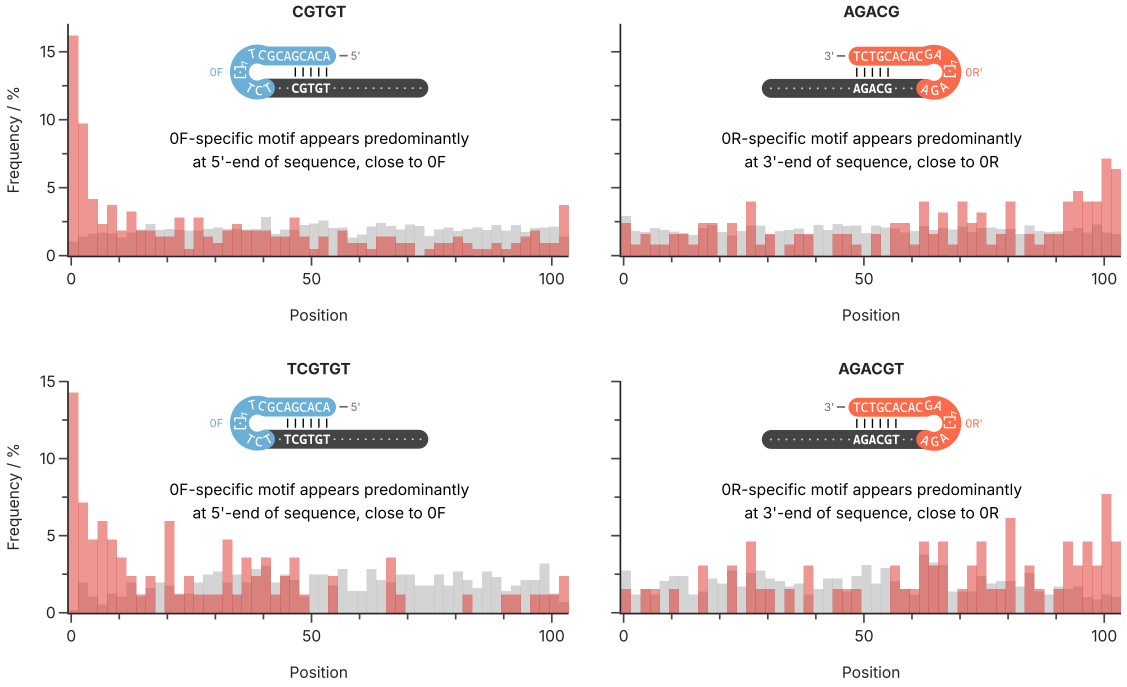

Supplementary Fig. 16 Positional distribution of individual k-mers in the datasets of GCall and GCfix, within poorly (red) and normally amplifying sequences (gray). Histograms show the frequency in 2 nt-windows among all positions in which a k-mer occurs. Insets show the hypothesized self-annealing that occurs at the sequences' 5'- and 3'-ends. Source data are provided as a Source Data file.

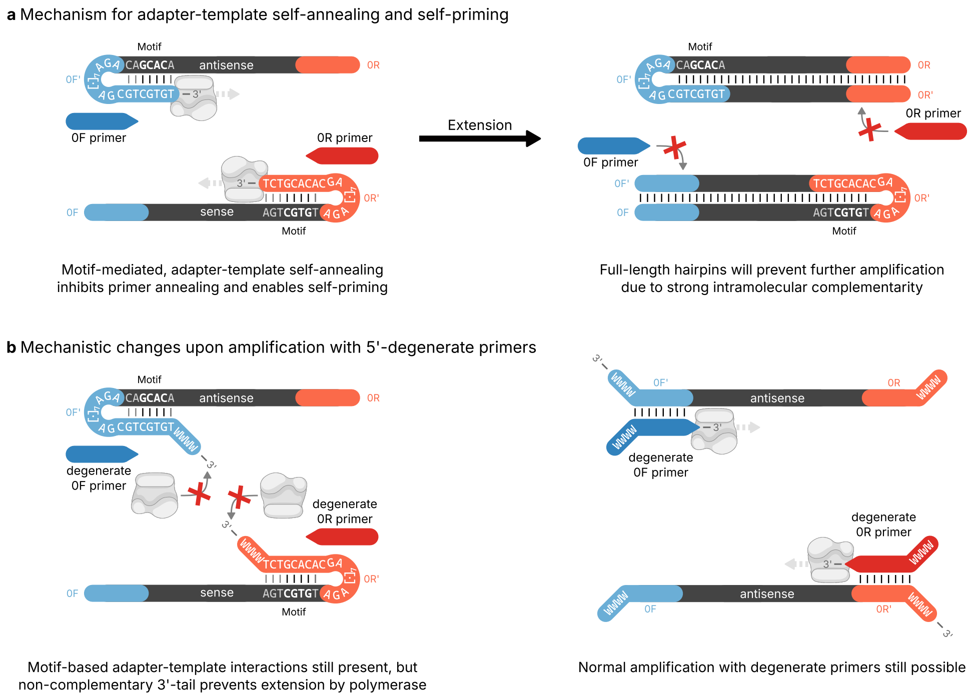

Supplementary Fig. 17 Illustration of the proposed mechanism for self-priming. (a) Motif-mediated interactions between the adapter at the front (top) or the end (bottom) of the design sequence and the template lead to hairpin formation. Due to the free 3'-end of the adapter involved in the hairpin, annealing of the primer is inhibited, and extension of the hairpin by a polymerase is facilitated. This leads to full-length hairpins that may no longer be amplified due to their self-complementarity (right). (b) Upon amplification with 5'-degenerate primers, the hairpin formation is still possible, however, the non-complementary 3'-ends introduced into the template during amplification prevent extension of hairpins by polymerases (left). Meanwhile, normal amplification is still possible (right). Partially created with BioRender. Gimpel, A. (2025) https://BioRender.com/19eu0ya.

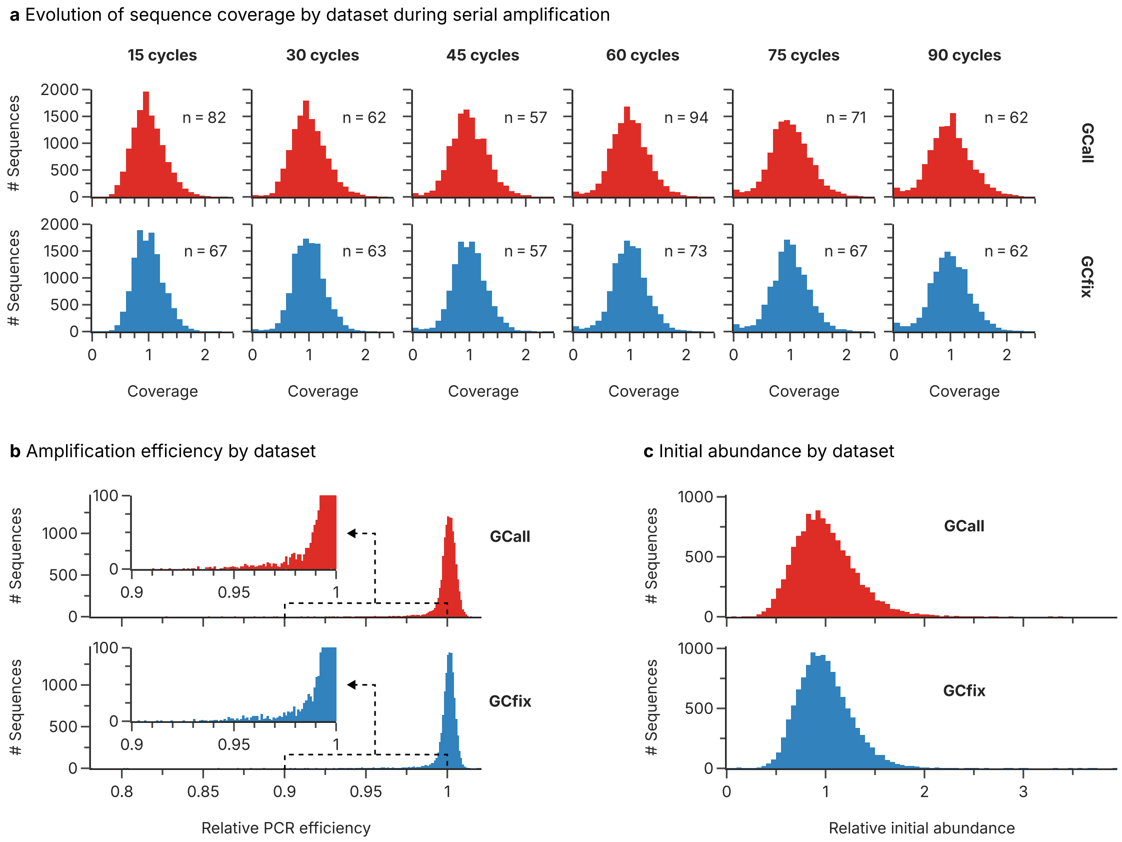

Supplementary Fig. 18 Comparison of the GCall and GCfix pools during serial amplification. The evolution of sequence coverage during serial amplification (a) for the GCall (top, red) and GCfix (bottom, blue) pools is shown, with the mean sequencing coverage given for each sequencing endpoint (n). The distributions of the estimated amplification efficiency (b) and the initial abundance (c) between the GCall (top, red) and GCfix (bottom, blue) datasets are also very similar. Source data are provided as a Source Data file.

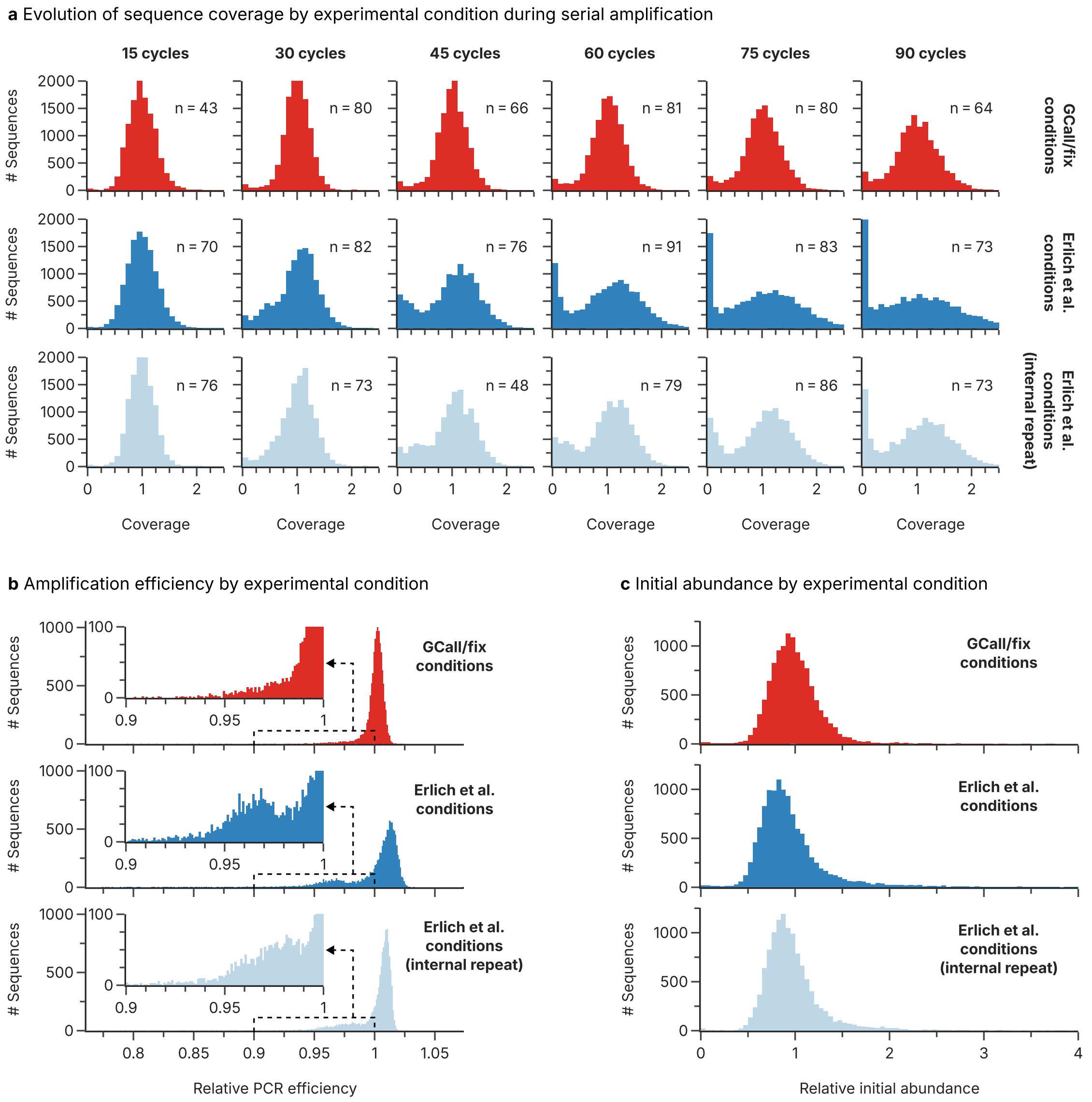

Supplementary Fig. 19 Comparison of the validation pools during serial amplification. The evolution of sequence coverage during serial amplification (a) for the validation pool using the GCall/GCfix conditions (top, red), the Erlich et al. conditions (middle, dark blue), and the internal repeat of the Erlich et al. conditions (bottom, light blue) is shown, with the mean sequencing coverage given for each sequencing endpoint (n). The distributions of the estimated amplification efficiency (b) and the initial abundance (c) between the GCall/GCfix conditions (top, red), the Erlich et al. conditions (middle, dark blue), and the internal repeat of the Erlich et al. conditions (bottom, light blue) are dissimilar only for the GCall/GCfix conditions. Source data are provided as a Source Data file.

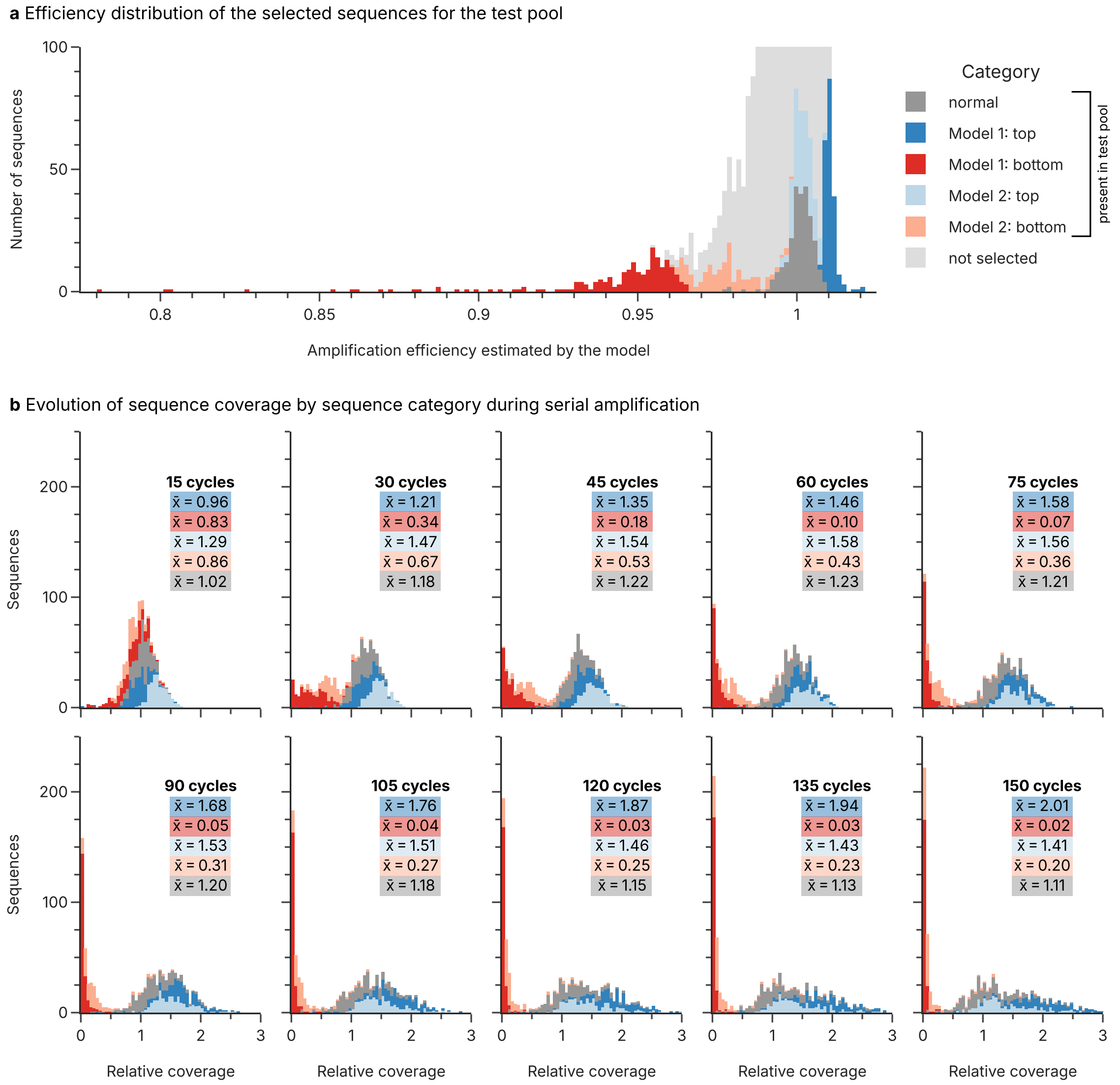

Supplementary Fig. 20 Composition and serial amplification of the test pool. The test pool for validation of estimated amplification efficiencies was composed of selected sequences from both the GCall and GCfix pools. Selection of 1,000 sequences from all 24,000 sequences was performed based on the estimations of amplification efficiency by two models (a), see also Supplementary Note 3. The simpler model 1 considered only exponential amplification, whereas model 2 also considered dilutions and sequencing propensity. The evolution of the coverage of the selected sequences after synthesis and serial amplification (b) highlights that both models identify poorly amplifying sequences. In both figures, the sequences selected from model 1 are shown in dark colors (dark red: poorly amplifying, dark blue: well amplifying), whereas the selected sequences from model 2 are shown in light colors (light red: poorly amplifying, light blue: well amplifying). The randomly selected sequences (gray, labeled normal) reflect the evolution of an average sequence during serial amplification. Source data are provided as a Source Data file.

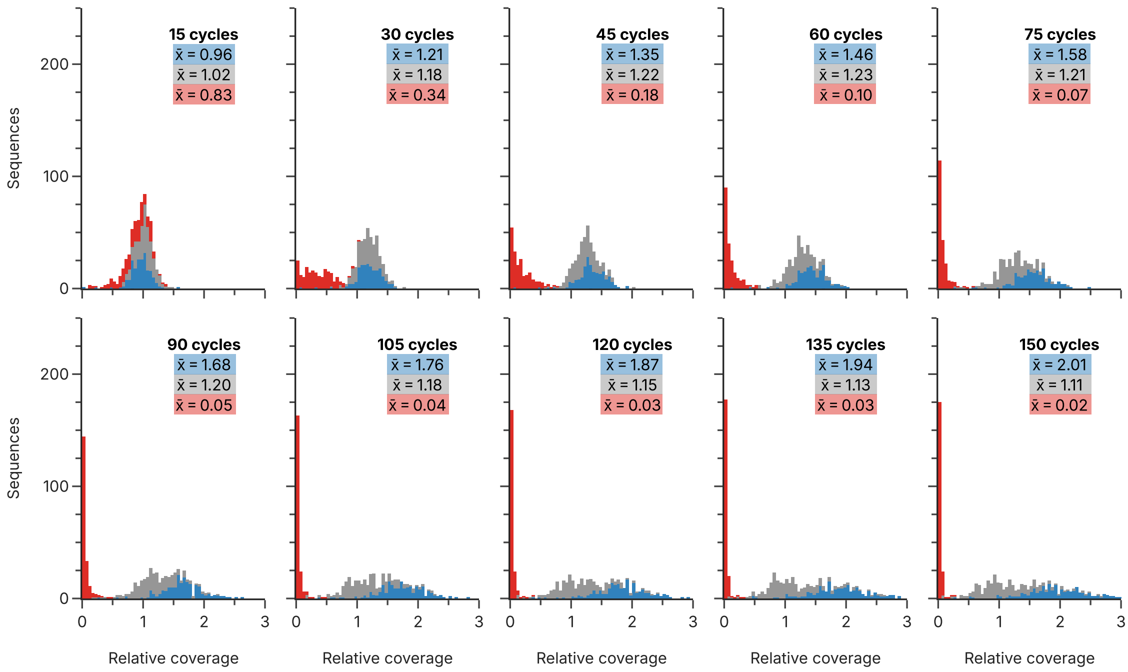

Supplementary Fig. 21 Composition and serial amplification of the test pool. This figure is a variant of Supplementary Fig. 20b showing only the evolution of the sequences selected by model 1. The sequences identified by the model as poorly amplifying (red), or well amplifying (blue) are shown together with randomly selected sequences (gray) from the GCall and GCfix pools during serial amplification. Source data are provided as a Source Data file.

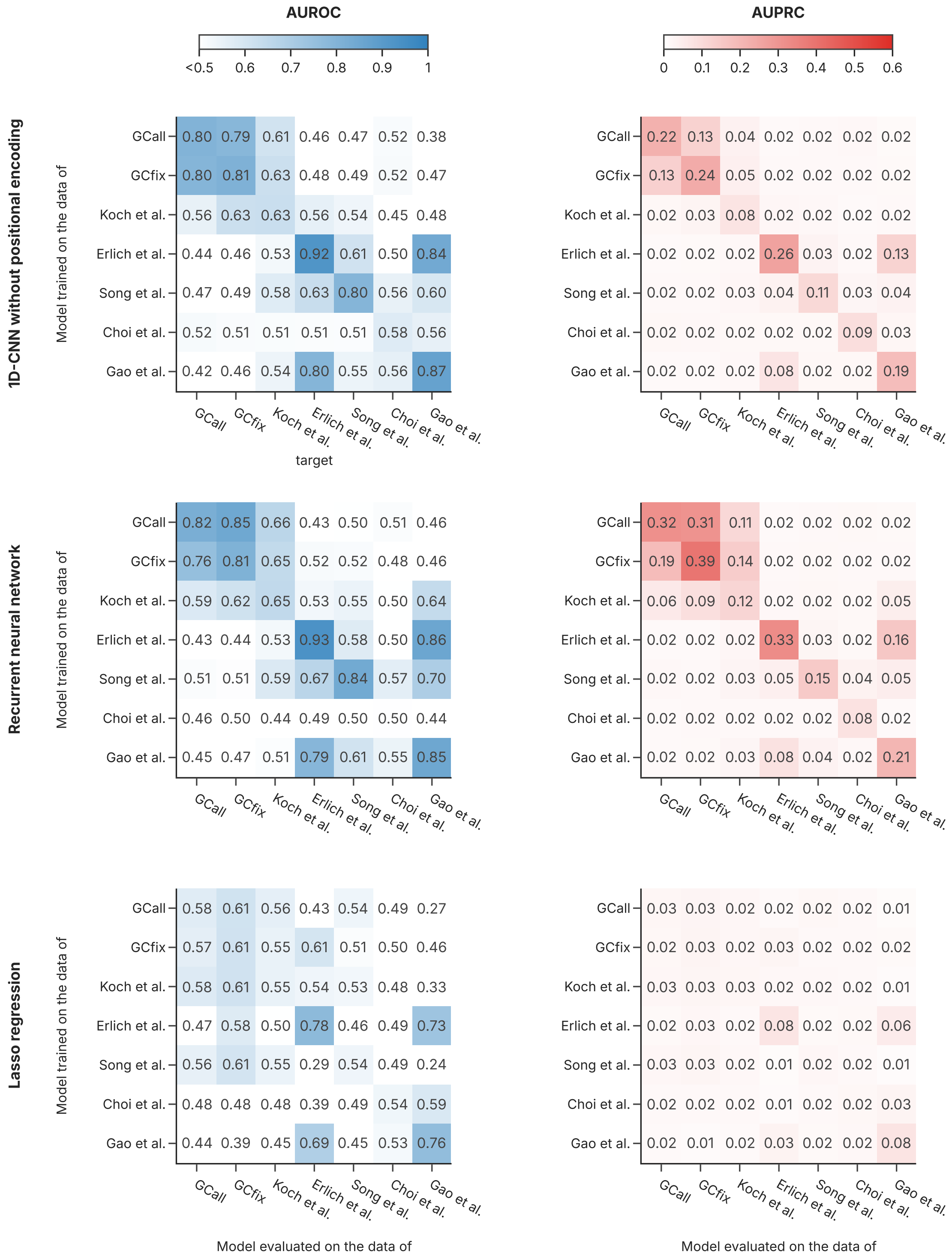

Supplementary Fig. 22 This figure presents the AUROC and AUPRC for different baseline models, including 1D-CNN with and without positional encoding, recurrent neural network, and Lasso regularized logistic regerssion, trained and evaluated across various literature datasets. The models are evaluated on their ability to categorize DNA sequences into low-efficiency and normal-efficiency classes based on the empirically set 2% threshold.

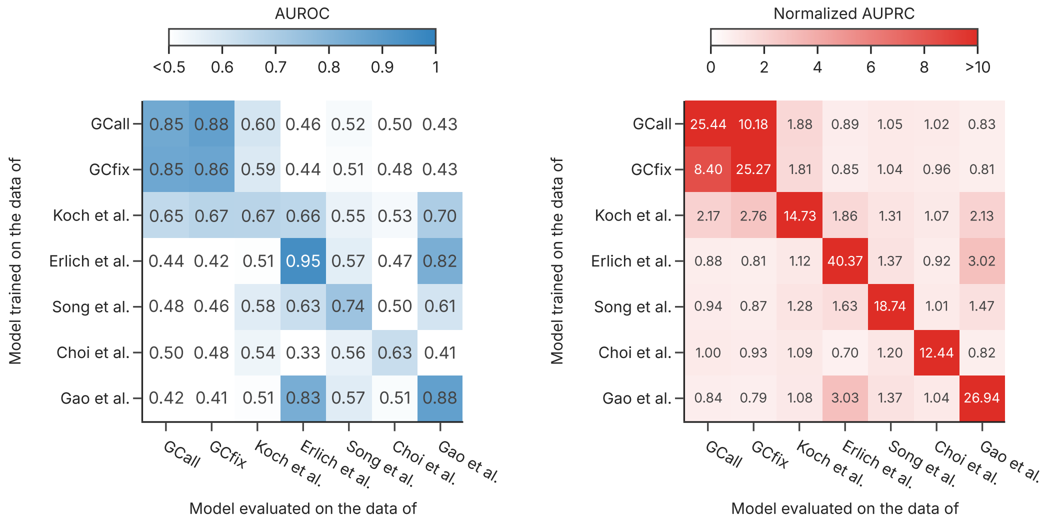

Supplementary Fig. 23 This figure presents the AUROC and the normalized AUPRC (AUPRC over the positive class prevalence) for the proposed 1D-CNN + PE model trained and evaluated across various literature datasets. The models are evaluated on their ability to categorize DNA sequences into low-efficiency and normal-efficiency classes based on a threshold defined as 1 standard deviation below the mean PCR efficiency.

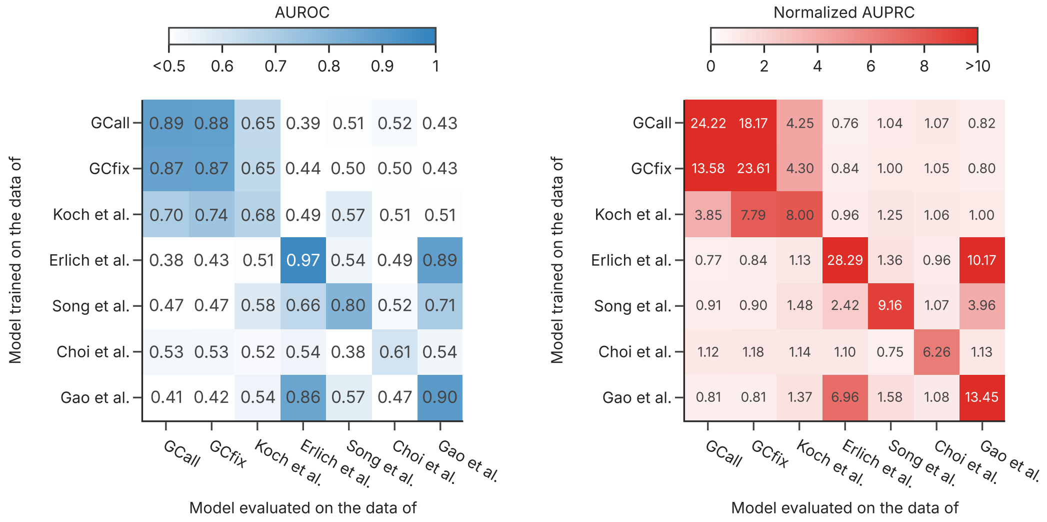

Supplementary Fig. 24 This figure presents the AUROC and the normalized AUPRC (AUPRC over the positive class prevalence) for the proposed 1D-CNN + PE model trained and evaluated across various literature datasets. The models are evaluated on their ability to categorize DNA sequences into low-efficiency and normal-efficiency classes based on a threshold defined as 2 standard deviations below the mean PCR efficiency.

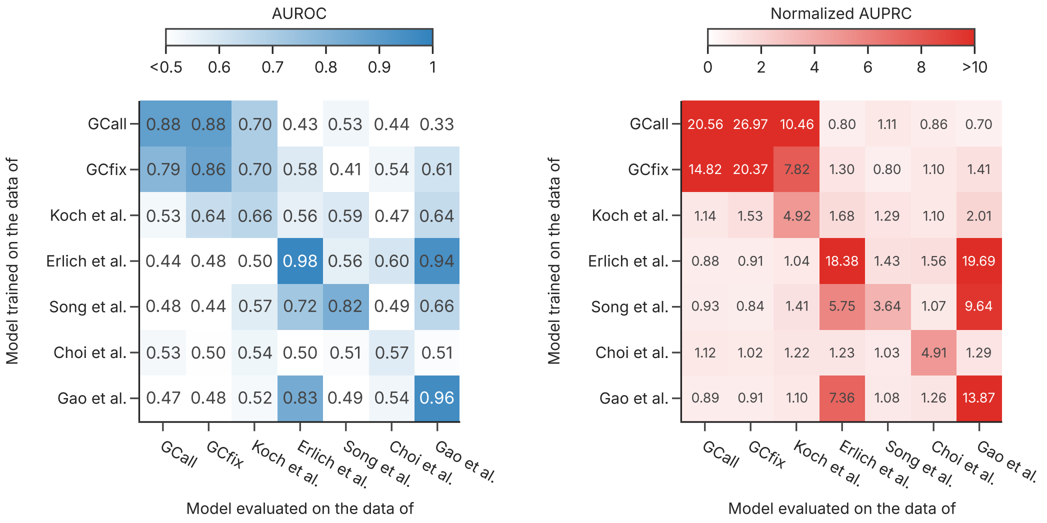

Supplementary Fig. 25 This figure presents the AUROC and the normalized AUPRC (AUPRC over the positive class prevalence) for the proposed 1D-CNN + PE model trained and evaluated across various literature datasets. The models are evaluated on their ability to categorize DNA sequences into low-efficiency and normal-efficiency classes based on a threshold defined as 3 standard deviations below the mean PCR efficiency.

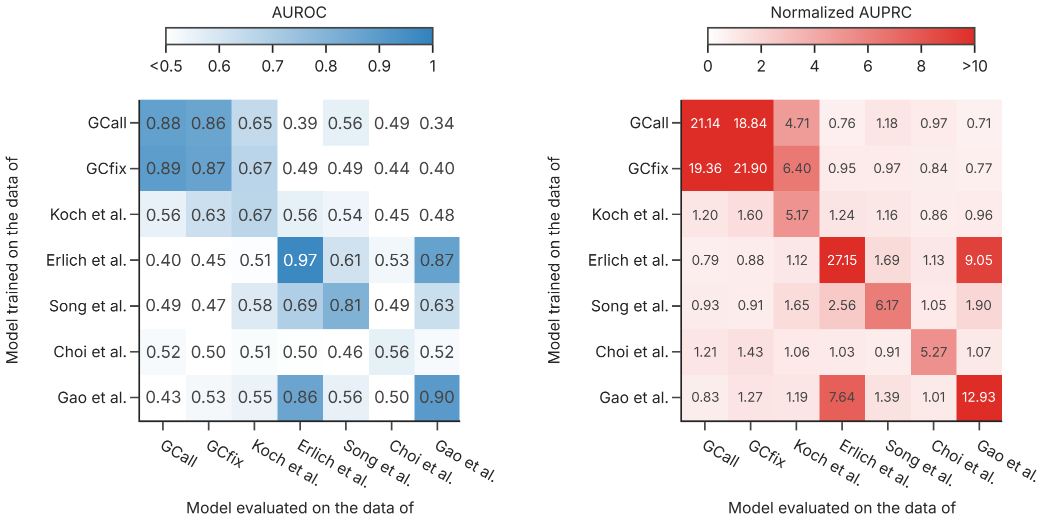

Supplementary Fig. 26 This figure presents the AUROC and the normalized AUPRC (AUPRC over the positive class prevalence) for the proposed 1D-CNN + PE model trained and evaluated across various literature datasets. The models are evaluated on their ability to categorize DNA sequences into low-efficiency and normal-efficiency classes based on a threshold defined as 2% of the total sequences.

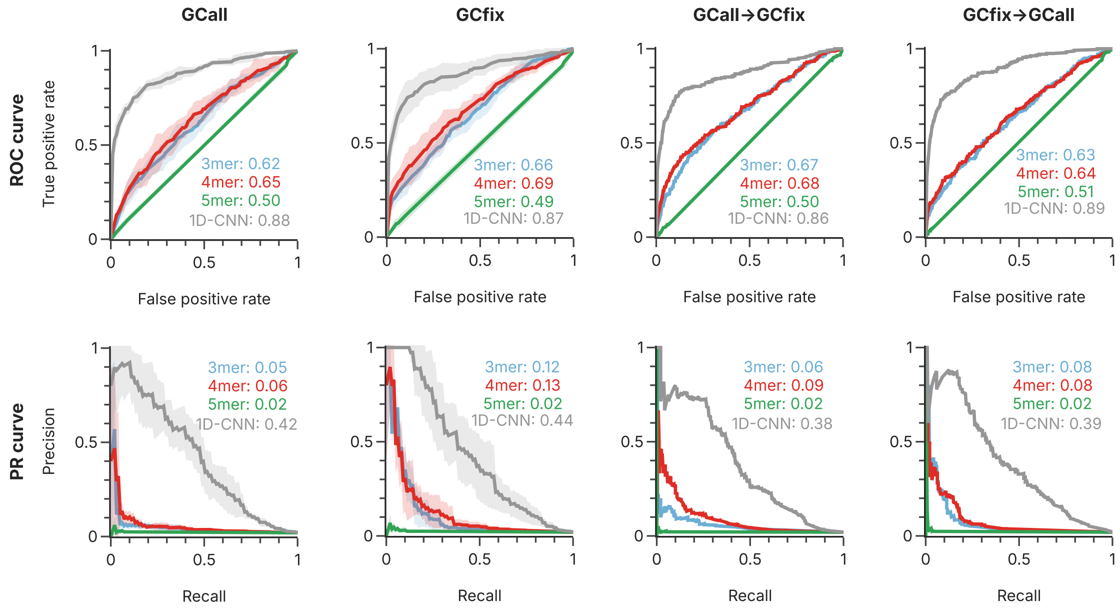

Supplementary Fig. 27 Performance comparison between k-mer based models and 1D-CNN model on the internal and external validation of GCfix and GCall datasets. Lines show the mean performance, with the shaded area showing the standard deviation from the five-fold cross-validation. Numbers in the plots indicate the area under the curve for the respective model. Source data are provided as a Source Data file.

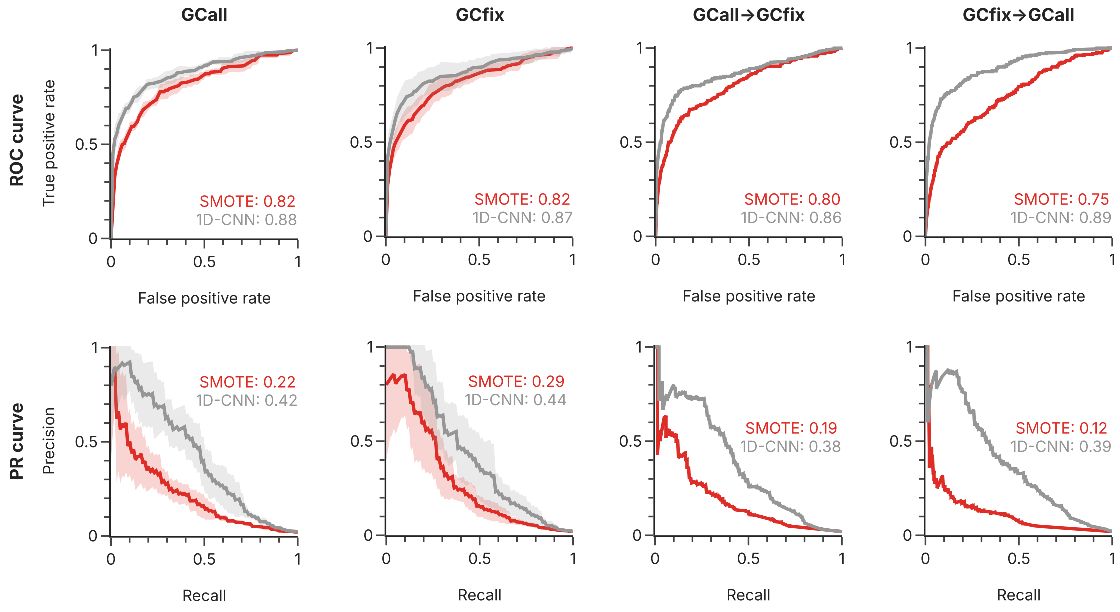

Supplementary Fig. 28 Performance comparison between 1D-CNN model with and without over sampling of the minority class (SMOTE) technique on the internal and external validation of GCfix and GCall datasets. Lines show the mean performance, with the shaded area showing the standard deviation from the five-fold cross-validation. Numbers in the plots indicate the area under the curve for the respective model. Source data are provided as a Source Data file.

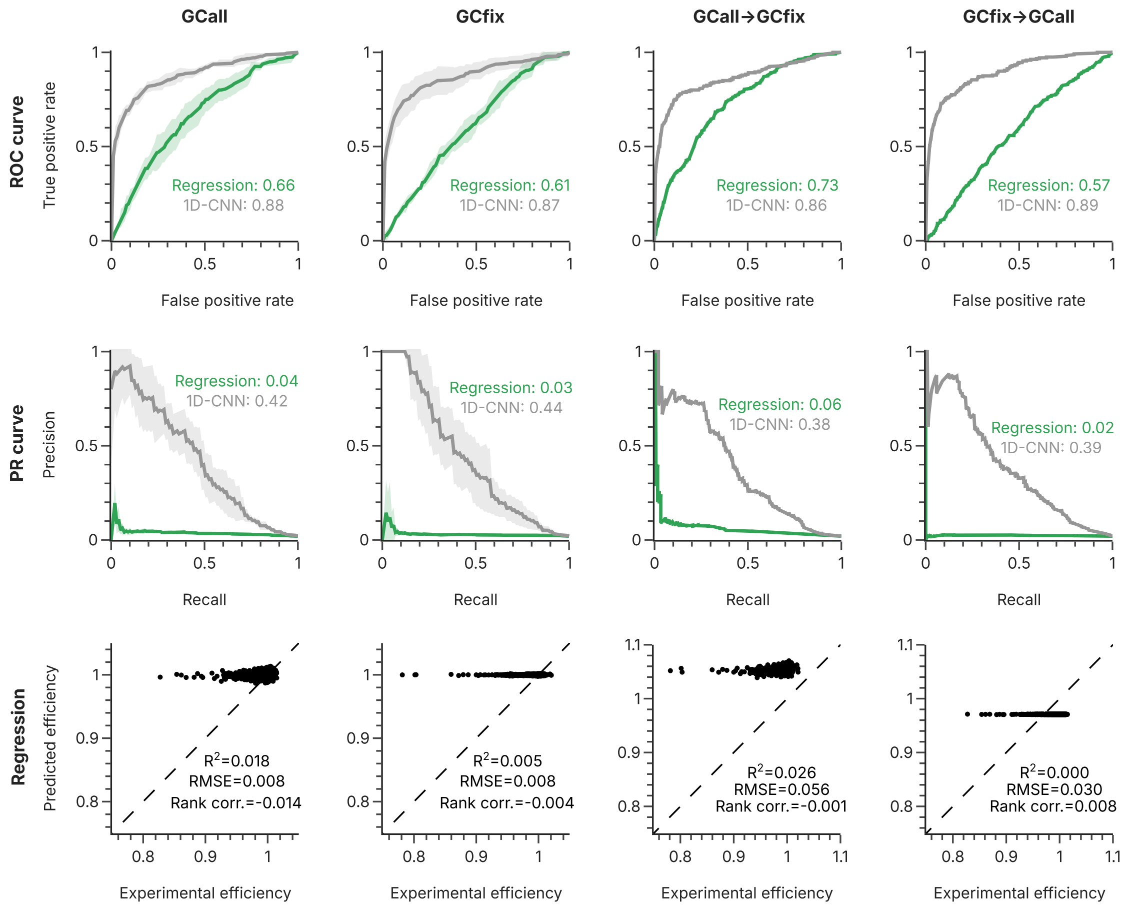

Supplementary Fig. 29 Performance comparison between regression-driven model and 1D-CNN model on the internal and external validation of GCfix and GCall datasets. The third row shows the direct regression performance, in which the R^2 score, RMSE and rank correlation are presented. Lines show the mean performance, with the shaded area showing the standard deviation from the five-fold cross-validation. Numbers in the plots indicate the area under the curve for the respective model. Source data are provided as a Source Data file.

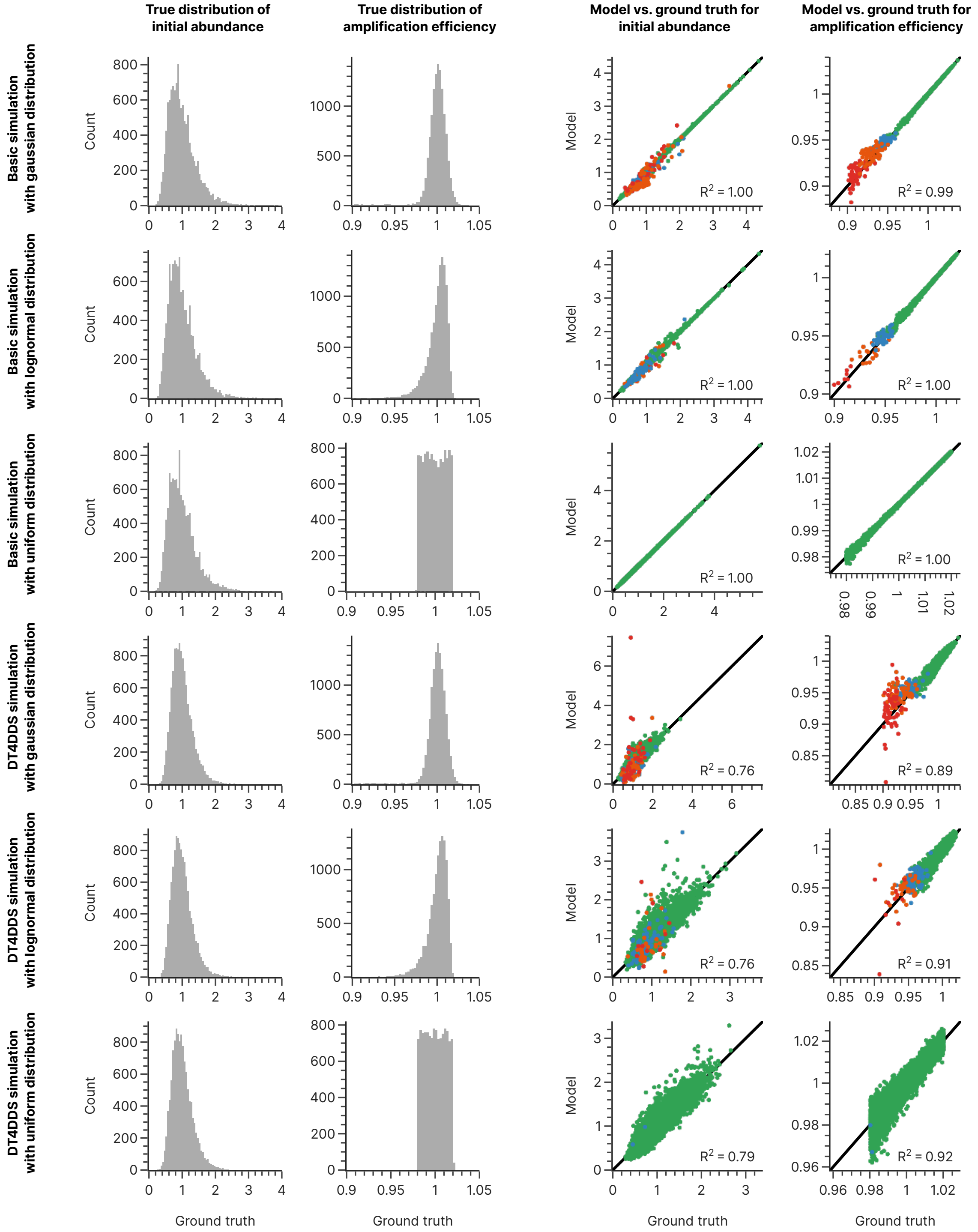

Supplementary Fig. 30 Verification of the two-parameter fitting of the exponential PCR equation using in-silico simulations. Sequencing data with varying distributions of the true amplification efficiency and the true initial coverage bias were generated using a basic model and the DT4DDS software (left column, see Methods). The results of the parameter estimation using the exponential PCR equation were compared to the ground truth parameters used for simulation (right column), and show good agreement across all tested parameter distributions. The simulated datasets also consisted of six experimental endpoints at different cycle counts, identical to the experimental datasets. The color of the points in the right column corresponds to the frequency with which the sequence was not observed in the six experimental endpoints: never (i.e., always present, green), once (blue), twice (orange), and at least three times (red). Source data are provided as a Source Data file.

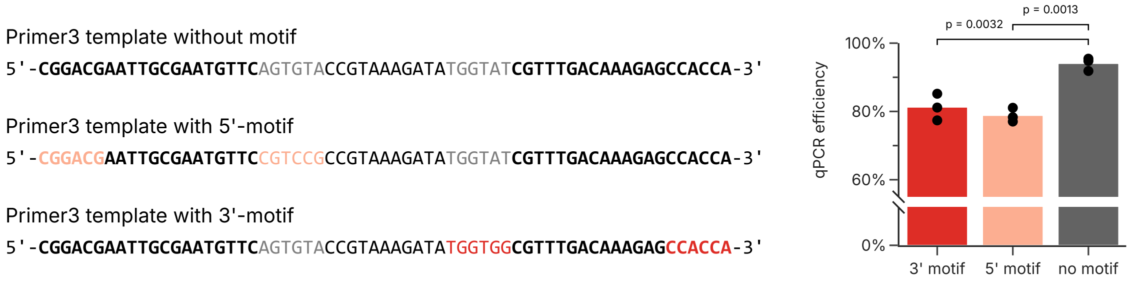

Supplementary Fig. 31 qPCR experiments demonstrating the motif-dependent inhibitory effect using a different primer set. Using Primer3Plus,[8] a primer set and amplicon (bold) were picked from a random 1000 bp sequence. The corresponding amplicon (Primer3 template without motif, top), as well as amplicons with primer-appropriate motifs at the 5'- (Primer3 template with 5'-motif, middle) and 3'-end (Primer3 template with 3'-motif, bottom) were ordered from Microsynth AG (Balgach, Switzerland). Based on qPCR dilution curves, the amplification efficiency of each amplicon was determined in three experimental replicates. Black dots denote the individual data points. Differences between amplicons' amplification efficiencies were assessed after a one-way ANOVA ($N = 3$ per sample, $F(2, 6) = 26.1$, $p = 0.0011$) using pairwise comparisons with Tukey's range test. Tukey's range test accounts for the family-wise error rate during multiple comparisons. Source data are provided as a Source Data file.

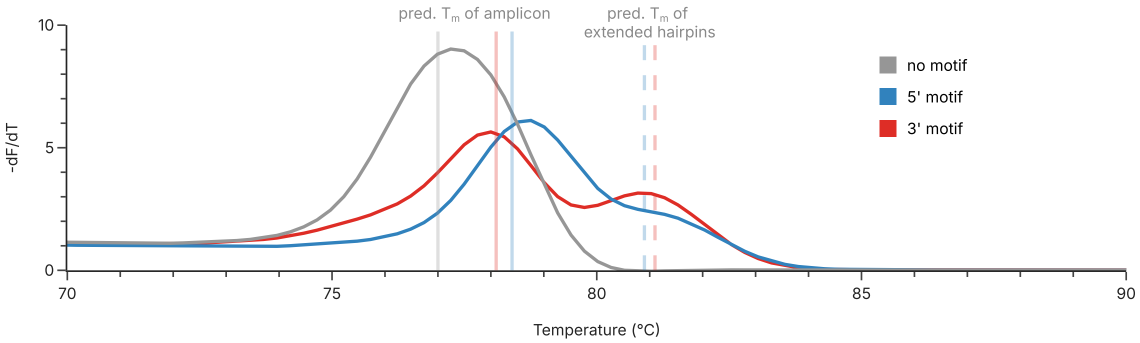

Supplementary Fig. 32 Melting curve analysis of the templates picked with Primer3, after normal amplification with primers for 15 cycles. The template without motif (grey) exhibits a singular melting peak, whereas the templates with 5'-motif (blue) and 3'-motif (red) exhibit two melting peaks. Also shown are the predicted melting temperatures of the amplicons (solid lines, via OligoAnalyzer by IDT, eu.idtdna.com/calc/analyzer) and the extended hairpins (dashed lines, via mfold, www.unafold.org/mfold/applications/dna-folding-form.php). Source data are provided as a Source Data file.

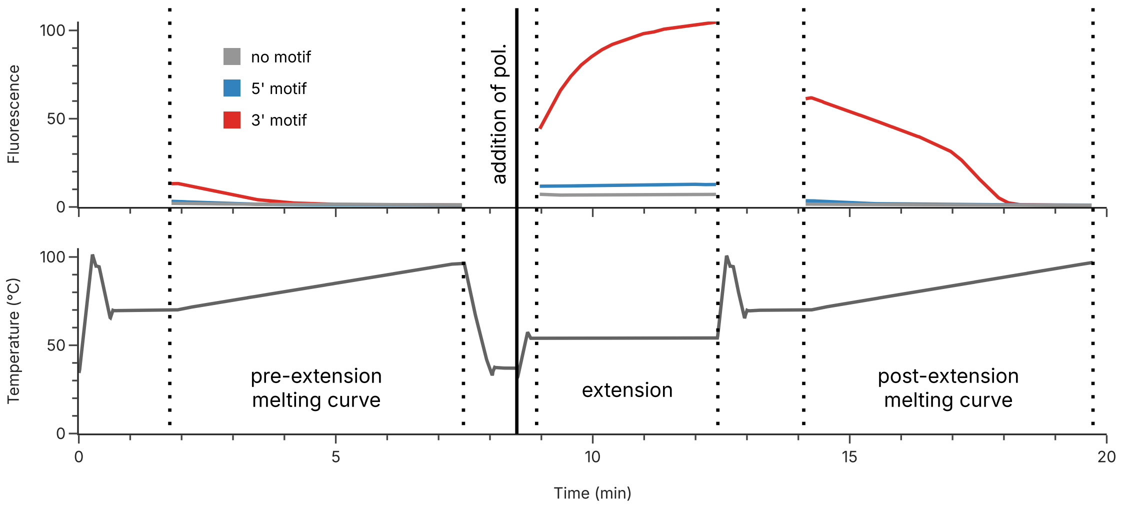

Supplementary Fig. 33 Fluorescence (top) and temperature profiles (bottom) throughout the primer-less extension of the templates picked with Primer3. The template without motif (grey) and with a 5'-motif (blue) did not exhibit a melting transition in both the pre- and post-extension melting curves, and did not increase in fluorescence during the extension phase. In contrast, the template with 3'-motif exhibited a melting transition in the post-extension melting curve and increased in fluorescence throughout the extension phase. Dashed vertical lines denote the boundaries of different measurement phases, and the solid vertical line denotes the addition of a polymerase to each sample. The melting profiles of the pre- and post-extension melting curves are shown separately in Supplementary Fig. 34. Source data are provided as a Source Data file.

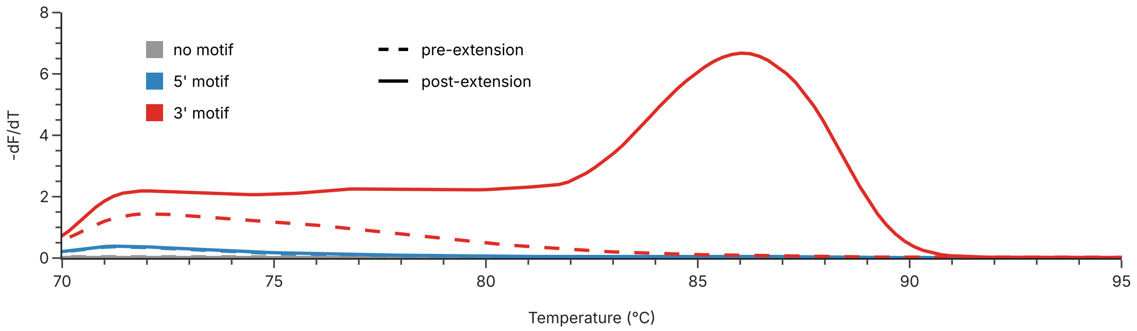

Supplementary Fig. 34 Melting curve analysis of the templates picked with Primer3, before (dashed) and after (solid) extension without primers. The templates without motif (grey) and with 5'-motif (blue) do not exhibit any melting peak both prior and after the extension phase. The template with 3'-motif does not show a melting transition before the extension, but has a distinct melting peak after the extension. Note that this melting curve is not directly comparable to the melting curve shown in Supplementary Fig. 32, as the composition of both master mixes differs (e.g., Mg^{2+} concentration). Source data are provided as a Source Data file.

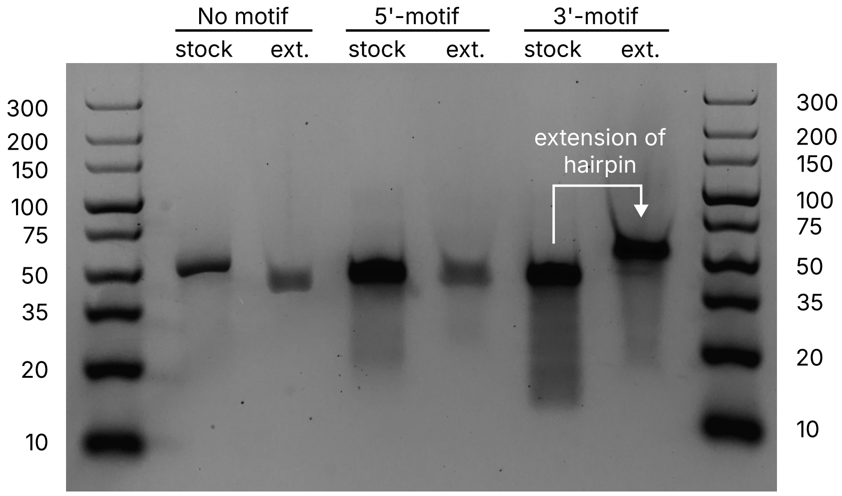

Supplementary Fig. 35 Agarose gel electrophoresis (4% with SYBR Gold II, E-Gel EX Agarose Gels, Invitrogen) of the templates picked with Primer3, before ("stock") and after extension ("ext.") without primers. Note that the intensity of samples after extension is lower for the sequence without motif and with the 5'-motif, due to the dilution during extension. In contrast, the sequence with 3'-motif exhibits a higher intensity after extension, due to its double-strandedness after extension. A reference ladder (Ultra Low Range DNA Ladder, Invitrogen) was run in the first and last well (annotated by base pairs on each side). Gel was illuminated on a transilluminator with filter (E-Gel Power Snap Electrophoresis System, Invitrogen) and photographed with a digital camera (Sony RX10II, ZEISS Vario-Sonnar T* F2.8). Final image was cropped, rotated, converted to grayscale, and inverted with GIMP 3.

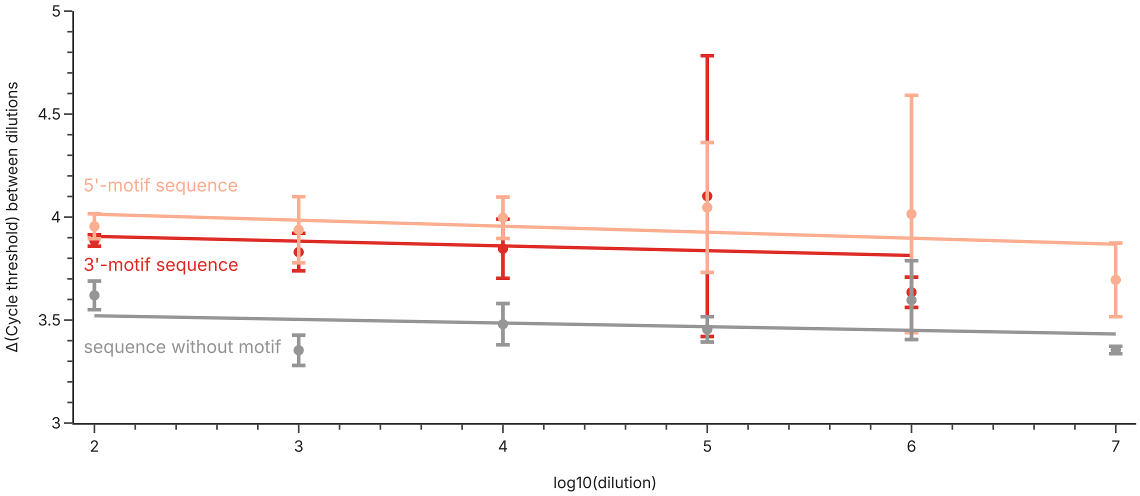

Supplementary Fig. 36 Difference in cycle thresholds between subsequent dilutions of the three individually synthesized template sequences Primer3 without motif (gray), Primer3 with 5'-motif (light red), and Primer3 with 3'-motif (dark red). Shown are the mean of the three individual experimental runs, with brackets indicating the standard deviation. The solid line represents a linear regression, indicating only a minor decrease as a function of dilution. Source data are provided as a Source Data file.

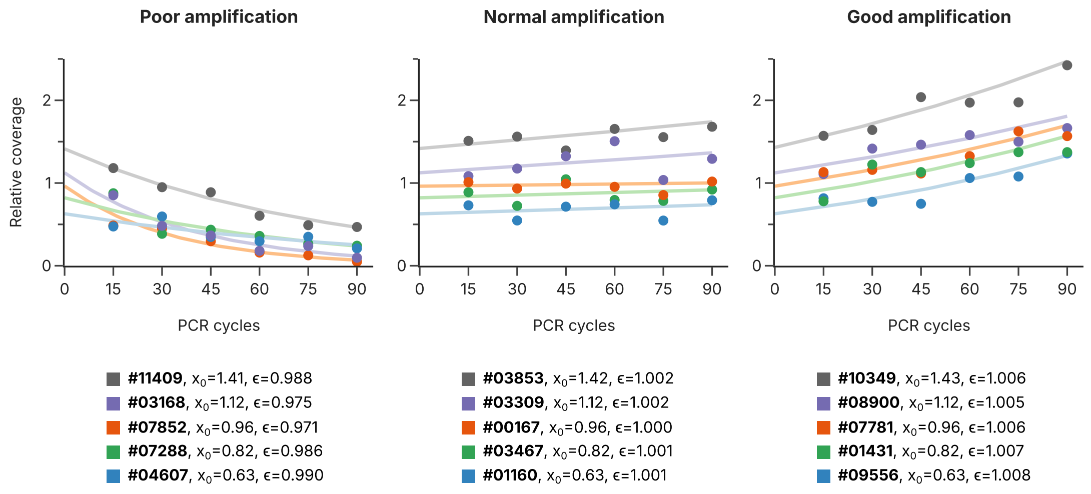

Supplementary Fig. 37 Exemplary trajectories of the relative coverage of different sequences from the GCall dataset. In each group of poor (left), normal (middle), and good amplification (right), the relative coverage as a function of the number of PCR cycles is shown for five exemplary sequences. Points denote the coverage in the experimental sequencing data, solid lines represent the model fit for estimating initial abundance and amplification efficiency. Source data are provided as a Source Data file.

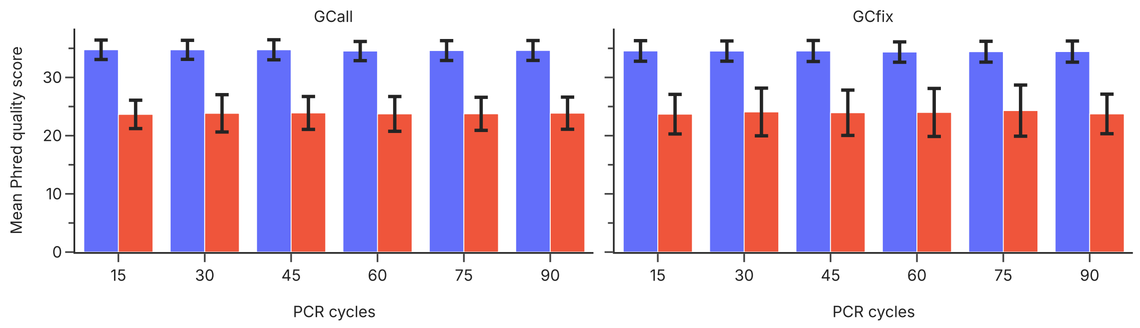

Supplementary Fig. 38 Mean Phred quality scores of the mapped (blue) and unmapped (red) sequencing reads of the sequencing data for the GCall (left) and GCfix (right) datasets. Shown are the mean quality scores per read, with the standard deviation shown as brackets. Source data are provided as a Source Data file.

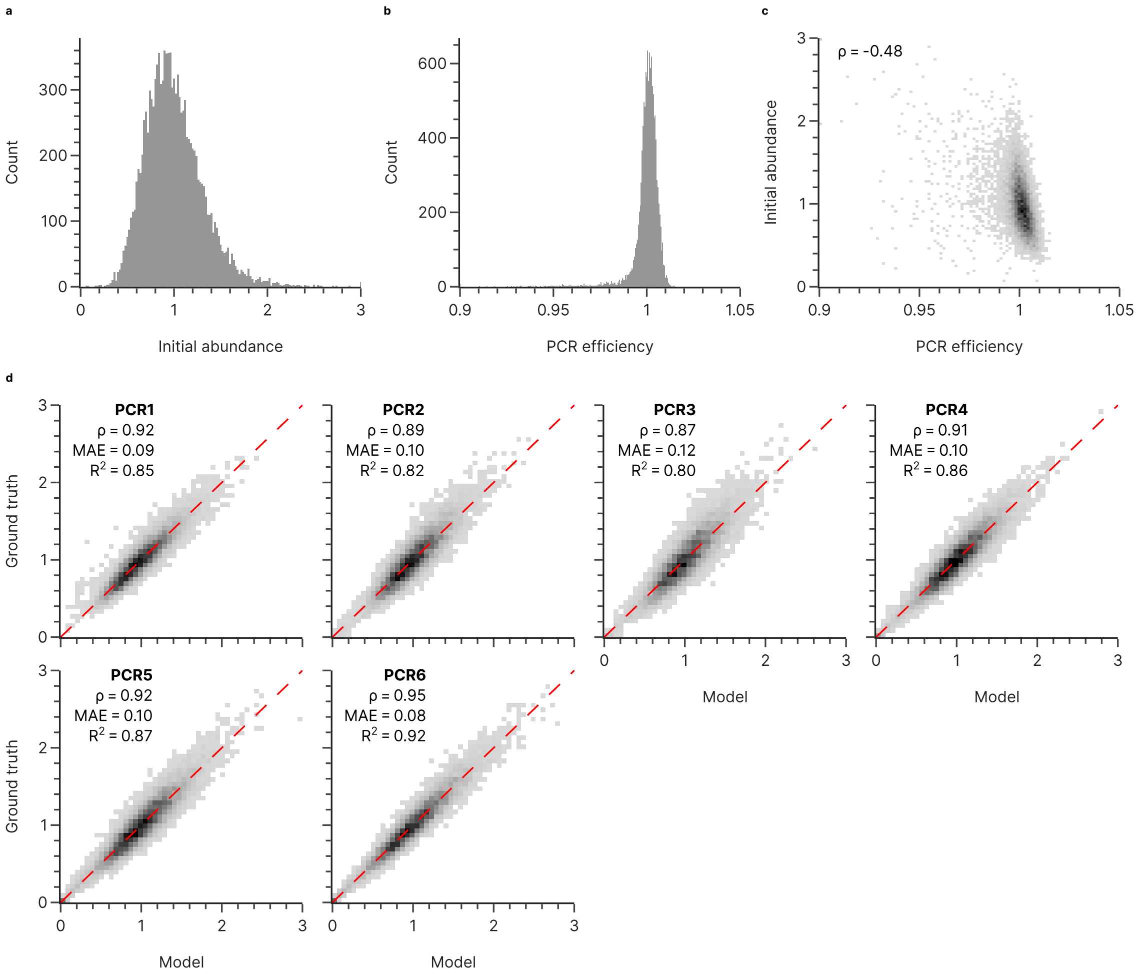

Supplementary Fig. 39 Results and performance of the two-parameter fitting for the GCall dataset. The distributions of the fitted parameters of the initial abundance (a) and the amplification efficiency (b) are shown, as well as the correlation between both parameters (c). For each sequencing endpoint, the plots in (d) show the agreement between the experimental abundance (Ground truth, y-axis) and the expected abundance based on the PCR model and the fitted parameters (Model, x-axis). Panels (c) and (d) show relative density, and (d) includes the diagonal of perfect agreement (red, dashed line), as well as corresponding metrics (Spearman's rank correlation coefficient ρ, mean absolute error MAE, and coefficient of determination R^2). In panels (a-c), the values exceeding the axis have been clipped to the axis limits for visual clarity. Source data are provided as a Source Data file.

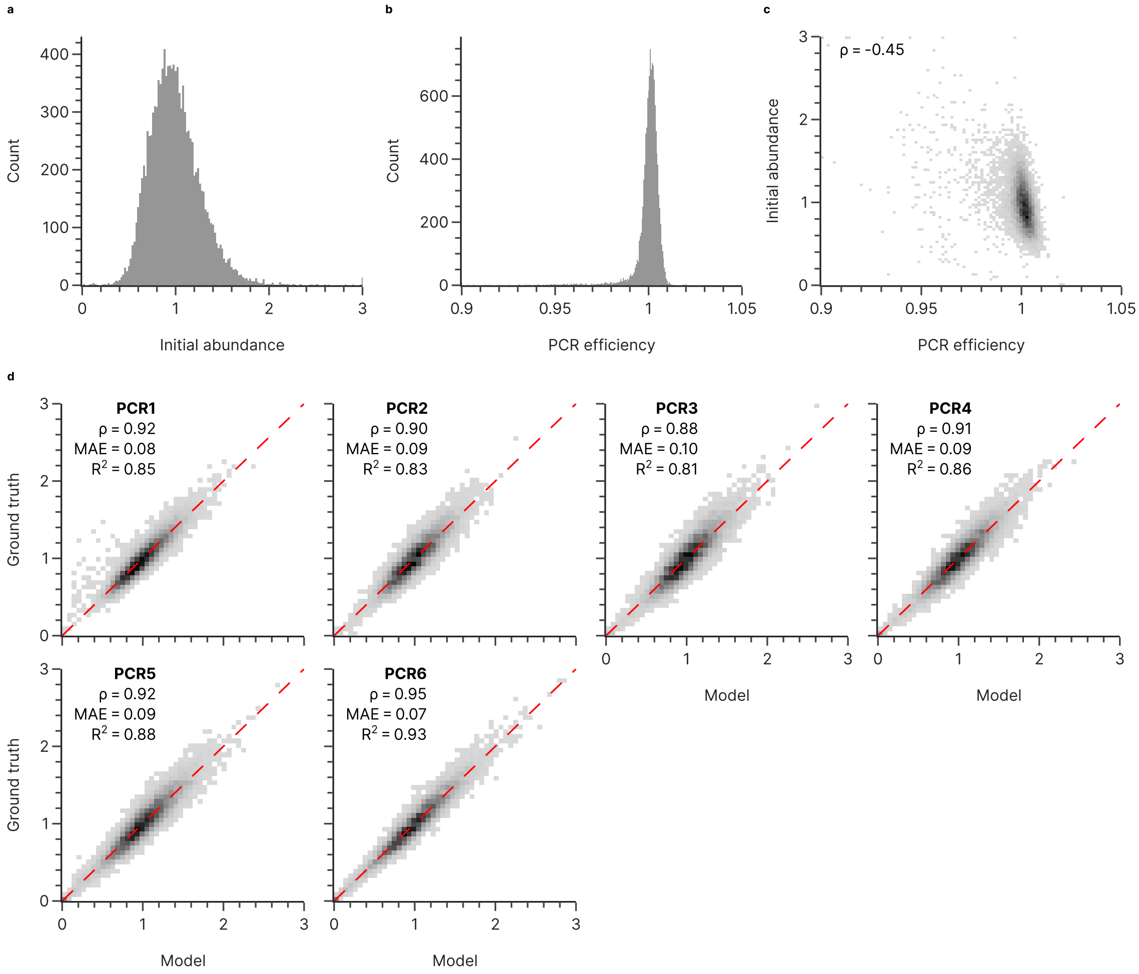

Supplementary Fig. 40 Results and performance of the two-parameter fitting for the GCfix dataset. The distributions of the fitted parameters of the initial abundance (a) and the amplification efficiency (b) are shown, as well as the correlation between both parameters (c). For each sequencing endpoint, the plots in (d) show the agreement between the experimental abundance (Ground truth, y-axis) and the expected abundance based on the PCR model and the fitted parameters (Model, x-axis). Panels (c) and (d) show relative density, and (d) includes the diagonal of perfect agreement (red, dashed line), as well as corresponding metrics (Spearman's rank correlation coefficient ρ, mean absolute error MAE, and coefficient of determination R^2). In panels (a-c), the values exceeding the axis have been clipped to the axis limits for visual clarity. Source data are provided as a Source Data file.

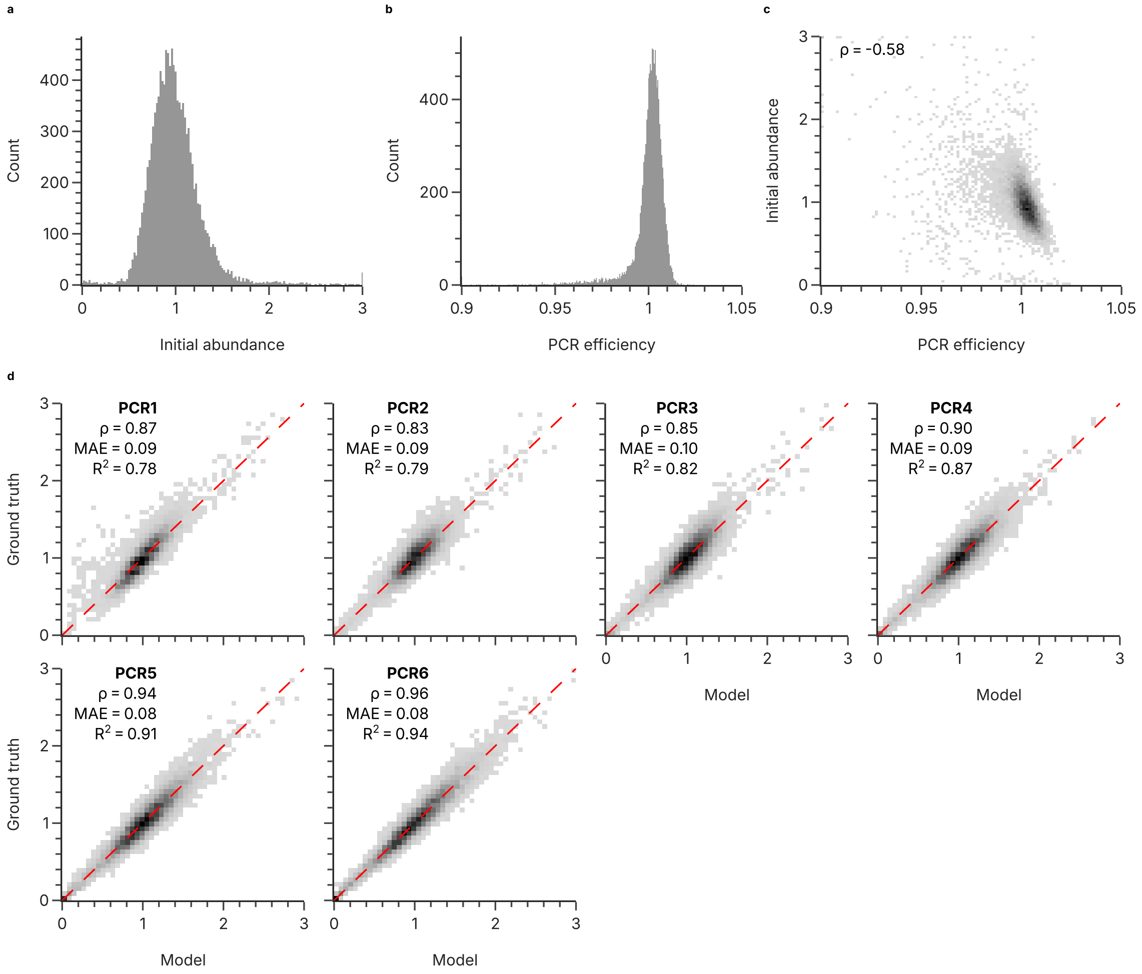

Supplementary Fig. 41 Results and performance of the two-parameter fitting for the validation dataset using the conditions of GCall/GCfix. The distributions of the fitted parameters of the initial abundance (a) and the amplification efficiency (b) are shown, as well as the correlation between both parameters (c). For each sequencing endpoint, the plots in (d) show the agreement between the experimental abundance (Ground truth, y-axis) and the expected abundance based on the PCR model and the fitted parameters (Model, x-axis). Panels (c) and (d) show relative density, and (d) includes the diagonal of perfect agreement (red, dashed line), as well as corresponding metrics (Spearman's rank correlation coefficient ρ, mean absolute error MAE, and coefficient of determination R^2). In panels (a-c), the values exceeding the axis have been clipped to the axis limits for visual clarity. Source data are provided as a Source Data file.

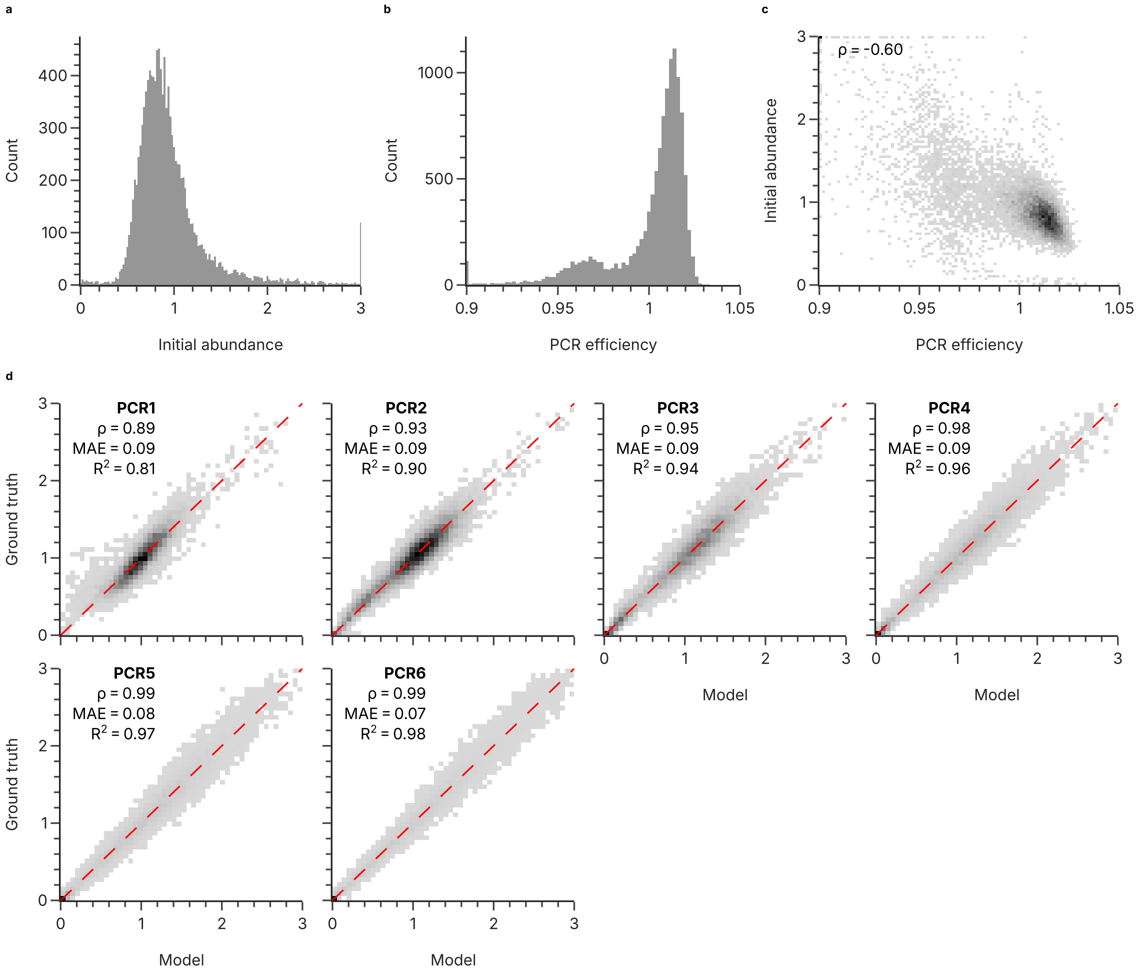

Supplementary Fig. 42 Results and performance of the two-parameter fitting for the validation dataset using the conditions of Erlich et al. The distributions of the fitted parameters of the initial abundance (a) and the amplification efficiency (b) are shown, as well as the correlation between both parameters (c). For each sequencing endpoint, the plots in (d) show the agreement between the experimental abundance (Ground truth, y-axis) and the expected abundance based on the PCR model and the fitted parameters (Model, x-axis). Panels (c) and (d) show relative density, and (d) includes the diagonal of perfect agreement (red, dashed line), as well as corresponding metrics (Spearman's rank correlation coefficient ρ, mean absolute error MAE, and coefficient of determination R^2). In panels (a-c), the values exceeding the axis have been clipped to the axis limits for visual clarity. Source data are provided as a Source Data file.

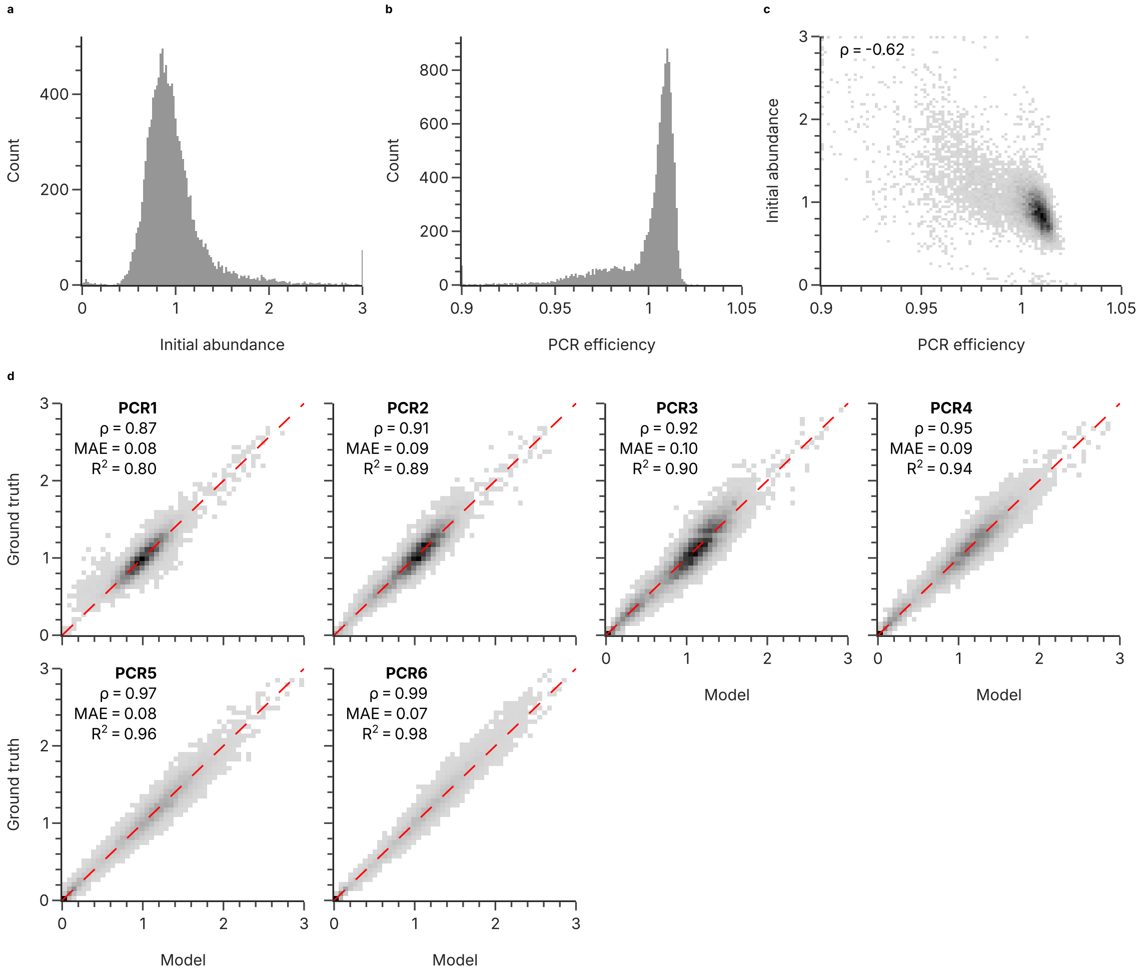

Supplementary Fig. 43 Results and performance of the two-parameter fitting for the validation dataset using the conditions of Erlich et al., during an internal repeat of the experiment. The distributions of the fitted parameters of the initial abundance (a) and the amplification efficiency (b) are shown, as well as the correlation between both parameters (c). For each sequencing endpoint, the plots in (d) show the agreement between the experimental abundance (Ground truth, y-axis) and the expected abundance based on the PCR model and the fitted parameters (Model, x-axis). Panels (c) and (d) show relative density, and (d) includes the diagonal of perfect agreement (red, dashed line), as well as corresponding metrics (Spearman's rank correlation coefficient ρ, mean absolute error MAE, and coefficient of determination R^2). In panels (a-c), the values exceeding the axis have been clipped to the axis limits for visual clarity. Source data are provided as a Source Data file.

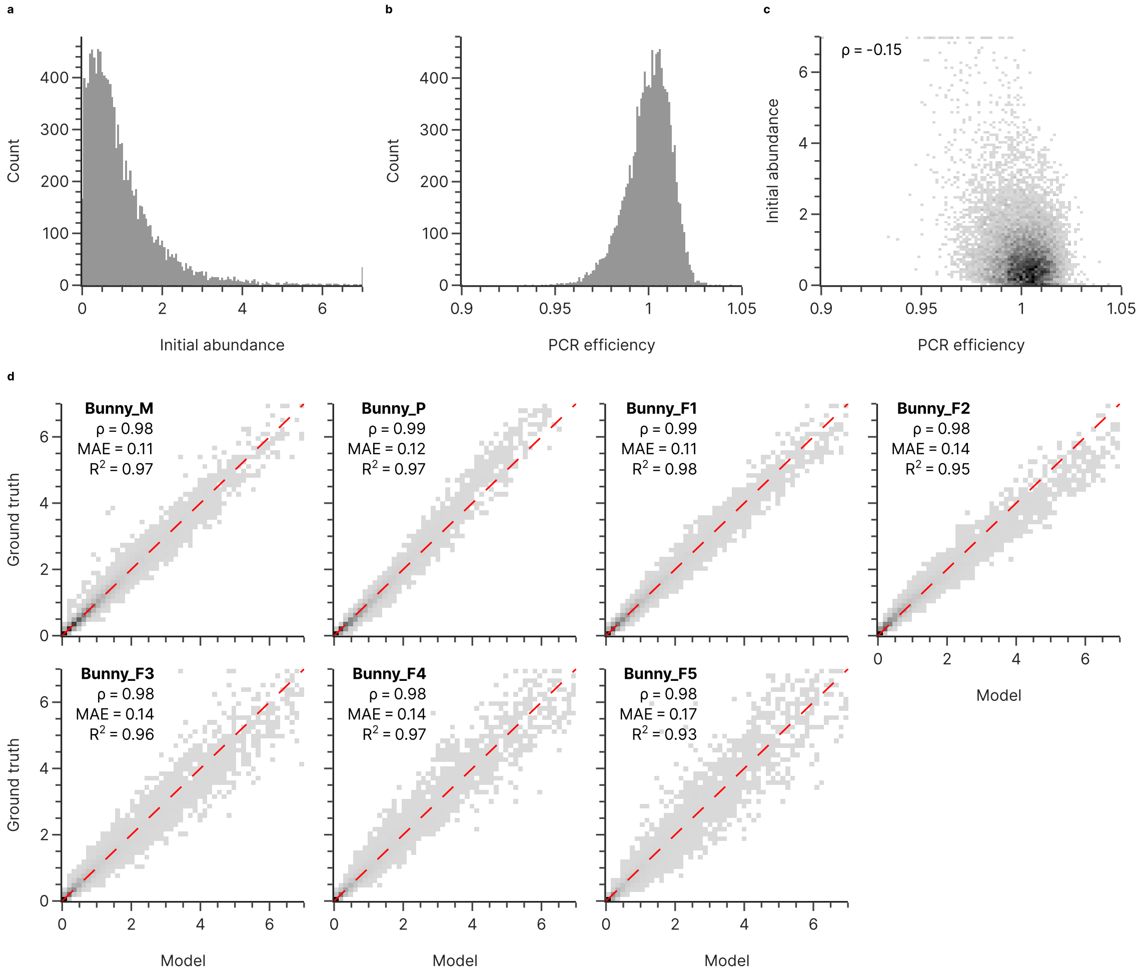

Supplementary Fig. 44 Results and performance of the two-parameter fitting for the Koch et al. dataset. The distributions of the fitted parameters of the initial abundance (a) and the amplification efficiency (b) are shown, as well as the correlation between both parameters (c). For each sequencing endpoint, the plots in (d) show the agreement between the experimental abundance (Ground truth, y-axis) and the expected abundance based on the PCR model and the fitted parameters (Model, x-axis). Panels (c) and (d) show relative density, and (d) includes the diagonal of perfect agreement (red, dashed line), as well as corresponding metrics (Spearman's rank correlation coefficient ρ, mean absolute error MAE, and coefficient of determination R^2). In panels (a-c), the values exceeding the axis have been clipped to the axis limits for visual clarity. Source data are provided as a Source Data file.

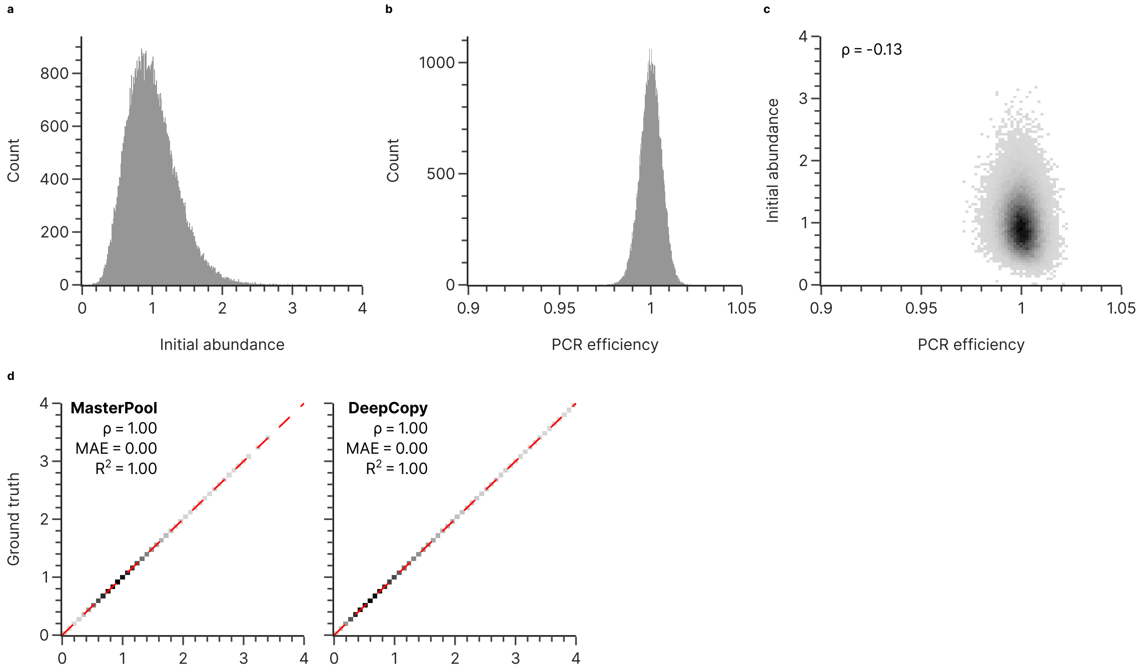

Supplementary Fig. 45 Results and performance of the two-parameter fitting for the Erlich et al. dataset. The distributions of the fitted parameters of the initial abundance (a) and the amplification efficiency (b) are shown, as well as the correlation between both parameters (c). For each sequencing endpoint, the plots in (d) show the agreement between the experimental abundance (Ground truth, y-axis) and the expected abundance based on the PCR model and the fitted parameters (Model, x-axis). Panels (c) and (d) show relative density, and (d) includes the diagonal of perfect agreement (red, dashed line), as well as corresponding metrics (Spearman's rank correlation coefficient ρ, mean absolute error MAE, and coefficient of determination R^2). In panels (a-c), the values exceeding the axis have been clipped to the axis limits for visual clarity. Source data are provided as a Source Data file.

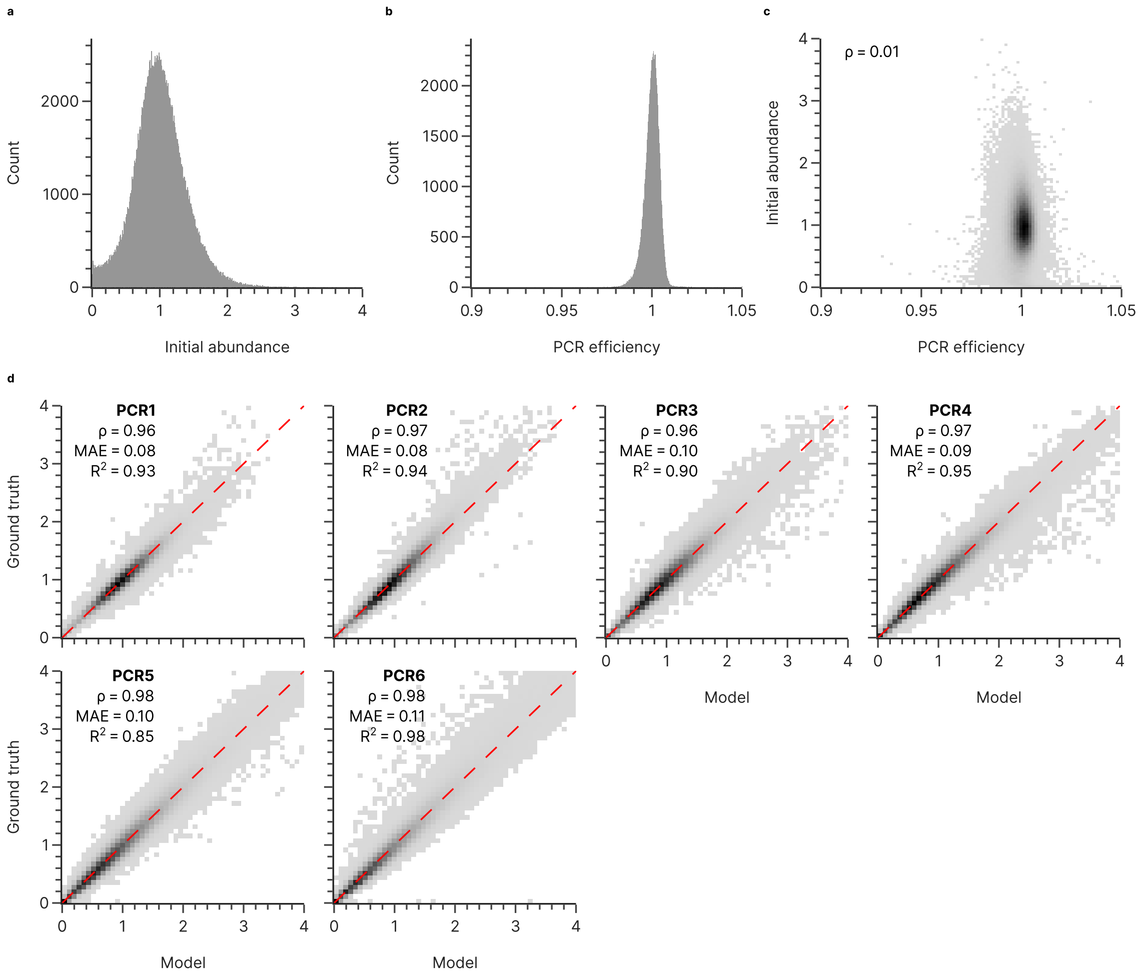

Supplementary Fig. 46 Results and performance of the two-parameter fitting for the Song et al. dataset. The distributions of the fitted parameters of the initial abundance (a) and the amplification efficiency (b) are shown, as well as the correlation between both parameters (c). For each sequencing endpoint, the plots in (d) show the agreement between the experimental abundance (Ground truth, y-axis) and the expected abundance based on the PCR model and the fitted parameters (Model, x-axis). Panels (c) and (d) show relative density, and (d) includes the diagonal of perfect agreement (red, dashed line), as well as corresponding metrics (Spearman's rank correlation coefficient ρ, mean absolute error MAE, and coefficient of determination R^2). In panels (a-c), the values exceeding the axis have been clipped to the axis limits for visual clarity. Source data are provided as a Source Data file.

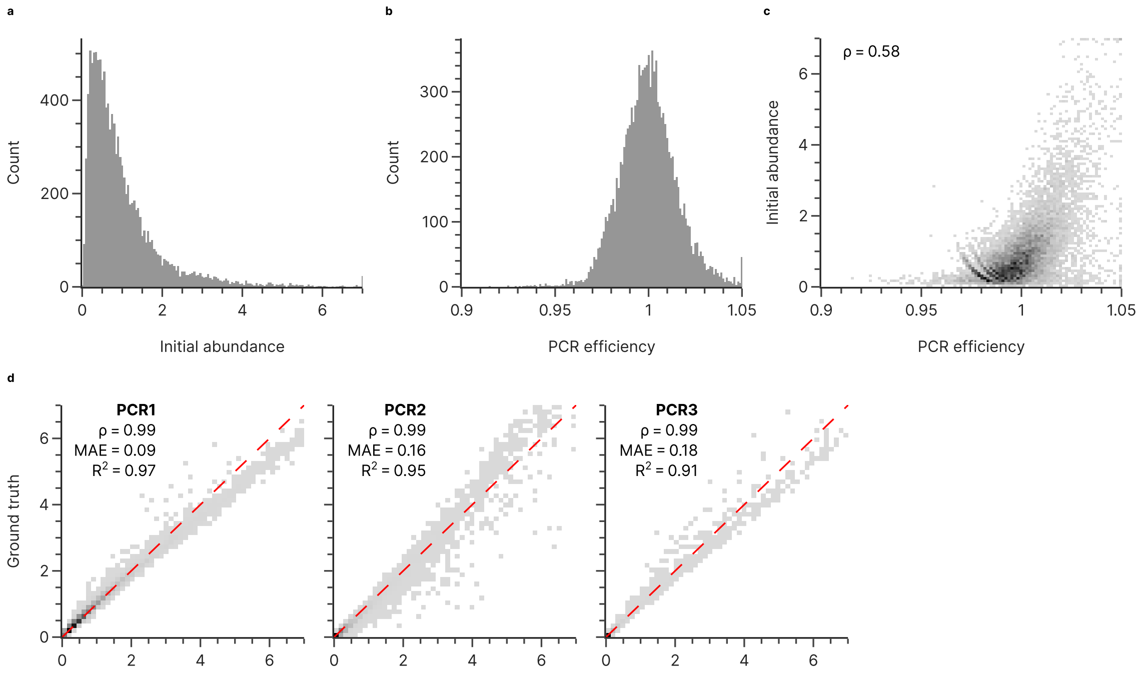

Supplementary Fig. 47 Results and performance of the two-parameter fitting for the Gao et al. dataset. The distributions of the fitted parameters of the initial abundance (a) and the amplification efficiency (b) are shown, as well as the correlation between both parameters (c). For each sequencing endpoint, the plots in (d) show the agreement between the experimental abundance (Ground truth, y-axis) and the expected abundance based on the PCR model and the fitted parameters (Model, x-axis). Panels (c) and (d) show relative density, and (d) includes the diagonal of perfect agreement (red, dashed line), as well as corresponding metrics (Spearman's rank correlation coefficient ρ, mean absolute error MAE, and coefficient of determination R^2). In panels (a-c), the values exceeding the axis have been clipped to the axis limits for visual clarity. Source data are provided as a Source Data file.

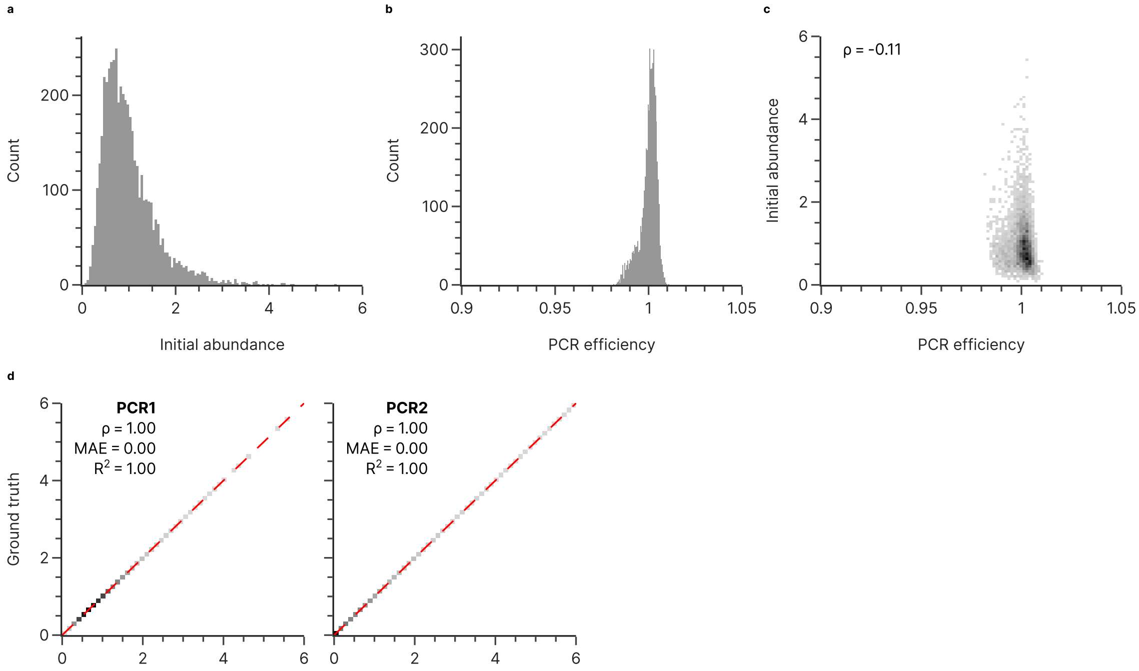

Supplementary Fig. 48 Results and performance of the two-parameter fitting for the Choi et al. dataset. The distributions of the fitted parameters of the initial abundance (a) and the amplification efficiency (b) are shown, as well as the correlation between both parameters (c). For each sequencing endpoint, the plots in (d) show the agreement between the experimental abundance (Ground truth, y-axis) and the expected abundance based on the PCR model and the fitted parameters (Model, x-axis). Panels (c) and (d) show relative density, and (d) includes the diagonal of perfect agreement (red, dashed line), as well as corresponding metrics (Spearman's rank correlation coefficient ρ, mean absolute error MAE, and coefficient of determination R^2). In panels (a-c), the values exceeding the axis have been clipped to the axis limits for visual clarity. Source data are provided as a Source Data file.

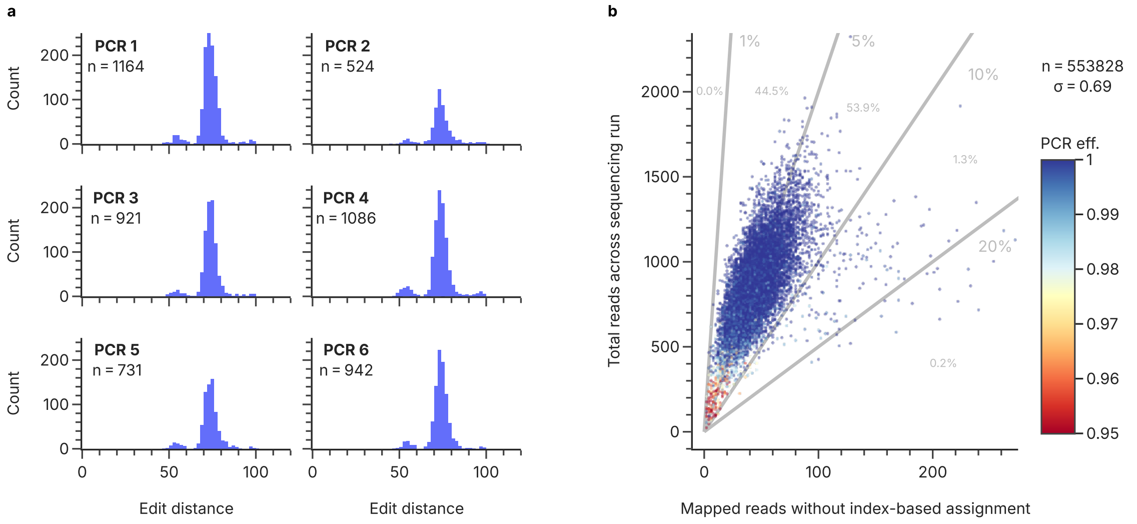

Supplementary Fig. 49 Occurrence of sequences of the GCall pool during demultiplexing and post-processing of the sequencing data. The distribution of minimum edit distances of all sequences removed during post-processing to the design sequences (a) shows that no real reads are lost during adapter removal and mapping. The occurrence of design sequences without experimental assignment after demultiplexing (b) shows that all sequences are found in the unassigned data at approximately the same rate, proportional to their overall presence. Source data are provided as a Source Data file.

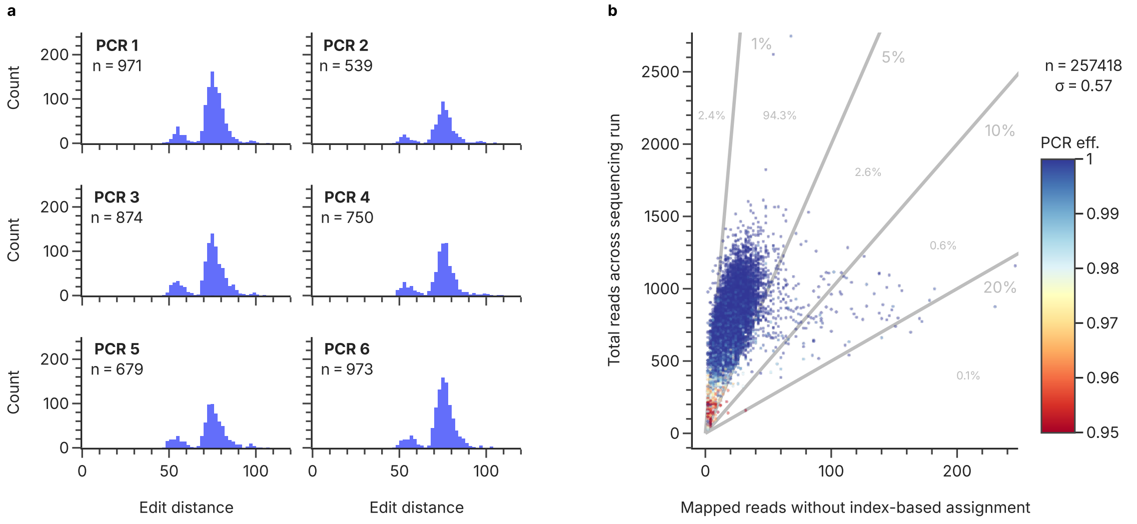

Supplementary Fig. 50 Occurrence of sequences of the GCfix pool during demultiplexing and post-processing of the sequencing data. The distribution of minimum edit distances of all sequences removed during post-processing to the design sequences (a) shows that no real reads are lost during adapter removal and mapping. The occurrence of design sequences without experimental assignment after demultiplexing (b) shows that all sequences are found in the unassigned data at approximately the same rate, proportional to their overall presence. Source data are provided as a Source Data file.

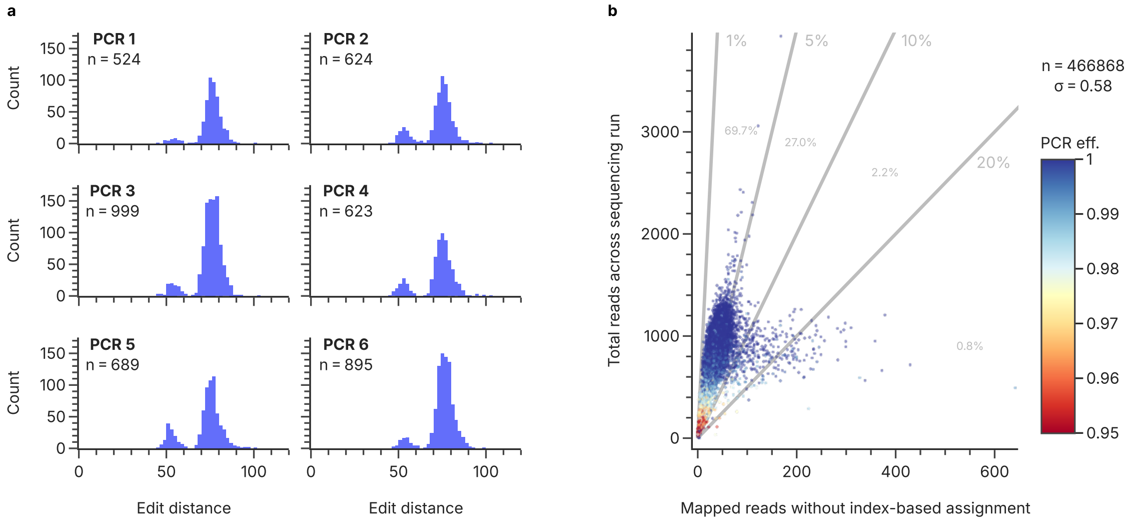

Supplementary Fig. 51 Occurrence of sequences of the validation pool using the GCall/GCfix conditions during demultiplexing and post-processing of the sequencing data. The distribution of minimum edit distances of all sequences removed during post-processing to the design sequences (a) shows that no real reads are lost during adapter removal and mapping. The occurrence of design sequences without experimental assignment after demultiplexing (b) shows that all sequences are found in the unassigned data at approximately the same rate, proportional to their overall presence. Source data are provided as a Source Data file.

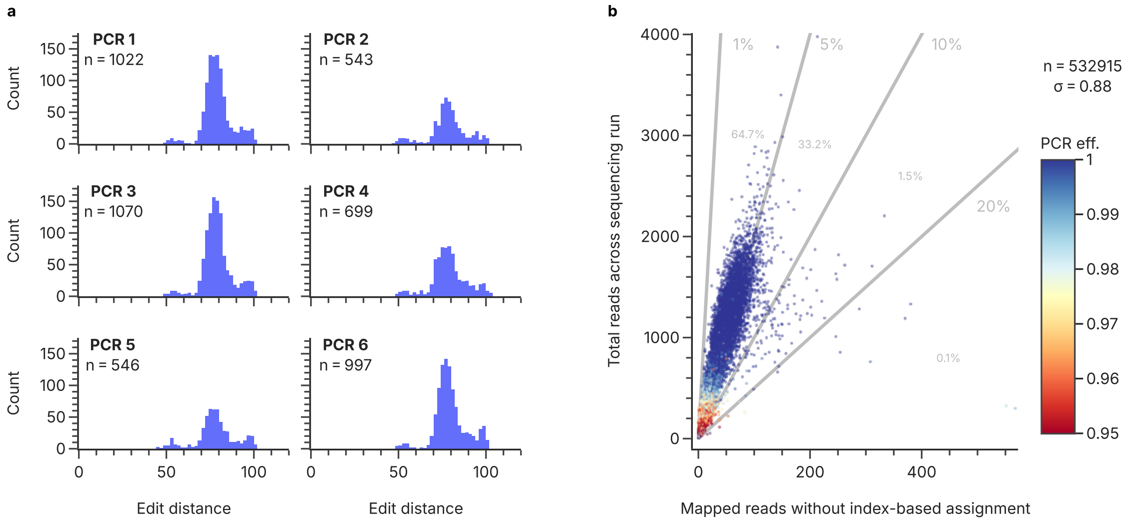

Supplementary Fig. 52 Occurrence of sequences of the validation pool using the Erlich et al. conditions during demultiplexing and post-processing of the sequencing data. The distribution of minimum edit distances of all sequences removed during post-processing to the design sequences (a) shows that no real reads are lost during adapter removal and mapping. The occurrence of design sequences without experimental assignment after demultiplexing (b) shows that all sequences are found in the unassigned data at approximately the same rate, proportional to their overall presence. Source data are provided as a Source Data file.

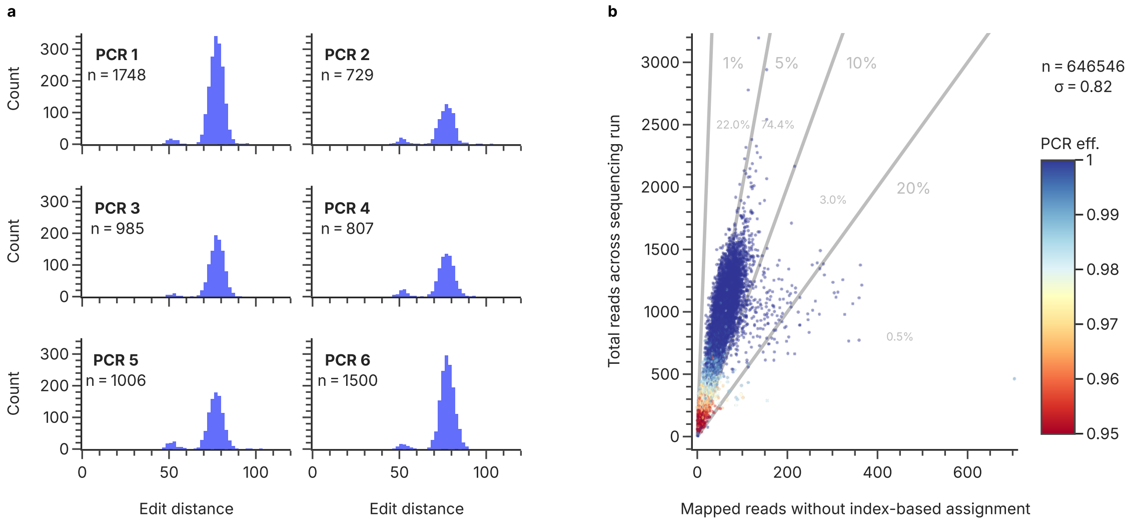

Supplementary Fig. 53 Occurrence of sequences of the validation pool using the Erlich et al. conditions, in the internal repeat of this experiment, during demultiplexing and post-processing of the sequencing data. The distribution of minimum edit distances of all sequences removed during post-processing to the design sequences (a) shows that no real reads are lost during adapter removal and mapping. The occurrence of design sequences without experimental assignment after demultiplexing (b) shows that all sequences are found in the unassigned data at approximately the same rate, proportional to their overall presence. Source data are provided as a Source Data file.

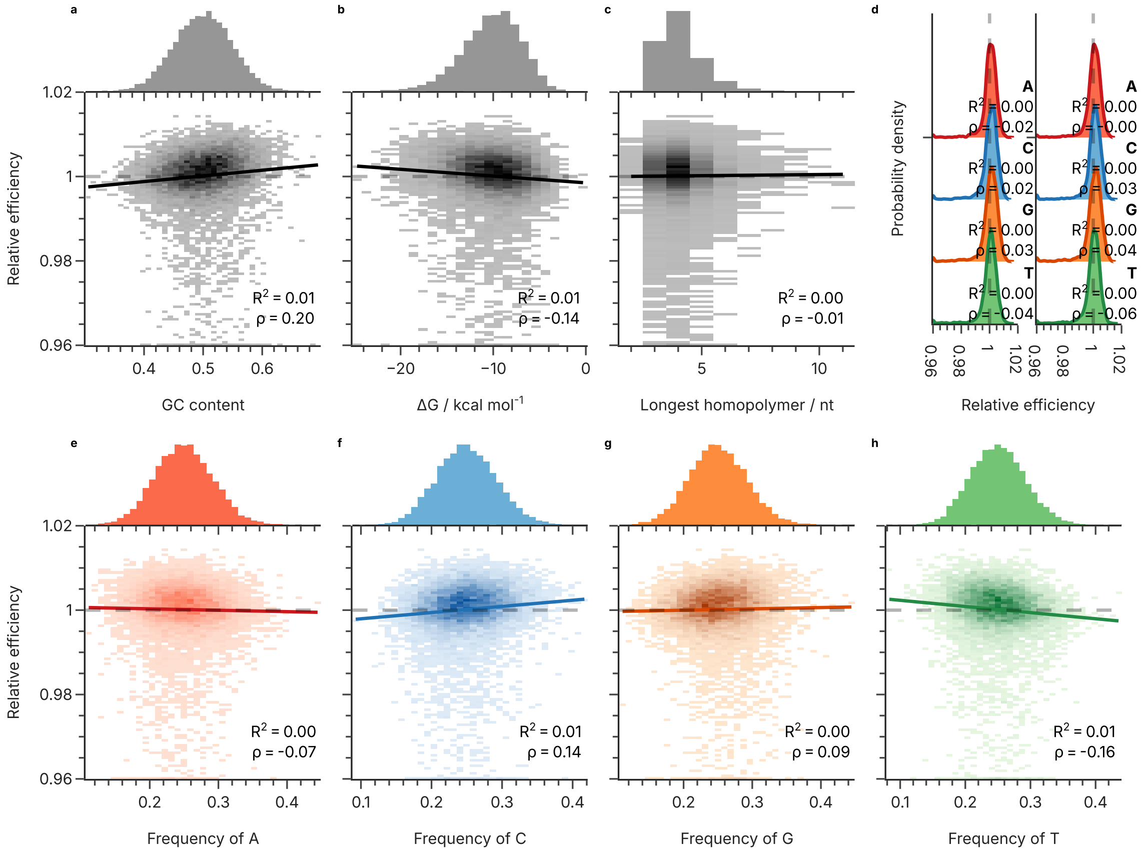

Supplementary Fig. 54 Correlation between estimated amplification efficiency and sequence properties for the GCall dataset. The sequence properties shown are the GC content (a), the free energy calculated by mfold (b), the length of the longest homopolymer in each sequence (c), the first and last nucleotide (d), as well as the frequency of A (e), C (f), G (g), and T (h) nucleotides in each sequence. Also shown are histograms of all sequence properties (top of panels), linear regressions (solid lines), the corresponding coefficients of determination (R^2), as well as Spearman's rank correlation coefficients (ρ). Source data are provided as a Source Data file.

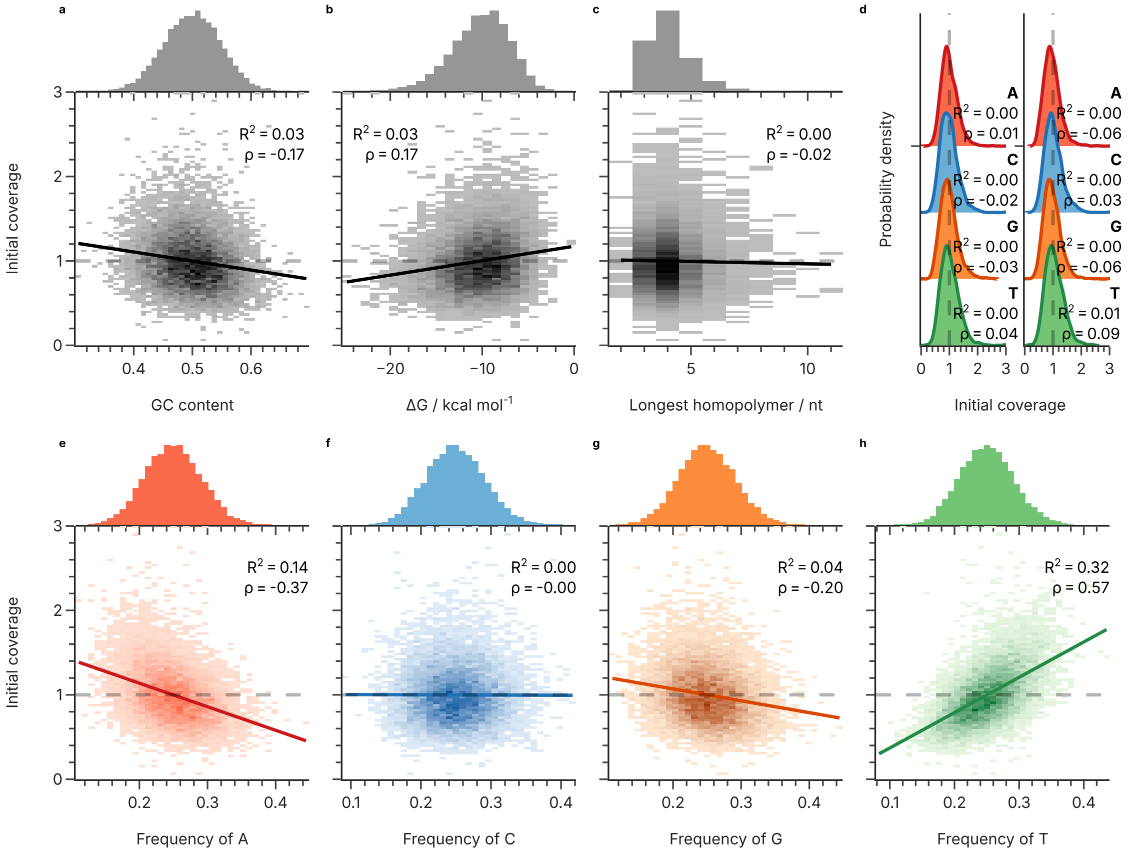

Supplementary Fig. 55 Correlation between estimated initial abundance and sequence properties for the GCall dataset. The sequence properties shown are the GC content (a), the free energy calculated by mfold (b), the length of the longest homopolymer in each sequence (c), the first and last nucleotide (d), as well as the frequency of A (e), C (f), G (g), and T (h) nucleotides in each sequence. Also shown are histograms of all sequence properties (top of panels), linear regressions (solid lines), the corresponding coefficients of determination (R^2), as well as Spearman's rank correlation coefficients (ρ). Source data are provided as a Source Data file.

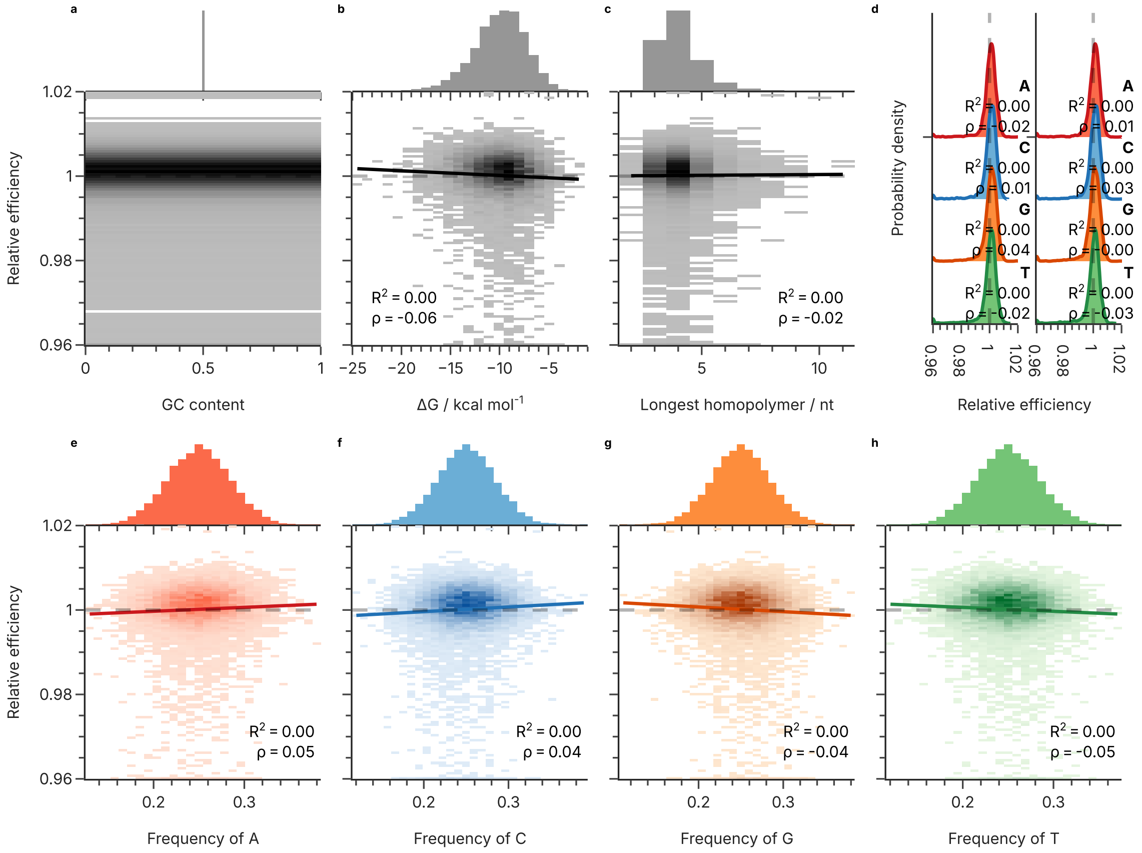

Supplementary Fig. 56 Correlation between estimated amplification efficiency and sequence properties for the GCfix dataset. The sequence properties shown are the GC content (a), the free energy calculated by mfold (b), the length of the longest homopolymer in each sequence (c), the first and last nucleotide (d), as well as the frequency of A (e), C (f), G (g), and T (h) nucleotides in each sequence. Also shown are histograms of all sequence properties (top of panels), linear regressions (solid lines), the corresponding coefficients of determination (R^2), as well as Spearman's rank correlation coefficients (ρ). Source data are provided as a Source Data file.

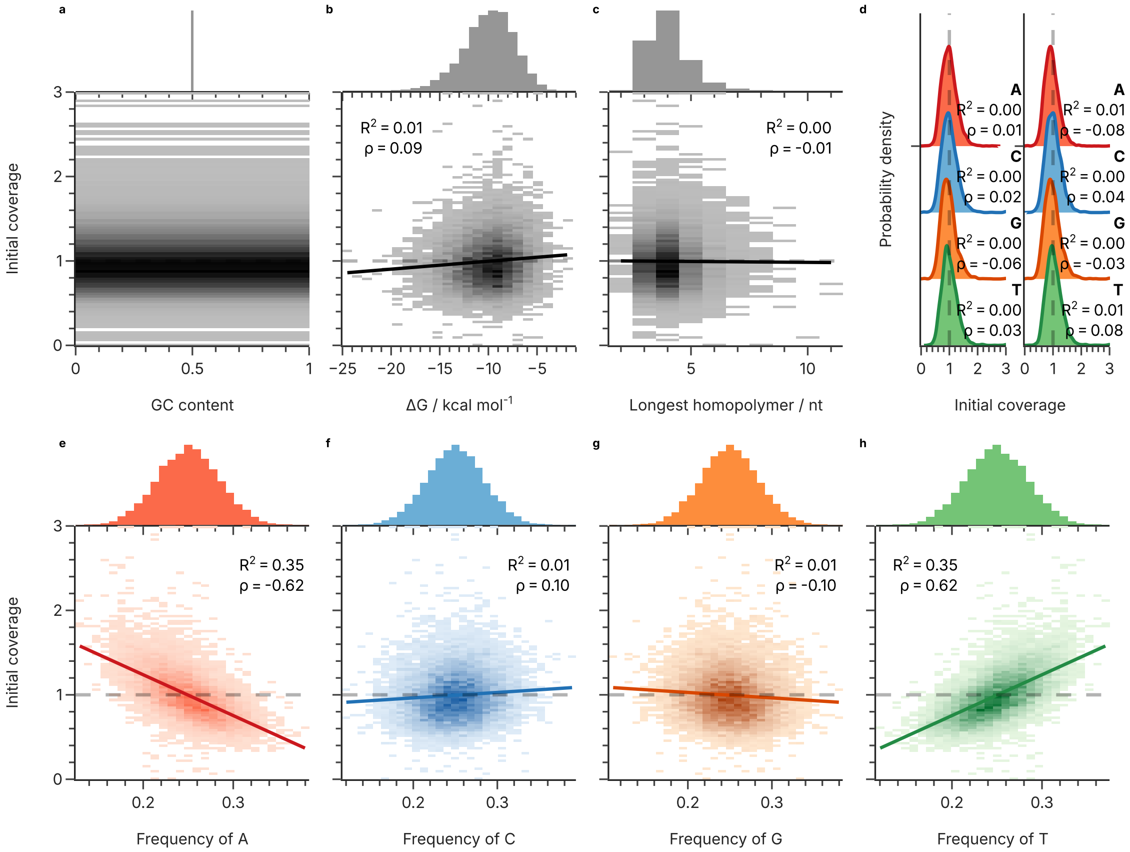

Supplementary Fig. 57 Correlation between estimated initial abundance and sequence properties for the GCfix dataset. The sequence properties shown are the GC content (a), the free energy calculated by mfold (b), the length of the longest homopolymer in each sequence (c), the first and last nucleotide (d), as well as the frequency of A (e), C (f), G (g), and T (h) nucleotides in each sequence. Also shown are histograms of all sequence properties (top of panels), linear regressions (solid lines), the corresponding coefficients of determination (R^2), as well as Spearman's rank correlation coefficients (ρ). Source data are provided as a Source Data file.

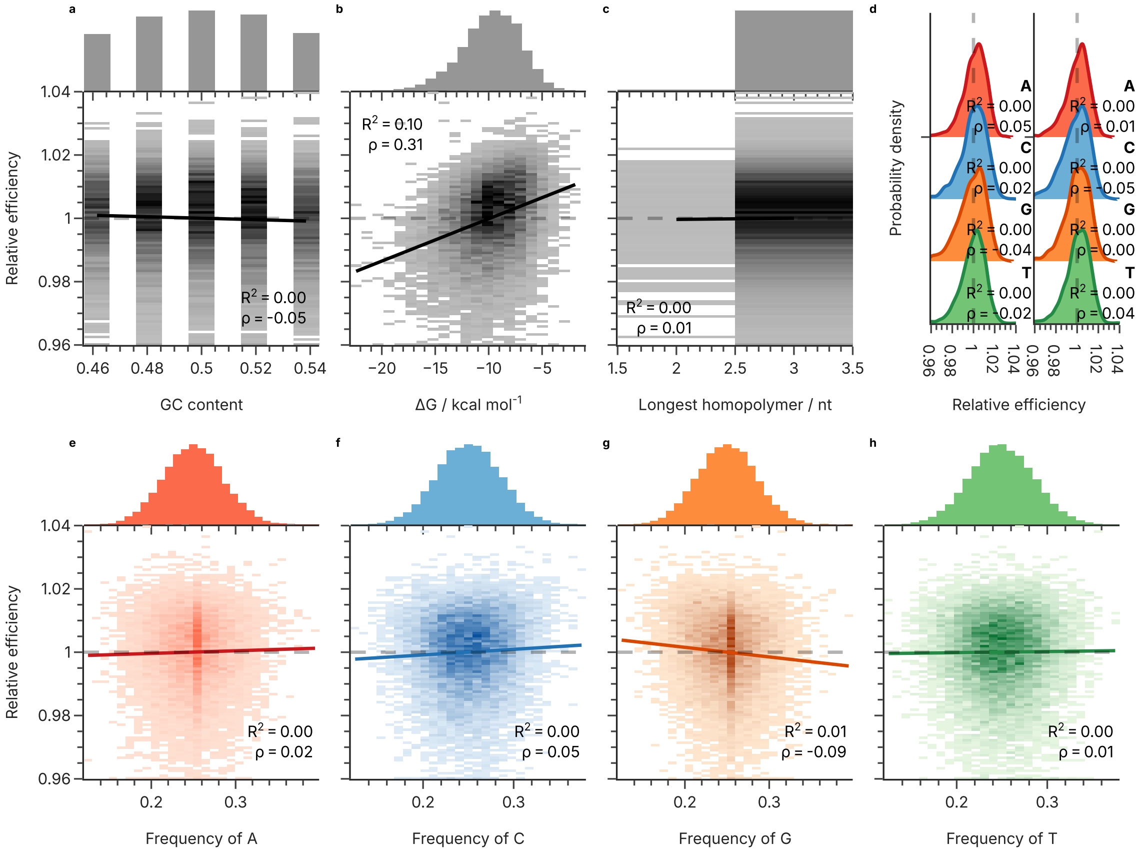

Supplementary Fig. 58 Correlation between estimated amplification efficiency and sequence properties for the Koch et al. dataset. The sequence properties shown are the GC content (a), the free energy calculated by mfold (b), the length of the longest homopolymer in each sequence (c), the first and last nucleotide (d), as well as the frequency of A (e), C (f), G (g), and T (h) nucleotides in each sequence. Also shown are histograms of all sequence properties (top of panels), linear regressions (solid lines), the corresponding coefficients of determination (R^2), as well as Spearman's rank correlation coefficients (ρ). Source data are provided as a Source Data file.

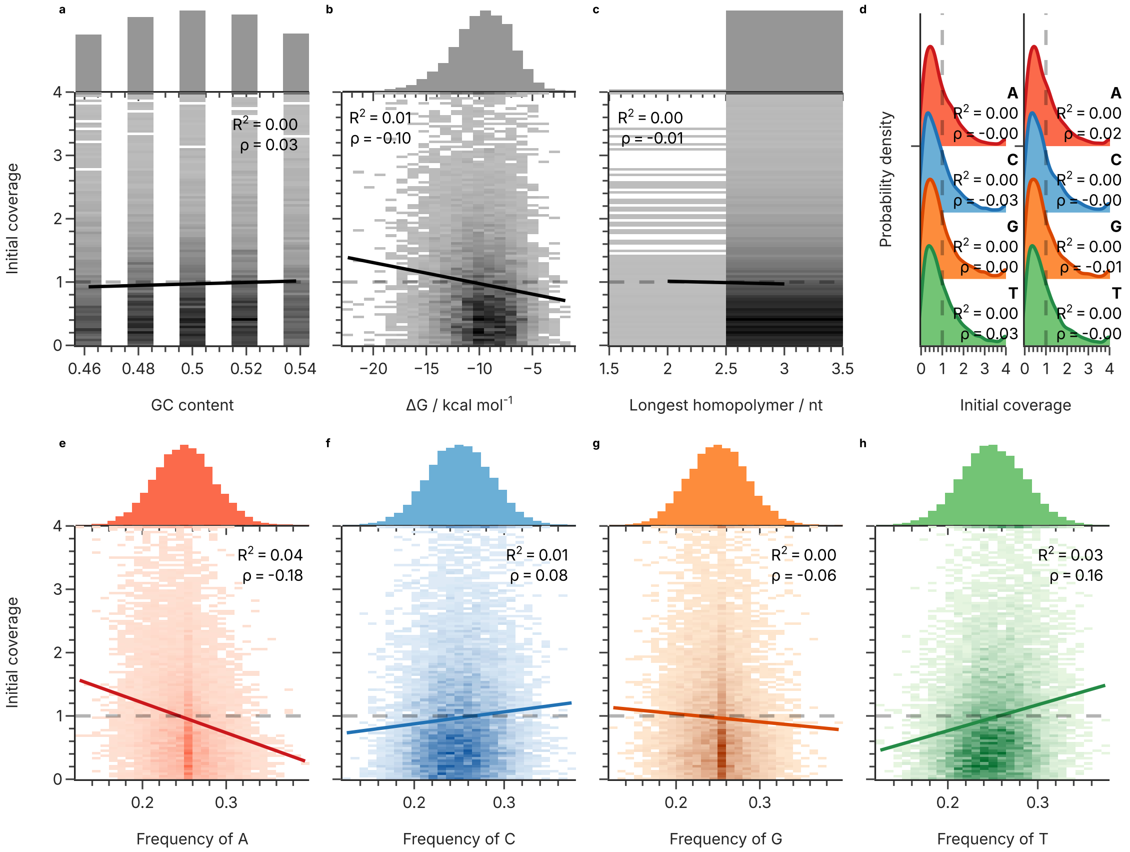

Supplementary Fig. 59 Correlation between estimated initial abundance and sequence properties for the Koch et al. dataset. The sequence properties shown are the GC content (a), the free energy calculated by mfold (b), the length of the longest homopolymer in each sequence (c), the first and last nucleotide (d), as well as the frequency of A (e), C (f), G (g), and T (h) nucleotides in each sequence. Also shown are histograms of all sequence properties (top of panels), linear regressions (solid lines), the corresponding coefficients of determination (R^2), as well as Spearman's rank correlation coefficients (ρ). Source data are provided as a Source Data file.

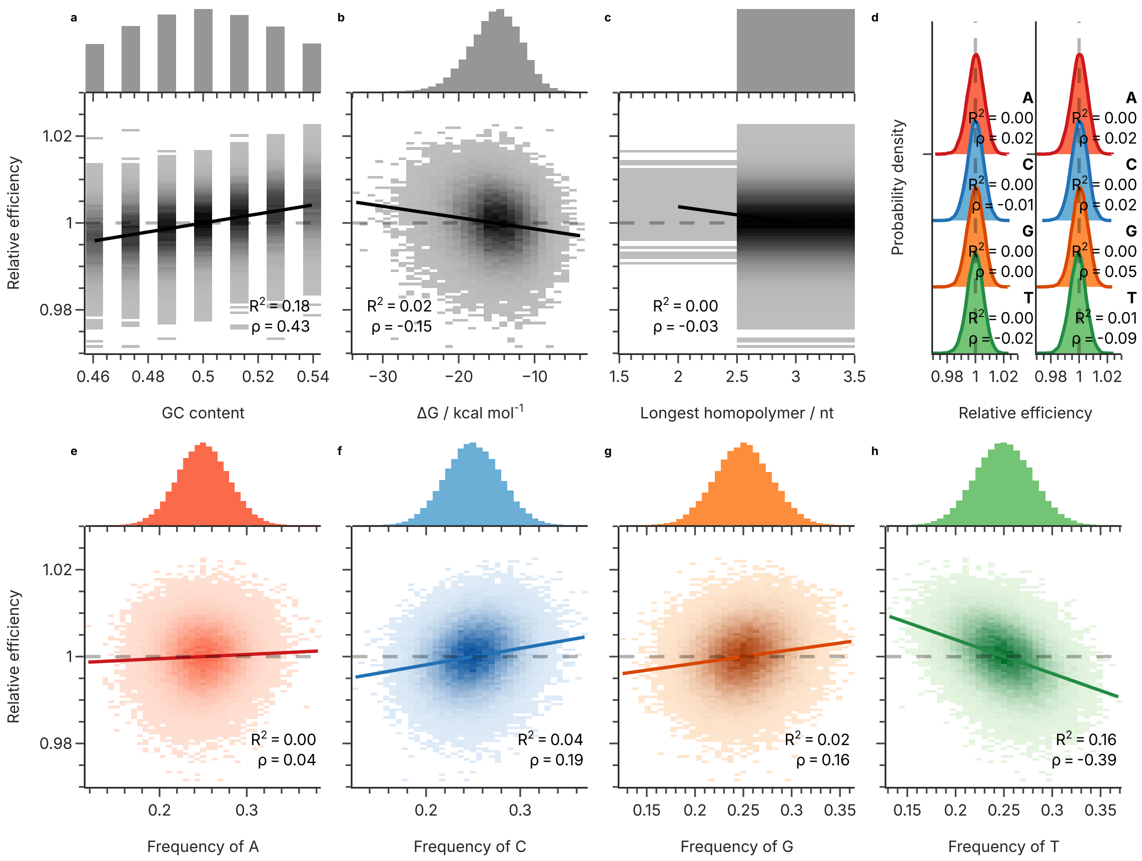

Supplementary Fig. 60 Correlation between estimated amplification efficiency and sequence properties for the Erlich et al. dataset. The sequence properties shown are the GC content (a), the free energy calculated by mfold (b), the length of the longest homopolymer in each sequence (c), the first and last nucleotide (d), as well as the frequency of A (e), C (f), G (g), and T (h) nucleotides in each sequence. Also shown are histograms of all sequence properties (top of panels), linear regressions (solid lines), the corresponding coefficients of determination (R^2), as well as Spearman's rank correlation coefficients (ρ). Source data are provided as a Source Data file.

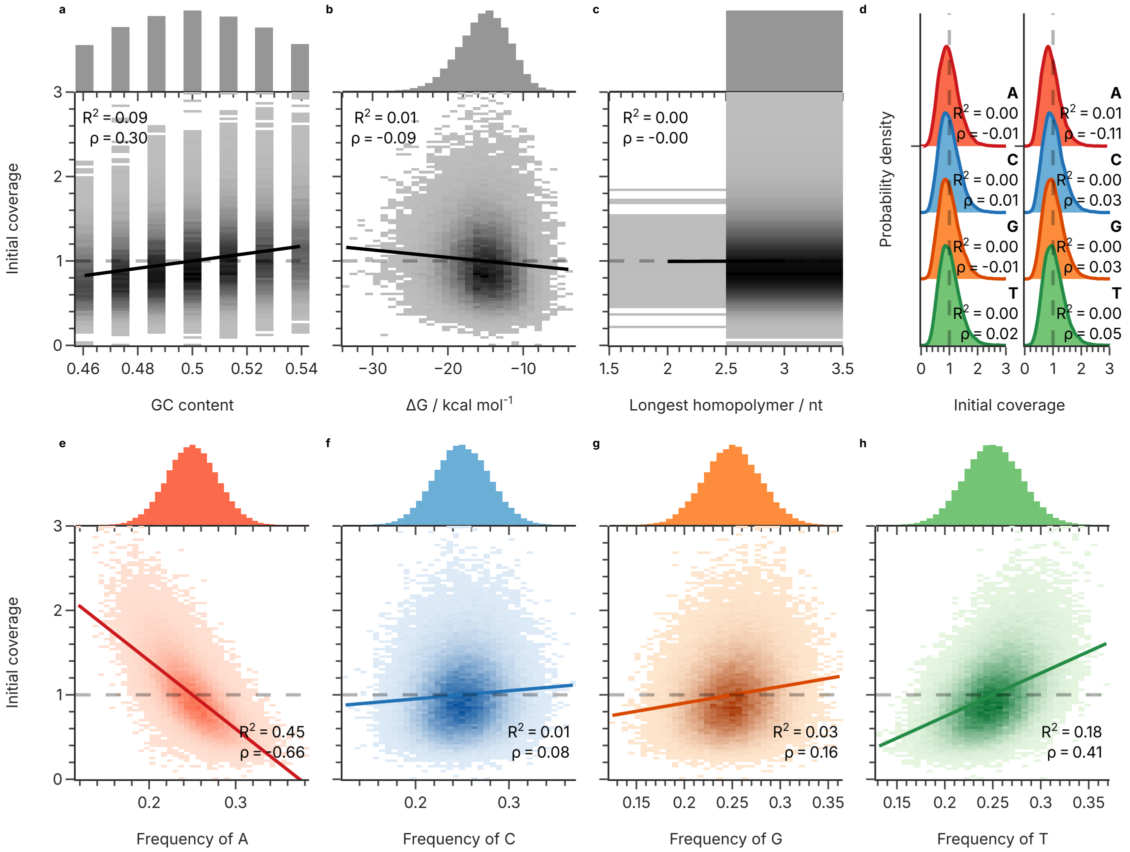

Supplementary Fig. 61 Correlation between estimated initial abundance and sequence properties for the Erlich et al. dataset. The sequence properties shown are the GC content (a), the free energy calculated by mfold (b), the length of the longest homopolymer in each sequence (c), the first and last nucleotide (d), as well as the frequency of A (e), C (f), G (g), and T (h) nucleotides in each sequence. Also shown are histograms of all sequence properties (top of panels), linear regressions (solid lines), the corresponding coefficients of determination (R^2), as well as Spearman's rank correlation coefficients (ρ). Source data are provided as a Source Data file.

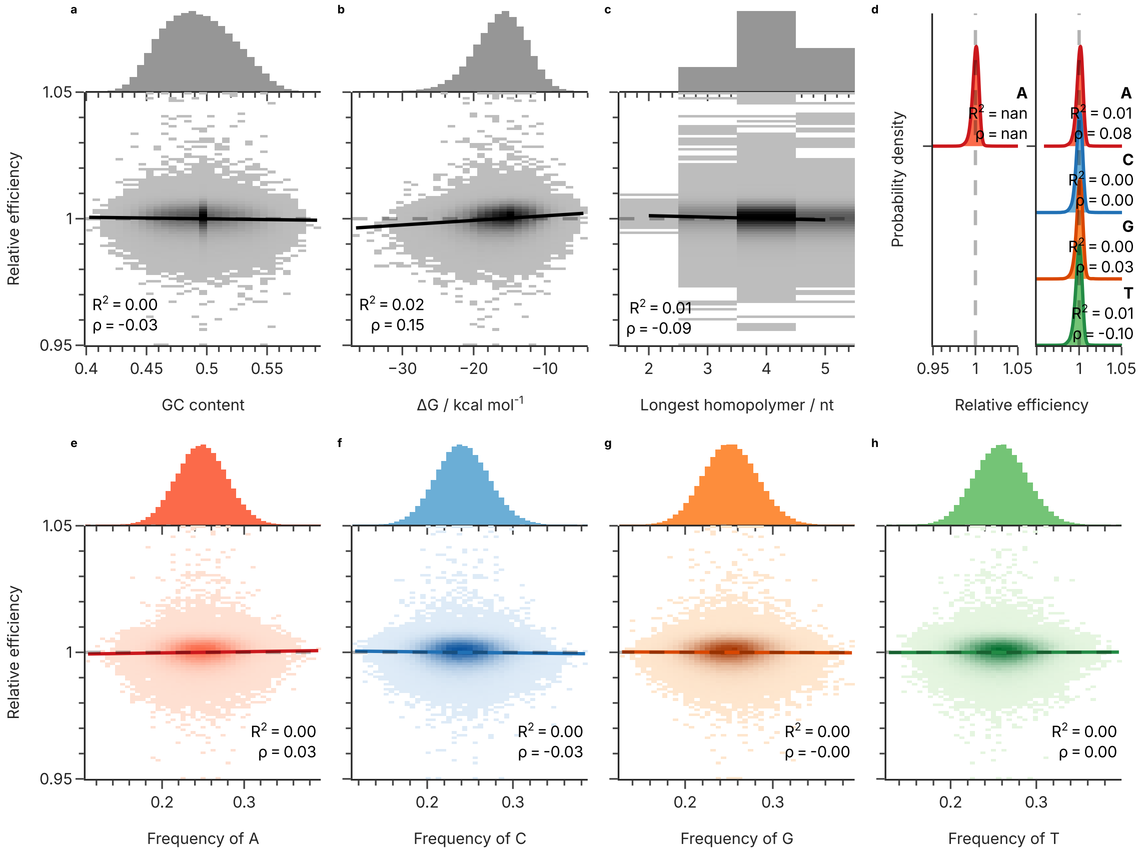

Supplementary Fig. 62 Correlation between estimated amplification efficiency and sequence properties for the Song et al. dataset. The sequence properties shown are the GC content (a), the free energy calculated by mfold (b), the length of the longest homopolymer in each sequence (c), the first and last nucleotide (d), as well as the frequency of A (e), C (f), G (g), and T (h) nucleotides in each sequence. Also shown are histograms of all sequence properties (top of panels), linear regressions (solid lines), the corresponding coefficients of determination (R^2), as well as Spearman's rank correlation coefficients (ρ). Source data are provided as a Source Data file.

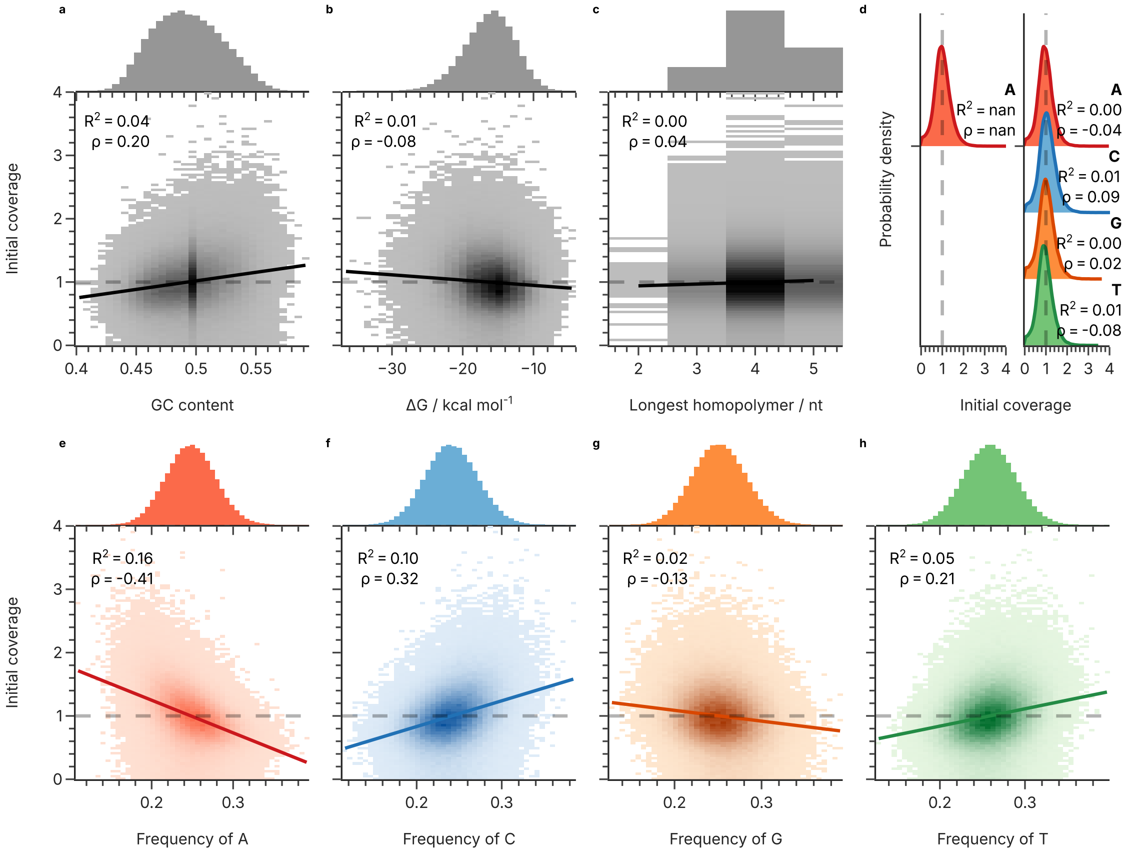

Supplementary Fig. 63 Correlation between estimated initial abundance and sequence properties for the Song et al. dataset. The sequence properties shown are the GC content (a), the free energy calculated by mfold (b), the length of the longest homopolymer in each sequence (c), the first and last nucleotide (d), as well as the frequency of A (e), C (f), G (g), and T (h) nucleotides in each sequence. Also shown are histograms of all sequence properties (top of panels), linear regressions (solid lines), the corresponding coefficients of determination (R^2), as well as Spearman's rank correlation coefficients (ρ). Source data are provided as a Source Data file.

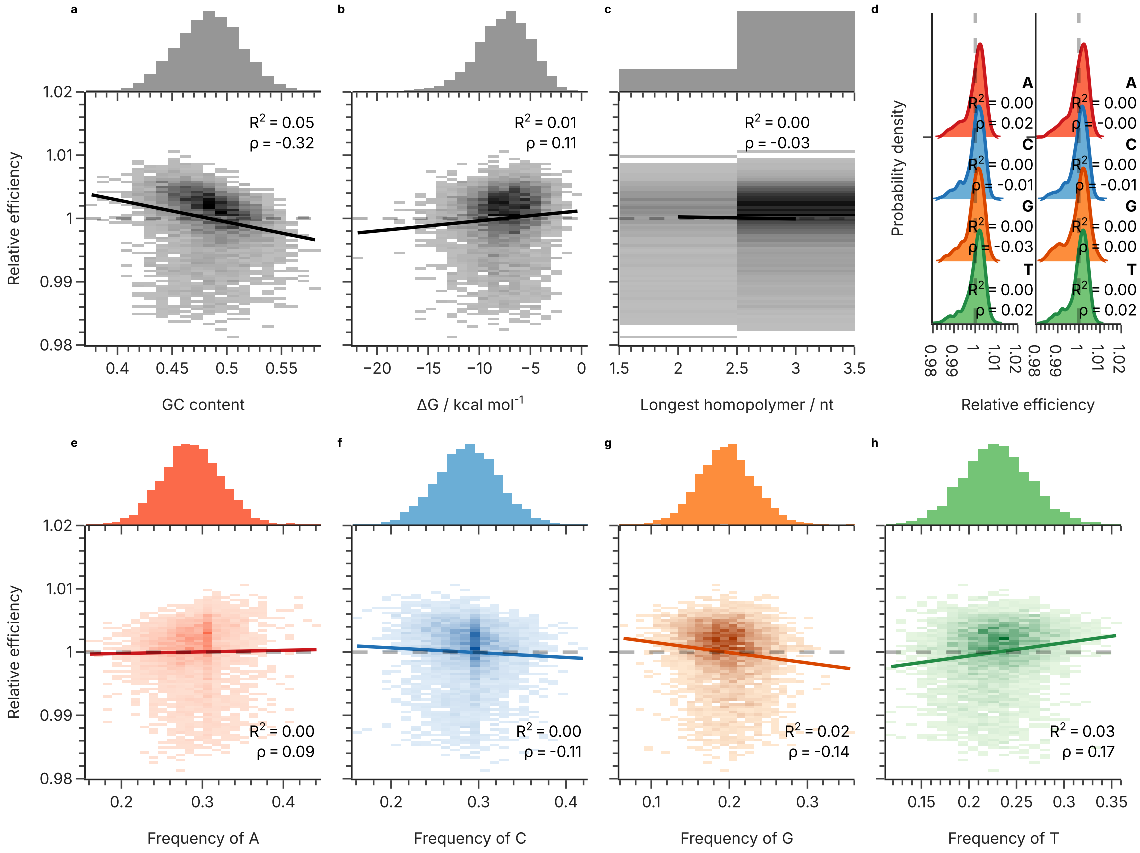

Supplementary Fig. 64 Correlation between estimated amplification efficiency and sequence properties for the Choi et al. dataset. The sequence properties shown are the GC content (a), the free energy calculated by mfold (b), the length of the longest homopolymer in each sequence (c), the first and last nucleotide (d), as well as the frequency of A (e), C (f), G (g), and T (h) nucleotides in each sequence. Also shown are histograms of all sequence properties (top of panels), linear regressions (solid lines), the corresponding coefficients of determination (R^2), as well as Spearman's rank correlation coefficients (ρ). Source data are provided as a Source Data file.

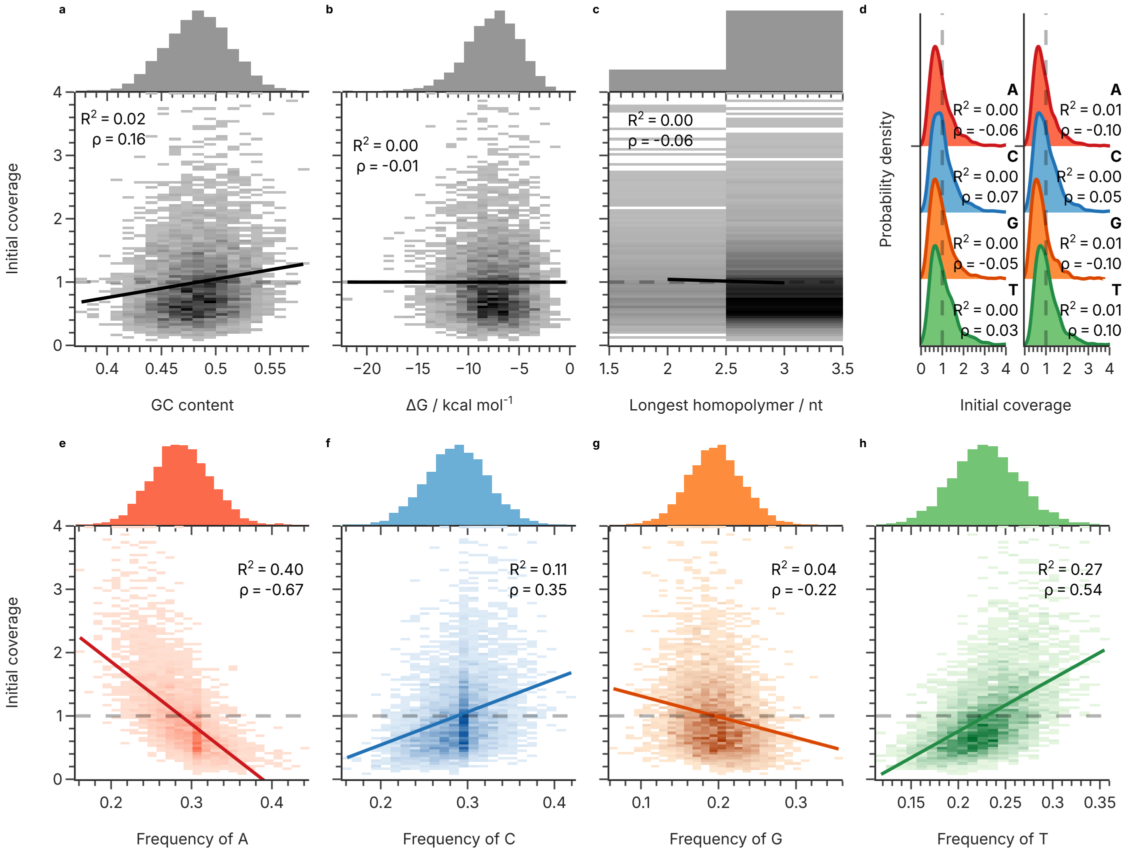

Supplementary Fig. 65 Correlation between estimated initial abundance and sequence properties for the Choi et al. dataset. The sequence properties shown are the GC content (a), the free energy calculated by mfold (b), the length of the longest homopolymer in each sequence (c), the first and last nucleotide (d), as well as the frequency of A (e), C (f), G (g), and T (h) nucleotides in each sequence. Also shown are histograms of all sequence properties (top of panels), linear regressions (solid lines), the corresponding coefficients of determination (R^2), as well as Spearman's rank correlation coefficients (ρ). Source data are provided as a Source Data file.

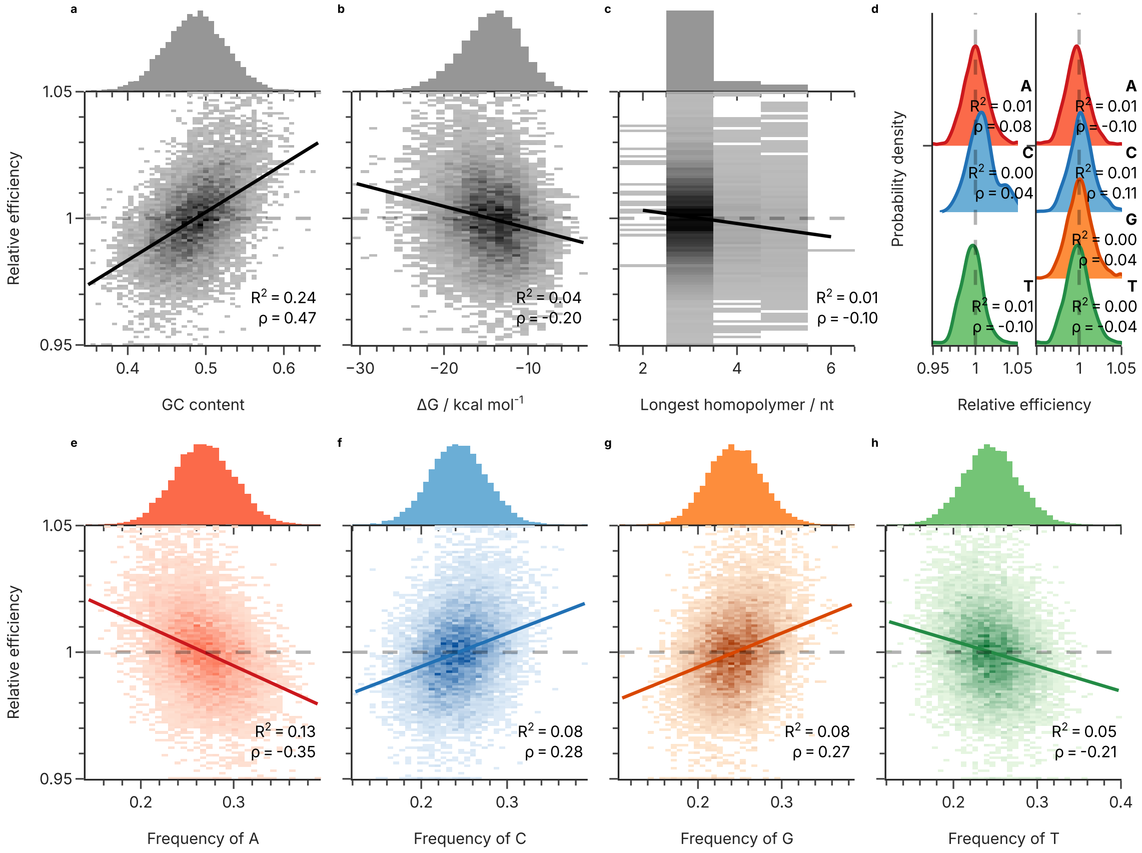

Supplementary Fig. 66 Correlation between estimated amplification efficiency and sequence properties for the Gao et al. dataset. The sequence properties shown are the GC content (a), the free energy calculated by mfold (b), the length of the longest homopolymer in each sequence (c), the first and last nucleotide (d), as well as the frequency of A (e), C (f), G (g), and T (h) nucleotides in each sequence. Also shown are histograms of all sequence properties (top of panels), linear regressions (solid lines), the corresponding coefficients of determination (R^2), as well as Spearman's rank correlation coefficients (ρ). Source data are provided as a Source Data file.

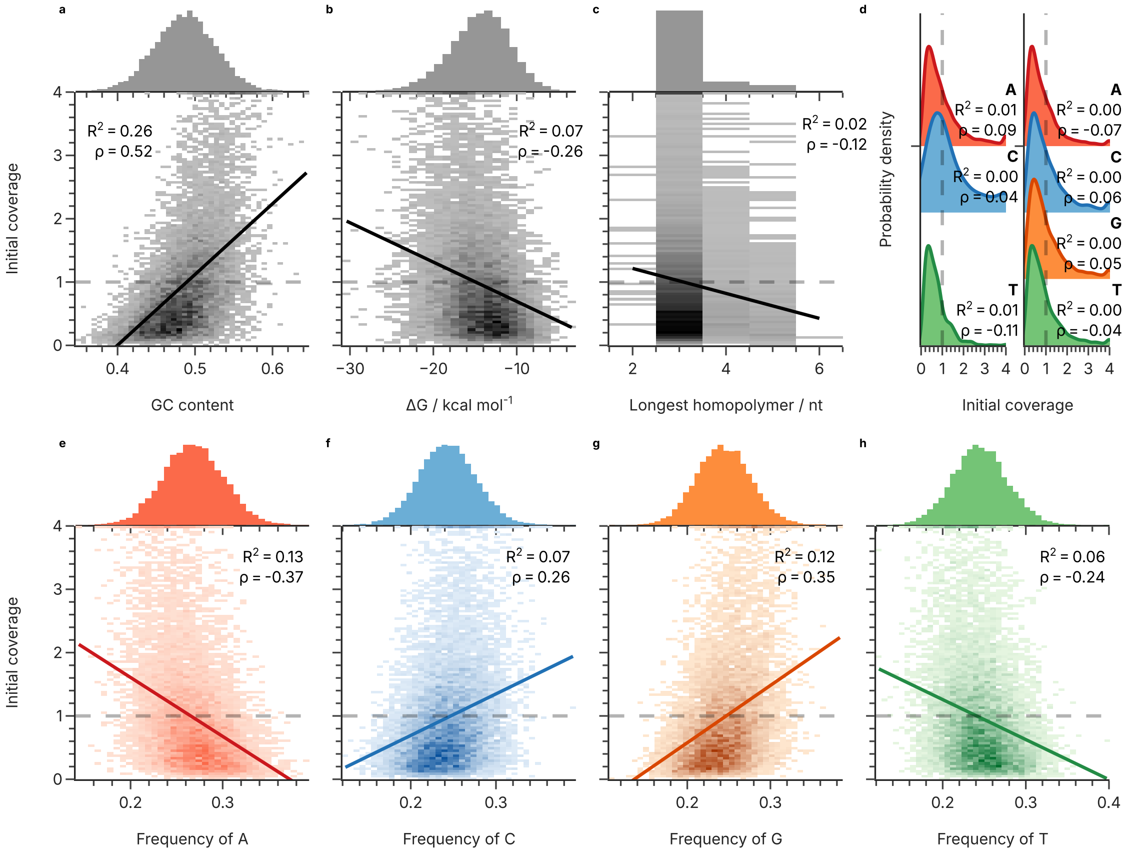

Supplementary Fig. 67 Correlation between estimated initial abundance and sequence properties for the Gao et al. dataset. The sequence properties shown are the GC content (a), the free energy calculated by mfold (b), the length of the longest homopolymer in each sequence (c), the first and last nucleotide (d), as well as the frequency of A (e), C (f), G (g), and T (h) nucleotides in each sequence. Also shown are histograms of all sequence properties (top of panels), linear regressions (solid lines), the corresponding coefficients of determination (R^2), as well as Spearman's rank correlation coefficients (ρ). Source data are provided as a Source Data file.

Supplementary Tables

Hyperparameter	Search values
Model	RNN, LSTM, GRU
Number of layers	1, 2, 3
Embedding dimension	32, 64, 128
Hidden dimension	32, 64, 128
Learning rate	10^{-3}, 10^{-4}, 10^{-5}
Batch size	64, 128, 256
Weight decay	0, 10^{-2}, 10^{-3}, 10^{-4}

Supplementary Table 1 Hyperparameter grid and ranges for the hyperparameter search of the RNN-based model.

Supplementary Table 2 Overview of qPCR results.

		GCall	#11493	#00006	#09807	#01634
	Category	reference	low ϵ	medium ϵ	low ϵ	high ϵ
Exp. 1	Slope	3.52	3.62	3.59	4.49	3.50
	Intercept	12.5	6.40	4.01	6.31	4.35
	R^2	>0.999	0.999	>0.999	0.994	>0.999
	ϵ	92.2%	88.8%	89.8%	67.0%	92.9%
Exp. 2	Slope	3.50	3.61	3.56	4.44	3.55
	Intercept	12.6	6.33	4.46	6.29	4.20
	R^2	0.999	>0.999	>0.999	0.991	>0.999
	ϵ	93.0%	89.2%	91.0%	68.0%	91.3%
Exp. 3	Slope	3.42	3.60	3.54	4.41	3.49
	Intercept	12.7	6.34	4.07	6.24	4.33
	R^2	>0.999	>0.999	>0.999	0.993	>0.999
	ϵ	96.0%	89.6%	91.8%	68.6%	93.3%
Overall	$\bar{\epsilon}$	93.7%	89.2%	90.8%	67.8%	92.5%
	SEM	1.14%	0.24%	0.58%	0.48%	0.61%

Supplementary Table 3 Multiple comparisons of the qPCR results using Tukey's range test. Tukey's range test controls for family-wise error rate in multiple comparisons.

Sample 1	Sample 2	$\Delta\epsilon$	p-value	95% conf. interval	
#00006	#01634	1.67%	0.4521	-1.48%	4.82%
#00006	#09807	-23.00%	3×10^{-9}	-26.14%	-19.85%
#00006	#11493	-1.64%	0.4671	-4.79%	1.51%
#00006	GCall	2.85%	0.0812	-0.30%	6.00%
#01634	#09807	-24.67%	1×10^{-9}	-27.81%	-21.52%
#01634	#11493	-3.31%	0.0385	-6.46%	-0.16%
#01634	GCall	1.18%	0.7341	-1.97%	4.33%
#09807	#11493	21.36%	6×10^{-9}	18.21%	24.50%
#09807	GCall	25.84%	9×10^{-10}	22.70%	28.99%
#11493	GCall	4.49%	0.0059	1.34%	7.64%

Supplementary Table 4 This table presents the positive class prevalence of each dataset for different statistically defined thresholds to categorize low-efficiency and normal-efficiency DNA sequences.

**Dataset**	Choi_et_al	Erlich_et_al	GCall	GCfix	Gao_et_al	Koch_et_al	Song_et_al
1 σ	0.146905	0.156250	0.059843	0.051192	0.141678	0.151776	0.020000
2 σ	0.059601	0.025861	0.027171	0.024679	0.016688	0.038278	0.034714
3 σ	0.011962	0.002556	0.016086	0.015258	0.003651	0.007020	0.008859

Supplementary Table 5 Spearman rank correlations of the estimated parameters of the three external validation datasets.

Group	Initial abundance			PCR efficiency		
	GCall/fix	Erlich (ext.)	Erlich (int.)	GCall/fix	Erlich (ext.)	Erlich (int.)
GCall/fix	1.00	0.30	0.28	1.00	-0.23	-0.22
Erlich (ext.)	0.30	1.00	0.63	-0.23	1.00	0.91
Erlich (int.)	0.28	0.63	1.00	-0.22	0.91	1.00

Supplementary Table 6 Sequences used in the study.

Name	Description	Sequence (5'-3')
0F	Forward primer	ACACGACGCTCTTCCGATCT
0R	Reverse primer	AGACGTGTGCTCTTCCGATCT
0F_4W	Degenerate forward primer	WWWWACACGACGCTCTTCCGATCT
0R_4W	Degenerate reverse primer	WWWWAGACGTGTGCTCTTCCGATCT
2FUF	Forward sequencing primer	AATGATACGGCGACCACCGAGATCTACACTCTTTCCCTACA<br>CGACGCTCTTCCGATCT
2RIF-GM5	Indexed reverse sequencing primer	CAAGCAGAAGACGGCATACGAGATCACTGTGTGACTGGAGT<br>TCAGACGTGTGCTCTTCCGATCT
2RIF-GM7	Indexed reverse sequencing primer	CAAGCAGAAGACGGCATACGAGATGATCTGGTGACTGGAGT<br>TCAGACGTGTGCTCTTCCGATCT
2RIF-GM8	Indexed reverse sequencing primer	CAAGCAGAAGACGGCATACGAGATTCAAGTGTGACTGGAGT<br>TCAGACGTGTGCTCTTCCGATCT
2RIF-GM10	Indexed reverse sequencing primer	CAAGCAGAAGACGGCATACGAGATAAGCTAGTGACTGGAGT<br>TCAGACGTGTGCTCTTCCGATCT
2RIF-GM11	Indexed reverse sequencing primer	CAAGCAGAAGACGGCATACGAGATGTAGCCGTGACTGGAGT<br>TCAGACGTGTGCTCTTCCGATCT
2RIF-GM12	Indexed reverse sequencing primer	CAAGCAGAAGACGGCATACGAGATTACAAGGTGACTGGAGT<br>TCAGACGTGTGCTCTTCCGATCT
2RIF-GM17	Indexed reverse sequencing primer	CAAGCAGAAGACGGCATACGAGATCTCTACGTGACTGGAGT<br>TCAGACGTGTGCTCTTCCGATCT
#11493	qPCR test sequence	ACACGACGCTCTTCCGATCTCGTGTATAGGCTGACTGTTAT<br>GTTCGTGCAGCACGCTGCATGGCTGATCGTATGTACTTGGG<br>CACTCTAAAGATTCAAGGCTAAGGAAGAAGGATAACGCTAC<br>CCAACAGATCGGAAGAGCACACGTCT
#00006	qPCR test sequence	ACACGACGCTCTTCCGATCTTATGTACTCTTGCACGCTTGG<br>CGGGTGTTAAACTTCTGGTTCCGACCTAGCGCTCGTGCCTT<br>CCGGTGAATGTAGGTTGCCGAATGACACATCCATGCCCTAT<br>TCAGCAGATCGGAAGAGCACACGTCT
#09807	qPCR test sequence	ACACGACGCTCTTCCGATCTGAGTTCAAGCGCCGTGTAGCC<br>TGATCTGGTCCTTATACTAGCCTGTTCACAAAGATATAGAT<br>CTGTATAAACAGCGCCATTCAAGCTGAGAGAGAGGGCCCAG<br>GGGCCAGATCGGAAGAGCACACGTCT
#01634	qPCR test sequence	ACACGACGCTCTTCCGATCTTTGCTTTGCTGTTGTGCGGTA<br>CACCCCAACGTGATCTTTAGTCCTTGGAAAGTCCACCACAT<br>CTCTTGTAGGGCTAGCTTATTCTAGAACAACAGCAAGGGCT<br>GCCACAGATCGGAAGAGCACACGTCT
no motif	Primer3 sequence wo. motif	CGGACGAATTGCGAATGTTCAGTGTACCGTAAAGATATGGT<br>ATCGTTTGACAAAGAGCCACCA
5' motif	Primer3 sequence with 5'-motif	CGGACGAATTGCGAATGTTCCGTCCGCCGTAAAGATATGGT<br>ATCGTTTGACAAAGAGCCACCA
3' motif	Primer3 sequence with 3'-motif	CGGACGAATTGCGAATGTTCAGTGTACCGTAAAGATATGGT<br>GGCGTTTGACAAAGAGCCACCA
Primer3-F	Primer3 forward primer	CGGACGAATTGCGAATGTTC
Primer3-R	Primer3 reverse primer	TGGTGGCTCTTTGTCAAACG

Supplementary Table 7 Overview of datasets.

Dataset	GCall	GCfix	Koch et al.	Erlich et al.	Song et al.	Choi et al.	Gao et al.
Source	PRJEB65931	PRJEB65931	PRJEB35217	PRJEB19305<br>PRJEB19307	10.6084/m9.figshare.16727122<br>10.6084/m9.figshare.17193128<br>10.6084/m9.figshare.18515045	PRJNA555140	pers. comm.
**Experimental design**							
#Experiments	6	6	7	2	6	2	3
Cycle counts	15-90	15-90	44-119	10, 100	30-180	17, 340	10, 50, 100
Synthesis provider	Twist	Twist	CustomArray	Twist	Twist	CustomArray	Twist
Polymerase	KAPA FAST	KAPA FAST	KAPA FAST	Q5 HiFi	error-prone polymerase	KAPA HiFi	Q5 HiFi
**Pool design**							
#Sequences	12000	12000	12000	72000	210000	5173	11520
Seq. length	108	108	104	152	164	93	146
Seq. constraints	-	GC	GC, HP	GC, HP	GC, HP	-	GC, motifs
Seq. randomized	Yes	Yes	No	No	No	No	No
**Pre-processing**							
#Seq. removed	2	6	35	0	53	408	15
%Seq. removed	0.017%	0.05%	0.29%	0%	0.025%	7.9%	0.13%
**Parameter fit**							
R^2_{baseline}	0.750	0.758	0.868	0.755	0.424	0.684	0.434
R^2_{model}	0.859	0.868	0.957	1.000	0.967	1.000	0.911
df_1	11998	11994	11965	72000	209947	4765	11505
df_2	47992	47976	59825	0	839788	0	11505
$F(df_1, df_2)$	3.08	3.33	10.2	-	65.8	-	5.38

References

1. Tarawneh, A. S., Hassanat, A. B., Altarawneh, G. A. & Almuhaimeed, A. Stop Oversampling for Class Imbalance Learning: A Review. *IEEE Access* **10,** 47643–47660 (2022).
2. Stewart, L., Bach, F., Berthet, Q. & Vert, J.-P. *Regression as classification: Influence of task formulation on neural network features* in *International Conference on Artificial Intelligence and Statistics* (2023), 11563–11582.
3. Argyropoulos, C., Etheridge, A., Sakhanenko, N. & Galas, D. Modeling bias and variation in the stochastic processes of small RNA sequencing. *Nucleic Acids Res.* **45,** e104–e104 (2017).
4. Harris, T. E. *et al. The theory of branching processes* (Springer Berlin, 1963).
5. Lalam, N. A quantitative approach for polymerase chain reactions based on a hidden Markov model. *J. Math. Biol.* **59,** 517–533 (2009).
6. Gevertz, J. L., Dunn, S. M. & Roth, C. M. Mathematical model of real-time PCR kinetics. *Biotechnol. Bioeng.* **92,** 346–355 (2005).
7. Stolovitzky, G. & Cecchi, G. Efficiency of DNA replication in the polymerase chain reaction. *Proc. Natl. Acad. Sci.* **93,** 12947–12952 (1996).
8. Untergasser, A. *et al.* Primer3Plus, an enhanced web interface to Primer3. *Nucleic Acids Research* **35,** W71–W74 (2007).
9. Zuker, M. Mfold Web Server for Nucleic Acid Folding and Hybridization Prediction. *Nucleic Acids Res.* **31,** 3406–3415 (2003).
