# Supplementary material for: Predicting sequence-specific amplification efficiency in multi-template PCR with deep learning
Source: Nat Commun. 2025 Oct 16;16:9187. doi: 10.1038/s41467-025-64221-4 (PMC12533003; doi:10.1038/s41467-025-64221-4)
Supplement: Supplementary file 2 — Reporting Summary [file 41467_2025_64221_MOESM2_ESM.pdf]

nature portfolio

Corresponding author(s): Robert Grass, Karsten Borgwarddt

Last updated by author(s): Jul 28, 2025

Reporting Summary

Nature Portfolio wishes to improve the reproducibility of the work that we publish. This form provides structure for consistency and transparency in reporting. For further information on Nature Portfolio policies, see our Editorial Policies and the Editorial Policy Checklist.

Statistics

For all statistical analyses, confirm that the following items are present in the figure legend, table legend, main text, or Methods section.

n/a	Confirmed	
☐	☒	The exact sample size (n) for each experimental group/condition, given as a discrete number and unit of measurement
☐	☒	A statement on whether measurements were taken from distinct samples or whether the same sample was measured repeatedly
☐	☒	The statistical test(s) used AND whether they are one- or two-sided<br>*Only common tests should be described solely by name; describe more complex techniques in the Methods section.*
☒	☐	A description of all covariates tested
☐	☒	A description of any assumptions or corrections, such as tests of normality and adjustment for multiple comparisons
☐	☒	A full description of the statistical parameters including central tendency (e.g. means) or other basic estimates (e.g. regression coefficient) AND variation (e.g. standard deviation) or associated estimates of uncertainty (e.g. confidence intervals)
☐	☒	For null hypothesis testing, the test statistic (e.g. F, t, r) with confidence intervals, effect sizes, degrees of freedom and P value noted<br>*Give P values as exact values whenever suitable.*
☒	☐	For Bayesian analysis, information on the choice of priors and Markov chain Monte Carlo settings
☒	☐	For hierarchical and complex designs, identification of the appropriate level for tests and full reporting of outcomes
☒	☐	Estimates of effect sizes (e.g. Cohen's d, Pearson's r), indicating how they were calculated

Our web collection on statistics for biologists contains articles on many of the points above.

Software and code

Policy information about availability of computer code

Data collection	Sequencing data was collected on an iSeq 100 sequencer by Illumina, running iSeq 100 Software System Suite v3.1. qPCR data was collected on a LightCycler 480 II by Roche, running LightCycler 480 software 1.5.1.
Data analysis	Data analysis used BBMap (v39.01) for read mapping, as well as custom code implemented in Python (v3.9.7) for deep learning and motif extraction. This custom code uses the Python packages numpy (v1.26.4), pandas (v2.2.3), matplotlib (v3.9.4), torch (v2.0.1), torchmetrics (v0.7.2), scikit-learn (v1.2.2), seaborn (v0.12.2), logomaker (v0.8), captum (v0.6.0), scipy (v1.10.1), pytorch-lightning (v1.5.9), and plotly (v5.20.0). The custom code has been publicly deposited at https://github.com/BorgwardtLab/PCR-bias (DOI: 10.5281/zenodo.16528097).

For manuscripts utilizing custom algorithms or software that are central to the research but not yet described in published literature, software must be made available to editors and reviewers. We strongly encourage code deposition in a community repository (e.g. GitHub). See the Nature Portfolio guidelines for submitting code & software for further information.

Data

Policy information about availability of data

All manuscripts must include a data availability statement. This statement should provide the following information, where applicable:

- Accession codes, unique identifiers, or web links for publicly available datasets
- A description of any restrictions on data availability
- For clinical datasets or third party data, please ensure that the statement adheres to our policy

The experimental sequencing data generated in this study has been deposited in the European Nucleotide Archive under accession code PRJEB77604 [https://

www.ebi.ac.uk/ena/browser/view/PRJEB77604]. Literature sequencing datasets are available from Gimpel et al.20 (European Nucleotide Archive, PRJEB65931 [https://www.ebi.ac.uk/ena/browser/view/PRJEB65931]), Koch et al.49 (European Nucleotide Archive, PRJEB35217 [https://www.ebi.ac.uk/ena/browser/view/PRJEB35217]), Erlich et al.22 (European Nucleotide Archive, PRJEB19305 [https://www.ebi.ac.uk/ena/browser/view/PRJEB19305] and PRJEB19307 [https://www.ebi.ac.uk/ena/browser/view/PRJEB19307]), Song et al.50 (Figshare, 16727122 [https://doi.org/10.6084/m9.figshare.16727122.v2], 17193128 [https://doi.org/10.6084/m9.figshare.17193128.v1], and 18515045 [https://doi.org/10.6084/m9.figshare.18515045.v1]), Gao et al.19 (pers. communication), and Choi et al.18 (European Nucleotide Archive, PRJNA555140 [https://www.ebi.ac.uk/ena/browser/view/PRJNA555140]). Source data are provided with this paper.

The code used to develop the model, perform the analyses and generate results in this study is publicly available and has been deposited in GitHub at github.com/BorgwardtLab/PCR-bias, under BSD 3-Clause license. The specific version of the code associated with this publication is archived in Zenodo and is accessible via https://doi.org/10.5281/zenodo.16528097 [https://doi.org/10.5281/zenodo.16528097].68

Research involving human participants, their data, or biological material

Policy information about studies with human participants or human data. See also policy information about sex, gender (identity/presentation), and sexual orientation and race, ethnicity and racism.

Reporting on sex and gender	Sex and gender were not considered in this study.
Reporting on race, ethnicity, or other socially relevant groupings	Race, ethnicity, or other socially relevant groupings were not considered in this study.
Population characteristics	Population characteristics were not considered in this study.
Recruitment	No participants were recruited for this study.
Ethics oversight	No ethics approval was required for this study.

Note that full information on the approval of the study protocol must also be provided in the manuscript.

Field-specific reporting

Please select the one below that is the best fit for your research. If you are not sure, read the appropriate sections before making your selection.

- [x] Life sciences
- [ ] Behavioural & social sciences
- [ ] Ecological, evolutionary & environmental sciences

For a reference copy of the document with all sections, see nature.com/documents/nr-reporting-summary-flat.pdf

Life sciences study design

All studies must disclose on these points even when the disclosure is negative.

Sample size	Sample sizes of the oligonucleotide pools were chosen with respect to the required diversity for training of deep learning models, and the available sequencing capacity of the iSeq 100 sequencer. To enable accurate estimation of coverage, an average of 100 reads per sequence are desirable. This is standard for many DNA data storage experiments (e.g., see). The iSeq 100 outputs around 5-6 million reads, thus at five samples per sequencing run, oligonucleotide pools with 12000 random sequences were chosen, enabling sequencing depths of around 100x while maximizing available sequence diversity. Literature datasets were used as is.
Data exclusions	During post-processing of the sequencing data, individual sequences which appeared in fewer than two sequencing datasets in a given experimental series were excluded. This was necessary, as estimation of initial coverage and amplification efficiency requires abundance information at two cycle counts at a minimum. In addition, the analysis of the verification pool was limited to the sequences selected by the simple model in order to simplify figures, as outlined in Supplementary Note 2.
Replication	Replication was performed both internally and externally. Internally, selected sequences' amplification efficiencies were verified by an orthogonal method, using qPCR, as well as using a verification pool, that was independently synthesized and re-measured across a larger cycle count. Externally, an independently composed and synthesized oligonucleotide pool was amplified in an external laboratory, in order to test the performance of the deep learning models. All attempts at replication were successfull.
Randomization	Sequences were allocated into the oligonucleotide pools randomly, except for the GC-constrained pool, in which only random sequences with a fixed GC content of 50% were allocated. The sequences used in qPCR verification and the verification pool were not randomly chosen, and instead selected based on their estimated amplification efficiency of the GCall experiment. This was necessary to highlight and test the reproducibility of the differences in amplification efficiency.
Blinding	During external validation, the investigators performing the experiments and performing the property prediction via deep learning models were blinded to the identity of the sequences and whether motifs were explicitly introduced into them. Otherwise, blinding is not relevant to the study, as individual sequences in the pool cannot be handled independently, thus precluding any deliberate introduction of bias.

Reporting for specific materials, systems and methods

We require information from authors about some types of materials, experimental systems and methods used in many studies. Here, indicate whether each material, system or method listed is relevant to your study. If you are not sure if a list item applies to your research, read the appropriate section before selecting a response.

Materials & experimental systems

n/a	Involved in the study
☒	☐ Antibodies
☒	☐ Eukaryotic cell lines
☒	☐ Palaeontology and archaeology
☒	☐ Animals and other organisms
☒	☐ Clinical data
☒	☐ Dual use research of concern
☒	☐ Plants

Methods

n/a	Involved in the study
☒	☐ ChIP-seq
☒	☐ Flow cytometry
☒	☐ MRI-based neuroimaging

Plants

Seed stocks	*Report on the source of all seed stocks or other plant material used. If applicable, state the seed stock centre and catalogue number. If plant specimens were collected from the field, describe the collection location, date and sampling procedures.*
Novel plant genotypes	*Describe the methods by which all novel plant genotypes were produced. This includes those generated by transgenic approaches, gene editing, chemical/radiation-based mutagenesis and hybridization. For transgenic lines, describe the transformation method, the number of independent lines analyzed and the generation upon which experiments were performed. For gene-edited lines, describe the editor used, the endogenous sequence targeted for editing, the targeting guide RNA sequence (if applicable) and how the editor was applied.*
Authentication	*Describe any authentication procedures for each seed stock used or novel genotype generated. Describe any experiments used to assess the effect of a mutation and, where applicable, how potential secondary effects (e.g. second site T-DNA insertions, mosiacism, off-target gene editing) were examined.*
